# Does illicit drug use increase stroke risk? A systematic review, meta-analyses, and Mendelian randomization analysis

**DOI:** 10.1177/17474930261418926

**Published:** 2026-01-21

**Authors:** Megan Ritson, Hugh S Markus, Eric L Harshfield

**Affiliations:** Department of Clinical Neurosciences, University of Cambridge, Cambridge, UK

**Keywords:** Illicit drugs, MR analysis, meta-analysis, stroke, substance-related disorders, systematic review

## Abstract

**Background:**

Epidemiological evidence suggests associations between substance use disorders and risk of stroke, but whether these are due to confounding or are true causal relationships remains uncertain.

**Aims:**

To meta-analyze the observational evidence on illicit substance use and stroke risk and apply Mendelian randomization (MR) to evaluate potential causal effects of substance dependence on stroke subtypes.

**Methods:**

We conducted a systematic review and meta-analysis of studies reporting associations between illicit drug use and stroke (PROSPERO registration—CRD420251053702). The meta-analysis included 32 studies comprising more than 100 million total participants across administrative, hospital-based, and population-based datasets. Pooled odds ratios (ORs) were estimated using multivariate random-effects models for ischemic and hemorrhagic subtypes. We then performed two-sample MR using genome-wide association study summary statistics to examine associations between seven drug exposures and all stroke, ischemic and hemorrhagic stroke, and ischemic stroke subtypes.

**Results:**

Meta-analysis demonstrated significant associations of cannabis (OR = 1.37, 95% confidence interval (95% CI) = 1.14–1.65), cocaine (OR = 1.96; 95% CI = 1.27–3.01), and amphetamines (OR = 2.22, 95% CI = 1.40–3.53) with increased stroke risk, while no significant association was observed for opioids. Findings for cannabis showed some heterogeneity and small-study effects. MR analyses revealed that cannabis use disorder was associated with any stroke (OR = 1.11 [1.01–1.51]) and large artery stroke (OR = 1.35, 95% CI = 1.01–1.80), and cocaine dependence was associated with cardioembolic stroke (OR = 1.08, 95% CI = 1.02–1.14) and intracerebral hemorrhage (OR = 1.38, 95% CI = 1.15–1.65). Genetically predicted substance use disorder overall was associated with any stroke (OR = 1.33, 95% CI = 1.02–1.72) and intracerebral hemorrhage (OR = 7.79, 95% CI = 3.46–17.54). Problematic and dependent alcohol use was linked to large artery and cardioembolic stroke, whereas nicotine dependence showed no significant associations.

**Conclusion:**

Our findings provide consistent observational and genetic evidence that several forms of substance misuse increase stroke risk, particularly cocaine, amphetamines, and cannabis. These findings suggest important public health implications for prevention strategies targeting substance use disorders to mitigate stroke risk.

## Graphical abstract

**Graphical abstract:**
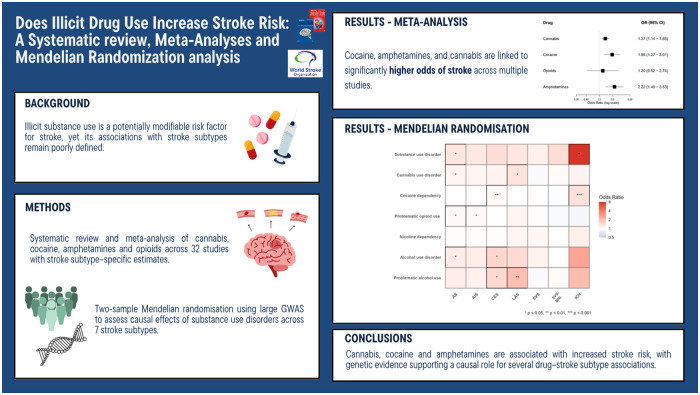

## Introduction

Stroke is a leading global health challenge, ranking as the third leading cause of death and disability combined, with the global burden rising substantially by 70% from 1990 to 2021. Most of this burden is attributable to modifiable risk factors, highlighting the importance of identifying preventable determinants such as substance misuse.^1^ Substance misuse and dependence, encompassing both legal and illicit drugs, represent a major global public health concern, substantially contributing to worldwide morbidity and mortality.^2^ In March 2024, 8.8% of adults aged 16 to 59 years in England and Wales reported using any drug in the past year, equating to approximately 2.9 million individuals.^3^ Recent data from the United States reports that over half of all those aged over 12 have used illicit drugs at least once.^4^ The use of illicit drugs—such as cocaine, cannabis, and opiates—have been linked to a plethora of adverse health outcomes, including cardiovascular diseases.^5,6^ Emerging evidence suggests that abuse of these substances may influence stroke risk, with studies reporting specific effects on stroke subtypes.^7–12^ However, observational studies investigating substance misuse and stroke risk are heterogeneous in exposure definitions, outcome classification, and study design, producing a fragmented evidence base.

One problem in interpreting epidemiological associations is determining whether they are causal or could be explained by confounding. For example, substance abuse might be associated with other unmeasured behaviors that also increase stroke risk. Mendelian randomization (MR) can help to overcome these limitations by using genetic variants as instrumental variables to assess causal effects.^13^ Previous MR studies have focused largely on legal substances such as alcohol and tobacco use,^14–17^ primarily investigating consumption rather than dependence. A few MR studies have investigated cannabis dependency; however, they were conducted prior to the availability of larger genome-wide association studies (GWAS).^18,19^ To date, there are no published MR analyses investigating cocaine or opioid dependency or broader drug-use disorders. Therefore, the causal relationships between illicit substances and stroke subtypes remain unclear.

To address these gaps, we first conducted a systematic review and meta-analysis of existing epidemiological evidence on substance misuse and stroke risk. Building upon these findings, we performed a two-sample MR analysis to examine whether the epidemiologic associations we identified are likely to be causal effects and whether any associations differed based on stroke subtypes.

## Methods

### Systematic review

#### Search strategy

This systematic review followed the Preferred Reporting Items for Systematic reviews and Meta-Analyses (PRISMA) guidelines^20^ and was prospectively registered with PROSPERO (CRD420251053702) on 20 May 2025. Systematic searches of PubMed, Embase, Scopus, and Web of Science were performed from database inception to 26 May 2025 without restriction on publication date. Search strategies were refined with a professional librarian (full syntax and PRISMA 2020 checklist in Supplementary Appendix).

Following the removal of duplicated studies, titles and abstracts were independently screened by two independent reviewers blinded to each other’s assessments (M.R. and E.L.H.) followed by full-text assessment. Any discrepancies were decided by discussion. The online platform Rayyan was used to manage study selection.

#### Inclusion and exclusion selection criteria

Eligible studies were original, peer-reviewed English-language publications reporting the primary outcome including ischemic stroke, hemorrhagic stroke, unspecified stroke, or cerebrovascular events, as defined by each study. For the meta-analysis of observational studies, exposure was defined as confirmed use of illicit substances (cannabis, cocaine, amphetamines, or opioids), based on self-report, clinical diagnosis, or toxicology testing, depending on the study. When referring to this analysis, “use” refers to consumption regardless of severity or frequency. Where available, information on frequency and duration was also extracted. Included study designs consisted of cohort, case-control and cross-sectional studies. Studies were excluded if they were case reports or case series, conference abstracts, literature reviews, animal studies, in vitro studies, studies on fetal outcomes, or exclusively HIV+ populations.

#### Data extraction and quality assessment

For each included study, data were extracted using a standardized data extraction template in Microsoft Excel. Extracted information included participant age range, year, country, study design, and adjusted risk estimates (relative risk, hazard ratio, or odds ratio [OR]) with 95% confidence intervals (CI) or standard errors (SEs) as reported. Only adjusted ORs were eligible for inclusion in the quantitative meta-analysis; unadjusted ORs and other effect measures were excluded. To address potential confounding, only studies reporting adjusted ORs accounting for key confounders such as smoking, alcohol use, hypertension, and concurrent substance misuse were included. Where only an OR and *P*-value were reported, 95% CIs were estimated using log(OR), assuming a normal distribution. Study quality was assessed using the Newcastle–Ottawa Scale.^21^

#### Statistical analyses

For studies reporting multiple relevant outcomes, each outcome was included in the quantitative analysis. Due to heterogeneity, outcomes with similar definitions were grouped. Pooled ORs were estimated using multivariate random-effects meta-analysis using the R package *metafor* v4.8-0, accounting for the correlation between multiple outcomes reported by the same study. Correlation coefficients of 0.2, 0.5, and 0.8 were tested to assess robustness. As a confirmatory approach, separate univariate pooled risk estimates per stroke subtype were also calculated using the DerSimonian and Laird random-effects model for each drug type. Within each exposure, a pooled estimate from the multivariate random-effects meta-analysis was only calculated for outcomes with ⩾3 studies. To assess potential temporal bias, a sensitivity analysis excluding studies published before 2010 was conducted using the same multivariate approach. Subgroup analyses were performed restricted to studies that reported results in populations under 55 years of age, for which pooled estimates were calculated for all stroke types combined. We also conducted a sensitivity analysis restricted to studies that explicitly reported recent substance misuse, defined as exposure within the preceding 72 h or at the time of hospital admission, to assess the influence of exposure timing on pooled estimates. Variance was calculated using 95% CIs for each estimate.

Sensitivity analyses included Cochran’s *Q* and the *I*^2^ statistics to assess heterogeneity. Publication bias was evaluated with funnel plots, and small-study effects were assessed using multi-level meta-regression of effect size on SE. Publication bias was also formally assessed with Egger’s and Begg’s tests and trim-and-fill correction for meta-analyses with three or more studies. Funnel plots with imputed studies and adjusted pooled estimates from trim-and-fill analysis are reported alongside unadjusted estimates. Statistical analyses were conducted in R v.4.3.0.

### Mendelian randomization analysis

#### Study design

**Figure 1.**
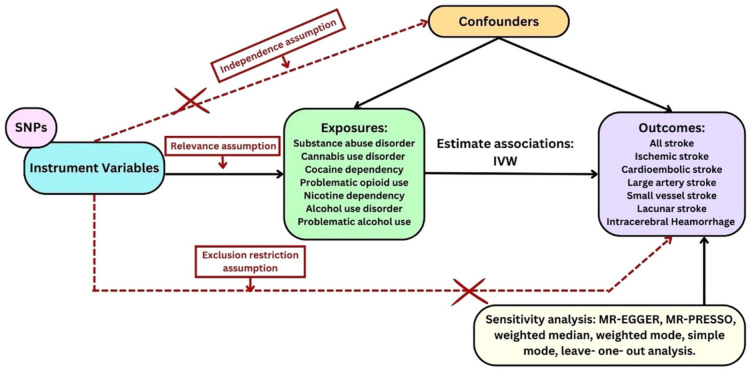
Mendelian randomization study design and key assumptions in investigating substance use disorders and stroke subtypes.

A two-sample MR analysis was conducted using publicly available GWAS summary statistics following established MR reporting guidelines.^22^ Genetic variants strongly associated with the exposures of interest were used as instrumental variables, satisfying three key assumptions as shown in Figure 1. Mendelian randomization was used to assess the causal effects of seven exposures, including substance use disorder (SUD), problematic alcohol use (PAU), alcohol use disorder (AUD), problematic opioid use (POU), cannabis use disorder (CUD), cocaine dependence (CD), and nicotine dependence (ND). For these analyses, the term “substance use disorder” refers to genetic liability to problematic or dependent use. The following stroke outcomes were assessed: any stroke (AS), any ischemic stroke (AIS), large artery stroke (LAS), cardioembolic stroke (CES), small vessel stroke (SVS), SVS-MRI, and intracerebral hemorrhage (ICH).

#### GWAS summary statistics

Exposure GWAS summary statistics were obtained for all substance traits^23–28^ (Table S13 and Supplementary Methods). Outcome datasets included GWAS summary statistics for five stroke subtypes.^29–31^ For SVS, we used two datasets: first, SVS from the larger MEGASTROKE (N = 11,710 cases) and second, a dataset of MRI-confirmed SVS.^30^ Both represent the same underlying phenotype, but in MEGASTROKE, much of SVS stroke subtyping was performed only using computed tomography (CT) imaging, and this has been shown to have a lower sensitivity and specificity, while MRI has a high sensitivity for SVS. All analyses were restricted to European-ancestry populations to minimize population stratification bias.

#### Instrumental variable selection

Instrumental variables were selected based on genome-wide significance thresholds (*P* < 5 × 10^−8^ for PAU, AUD, CUD, POU; *P* < 5 × 10^−7^ for SUD and *P* < 5 × 10^−5^ for ND and CD) from the largest publicly available GWAS summary statistics. Where full GWAS summary statistics were unavailable (POU and CD), the top 10,000 or 100 single nucleotide polymorphisms (SNPs) were used, respectively. Linkage disequilibrium clumping (*r*^2^ ⩾ 0.001, distance ⩽ 10,000 kb) was performed using the 1000 Genomes Project European reference panel to retain independent SNPs. Instrument strength was assessed by calculating *F*-statistics, with values > 10 considered sufficiently strong to reduce the risk of weak instrument bias.

#### Statistical analysis

SNPs reaching genome-wide significance were harmonized with outcome data to align alleles. The primary MR analysis used an inverse-variance weighted (IVW) random-effects model to estimate causal effects.^32^ Sensitivity analyses included MR-Egger regression, weighted median and simple and weighted mode-based estimators, and leave-one-out analysis to assess the influence of individual SNPs. Horizontal pleiotropy was evaluated using the MR-PRESSO and Steiger filtering to confirm the causal direction from exposure to outcome. Multiple testing was accounted for using a false discovery rate (FDR) threshold of *q* < 0.05. All analyses were conducted in R version 4.4.3 using the TwoSampleMR package (v. 0.16.14) and MR-PRESSO package (v.1.0). Two-sided *P*-values and 95% CIs are presented.

To limit the effect of confounding bias, SNPs were cross-checked against dbSNP for associations with known confounders (e.g. age, sex, body mass index (BMI), blood pressure, socioeconomic factors). SNPs that had significant associations with confounders were removed to satisfy MR assumptions.

## Results

### Systematic review

#### Literature search and inclusion

**Figure 2.**
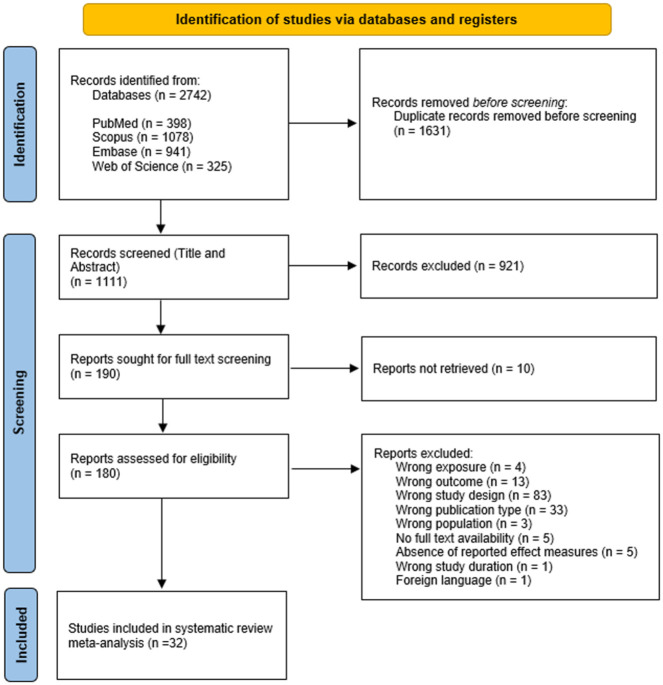
PRISMA flow diagram of study selection process. PRIMSA: Preferred Reporting Items for Systematic reviews and Meta-Analyses.

The search identified 2742 articles (Figure 2). After removing duplicates, 1631 articles remained for screening. After review of the titles and abstracts, 180 of the articles met the criteria for full-text screening, of which 32 studies met the inclusion criteria for the systematic review and meta-analysis. These comprised 14 cohort, 10 case–control, and 8 cross-sectional studies. Across these studies, the meta-analyses pooled 24 cannabis effect estimates from 19 studies, 18 cocaine estimates from 14 studies, 11 amphetamine estimates from 8 studies, and 13 opioid estimates from 10 studies. Participant numbers and study details are provided in Table S1.

#### Risk of bias

The Newcastle–Ottawa Scale was used to assess risk of bias, with studies rated as poor (0–2), fair (3–5), good (6–7), or high quality (8–9). Two studies^3,33,34^ were excluded due to poor quality (Tables S2 to S4). Funnel plot analysis was conducted to visually assess publication bias (Figure S1). As most subtype-specific analyses included fewer than 10 studies, formal assessments of publication bias using Egger’s and Begg’s tests, as well as trim-and-fill analyses, were conducted only for overall stroke (Figure S2). Egger’s and Begg’s tests indicated potential small-study effects for cannabis (Egger *p* = 4.16×10^−6^) but not for amphetamines, cocaine, or opioids (Egger *p* ⩾ 0.04; Begg *p* ⩾ 0.54). Trim-and-fill analyses imputed five studies for cannabis, yielding a slightly lower pooled effect (OR = 1.12, 95% CI = 1.02–1.23), which remained statistically significant, whereas no studies were imputed for amphetamines, cocaine, or opioids (Tables S5 and S6).

#### Cannabis and risk of stroke

**Figure 3.**
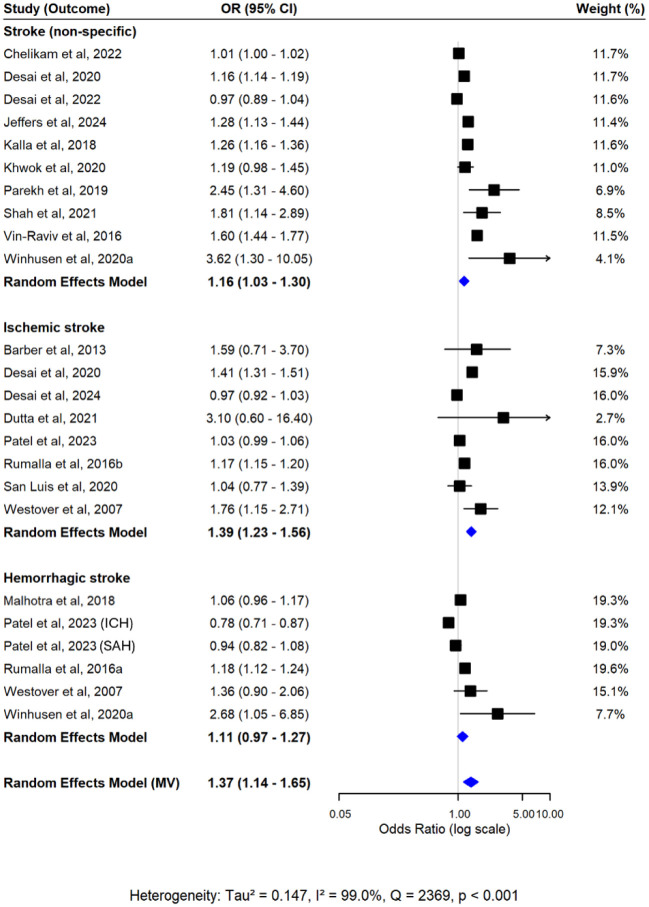
Forest plot from multivariate random-effects meta-analysis of cannabis use and stroke risk. Pooled estimates for each stroke subtype and the overall effect were estimated simultaneously within a single model, accounting for within-study correlations. Squares represent study-specific effect estimates, and diamonds represent the pooled multivariate estimates.

Meta-analysis of 24 cannabis–stroke effect estimates showed a significant association (OR = 1.37, 95% CI = 1.14–1.65) (Figure 3). Subgroup analysis found pooled ORs of 1.16 (95% CI = 1.03–1.30) for non-specific stroke, 1.39 (95% CI = 1.23–1.56) for ischemic stroke, and no significant association for hemorrhagic stroke (OR = 1.11, 95% CI = 0.97–1.27). Univariate random-effects analyses confirmed these findings (Figure S3; Tables S7 and S8). Heterogeneity was found to be substantial (*I*^2^ = 99%), and multivariate regression indicated small-study and between-study effects (Table S9).

#### Cocaine use and stroke risk

**Figure 4.**
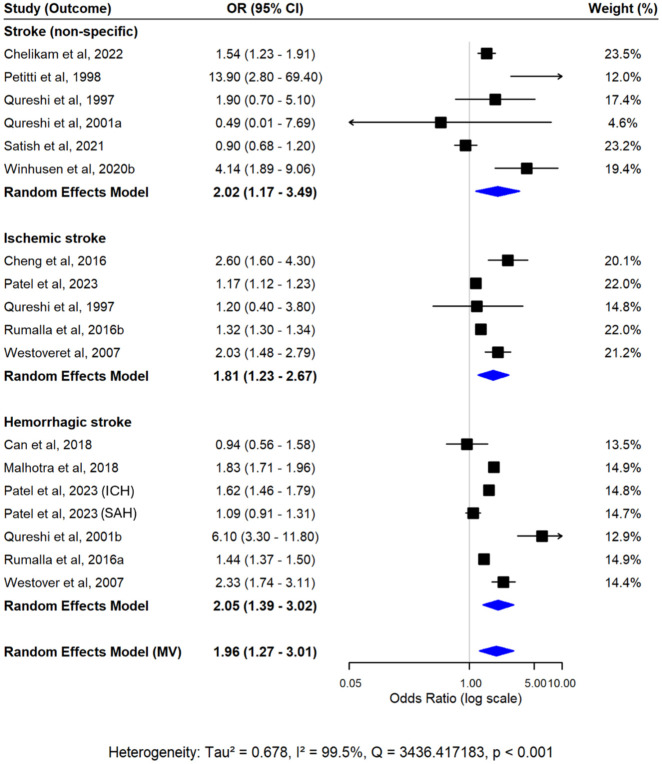
Forest plot from multivariate random-effects meta-analysis of cocaine use and stroke risk. Pooled estimates for each stroke subtype and the overall effect were estimated simultaneously within a single model, accounting for within-study correlations. Squares represent the study-specific effect estimates, and diamonds represent the pooled multivariate estimates.

Pooled meta-analysis of 18 cocaine-stroke effect estimates found a strong association (OR 1.96, 95% CI = 1.27–3.01) (Figure 4). Subtype analyses showed significantly increased risks for all stroke types: non-specific stroke (OR = 2.02, 95% CI = 1.17–3.49), ischemic stroke (OR = 1.81, 95% CI = 1.23–2.67), and hemorrhagic stroke (OR = 2.05, 95% CI = 1.39–3.02). Results from univariate random-effects meta-analyses showed results consistent with the primary multivariate analysis (Figure S4; Tables S7 and S8). The analysis demonstrated substantial between-study heterogeneity (*I*^2^ = 99.5%), and regression results from the multivariate model indicated the presence of small-study and between-study effects (Table S9).

#### Amphetamine use and risk of stroke

**Figure 5.**
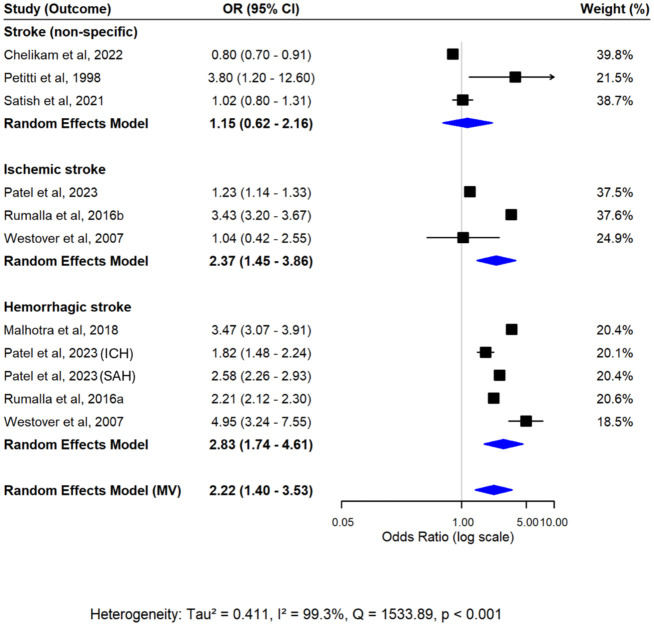
Forest plot from multivariate random-effects meta-analysis of amphetamine use and stroke risk. Pooled estimates for each stroke subtype and the overall effect were estimated simultaneously within a single model, accounting for within-study correlations. Squares represent study-specific effect estimates, and diamonds represent the pooled multivariate estimates.

The meta-analysis on 11 effect estimates of amphetamine use and stroke risk revealed a significant positive association overall (OR = 2.22, 95% CI = 1.40–3.53) (Figure 5). Subgroup analyses by stroke type also found significant associations for ischemic stroke (OR = 2.37, 95% CI = 1.45–3.86) and hemorrhagic stroke (OR = 2.83, 95% CI = 1.74–4.61). For non-specific stroke, a pooled OR of 1.15 (95% CI = 0.62–2.16) was calculated, but this association was non-significant. There was substantial heterogeneity observed across studies (*I*^2^ = 99.3%) and regression results from the multivariate model indicated the presence of between-study effects (Table S9). Results from univariate random-effects meta-analyses were largely consistent with the primary multivariate analysis, although the association for ischemic stroke was not statistically significant (Figure S5; Tables S7 and S8).

#### Opioid use and stroke risk

**Figure 6.**
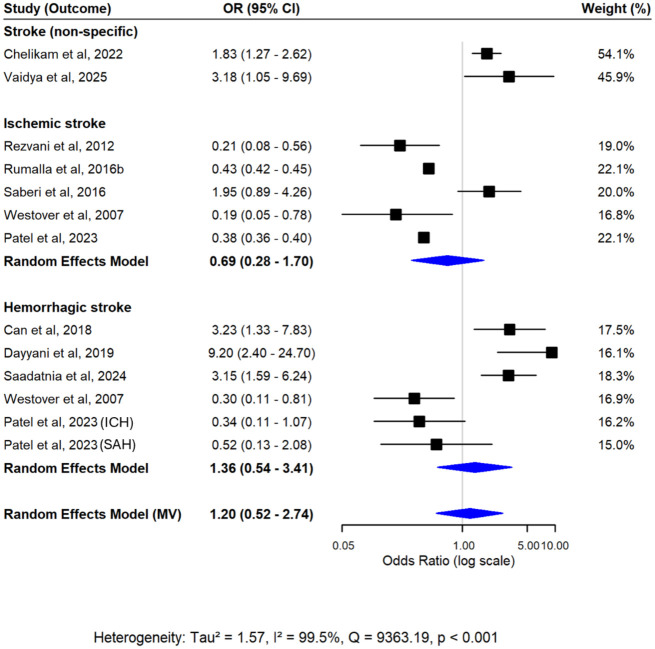
Forest plot from multivariate random-effects meta-analysis of opioid use and stroke risk. Pooled estimates for each stroke subtype and the overall effect were estimated simultaneously within a single model, accounting for within-study correlations. A pooled estimate for non-specific stroke was not obtained due to the presence of fewer than three studies. Squares represent the study-specific effect estimates, and diamonds represent the pooled multivariate estimates.

The meta-analysis on 12 effect estimates of opioid use and stroke risk showed no statistically significant association overall, with a pooled OR of 1.20 (95% CI = 0.52–2.74) (Figure 6). A pooled analysis for non-specific stroke was not conducted, as there were fewer than three studies available. For the remaining subtypes, no significant association was found for ischemic stroke (OR = 0.69, 95% CI = 0.28–1.70) or for hemorrhagic stroke (OR = 1.36, 95% CI = 0.54–3.41). Heterogeneity between studies was substantial (*I*^2^ = 99.5%), and regression results from the multivariate model did not indicate significant small-study or between-study effects (Table S9). Results from univariate random-effects meta-analyses showed a statistically significant association for the ischemic stroke subgroup, which was not observed in the multivariate analysis (Figure S6; Tables S7 and S8).

#### Drug use and stroke risk in younger people

**Figure 7.**
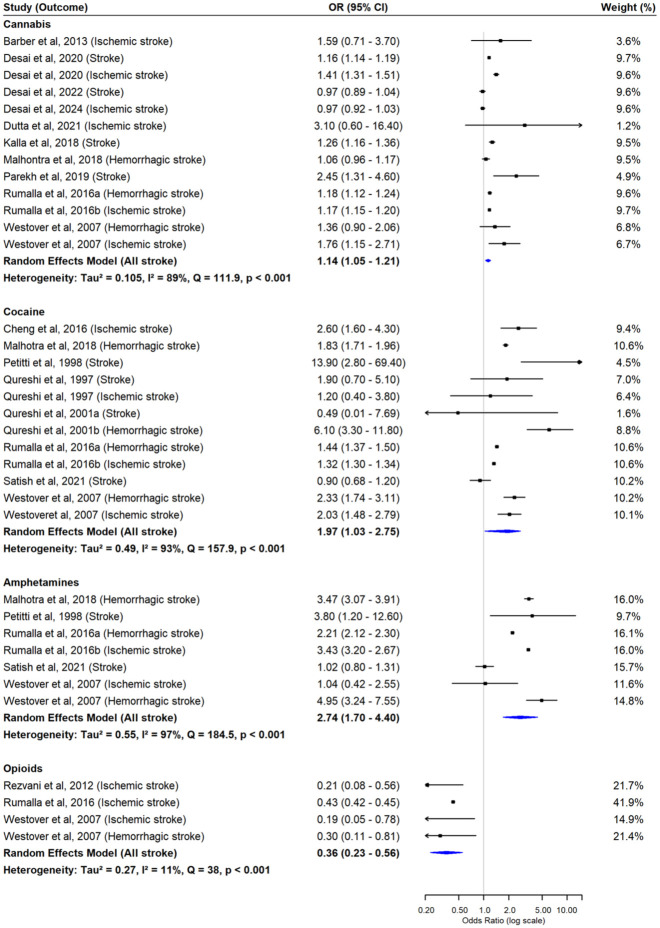
Forest plot from multivariate random-effects meta-analysis of stroke risk in younger populations. Pooled estimates for each illicit drug and the overall risk of stroke were estimated simultaneously within a single model, accounting for within-study correlations. Squares represent the study-specific effect estimates, and diamonds represent the pooled multivariate estimates.

Meta-analysis restricted to individuals under 55 years demonstrated significantly increased stroke risk associated with cannabis (OR = 1.14, 95% CI = 1.05–1.21), cocaine (OR = 1.97, 95% CI = 1.03–2.75), and amphetamines (OR = 2.74, 95% CI = 1.70–4.40) (Figure 7). In contrast, opioid use in this population was associated with a statistically significant decrease in stroke risk (OR = 0.36, 95% CI = 0.23–0.56). There was substantial heterogeneity observed across studies for cannabis, cocaine, and amphetamines (*I*^2^ > 89%), although not for opioids (*I*^2^ = 11%) (Table S10). Meta-regression results indicated evidence of between-study effects for cannabis, small-study effects for cocaine, both small and between-study effects for amphetamines, and no indication of either effect for opioids (Table S11).

#### Sensitivity analysis

Sensitivity analyses excluding studies published before 2010 showed results consistent with the primary analysis for all drug types (Table S12). Effect estimates were similar in both size and direction, with overlapping CIs, and statistical significance remained unchanged.

A further sensitivity analysis restricted to studies reporting recent illicit drug use showed pooled estimates broadly consistent with the main analysis, with associations for cocaine and amphetamines remaining significant, and opioid use showed a higher and statistically significant estimate. For cannabis, the direction of effect was the same but no longer statistically significant. Heterogeneity remained unchanged (98.7%) (Figure S7).

### Mendelian randomization analysis of substance misuse and stroke and its subtypes

**Figure 8.**
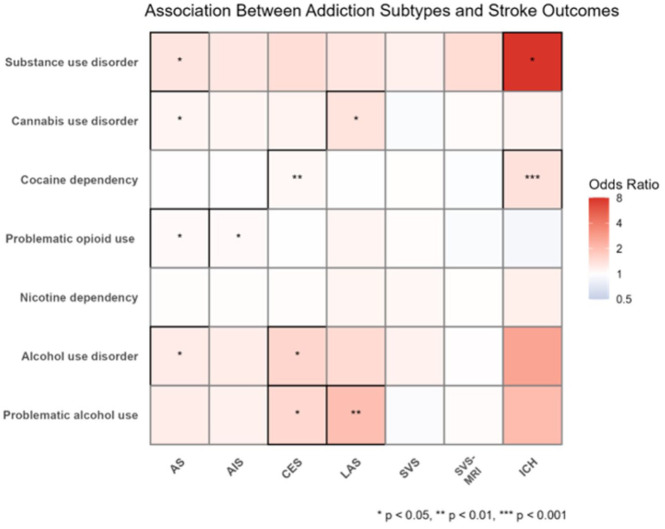
Mendelian randomization results showing causal estimates for the association of genetically predicted substance use disorders with stroke and its subtypes. Colors show the magnitude and direction of the associated odds ratio (OR) using the inverse-variance weighted Mendelian randomization approach. Significance of associations (false discovery rate *q* < 0.05) are indicated with asterisks (*) (*P* < 0.05, **P < 0.01, ***P < 0.001). AS indicates any stroke; AIS, any ischemic stroke; CES, cardioembolic stroke; LAS, large artery stroke; SVS, small vessel stroke; SVS-MRI, small vessel stroke (MRI-confirmed); ICH, intracerebral hemorrhage.

To assess potential causal effects, we conducted Mendelian randomization analyses of genetic liability to drug and alcohol traits on stroke and its subtypes (Figure 8; Figure S8; Table S14). Scatterplots of variant–outcome associations of significant traits (FDR *q* < 0.05) are shown in Figure S9, and results from the leave-one-out analysis are shown in Figure S10.

Genetic liability to SUD was significantly associated with AS (OR 1.33, 95% CI = 1.02–1.72; *P* = 0.018) and most strongly with ICH (OR = 7.79, 95% CI = 3.46–17.54; *P* = 0.033). Specific substance analyses indicated that cocaine use was associated with ICH (OR = 1.38, 95% CI = 1.15–1.65; *P* = 0.006) and cardioembolic stroke (OR = 1.08, 95% CI = 1.02–1.14; *P* = 0.004). CUDs were associated with any stroke (OR = 1.11, 95% CI = 1.01–1.51; *P* = 0.015) and LAS (OR = 1.35, 95% CI = 1.01–1.80; *P* = 0.029). Opiate abuse was associated with any stroke (OR = 1.05, 95% CI = 1.00–1.10; *P* = 0.029) and ischemic stroke (OR = 1.05, 95% CI = 1.00–1.11; *P* = 0.03). We were unable to assess associations with amphetamine use due to a lack of suitable genetic instruments.

PAU was associated with cardioembolic stroke (OR = 1.51, 95% CI = 1.05–2.17; *P* = 0.035) and LAS (OR = 2.00, 95% CI = 1.23–3.27; *P* = 0.009), whereas AUD was associated with any stroke (OR = 1.24, 95% CI = 1.01–1.51; *P* = 0.037) and cardioembolic stroke (OR = 1.56, 95% CI = 1.09–2.24; *P* = 0.035).

No significant associations of nicotine dependency were found with any stroke subtype, nor were any of the substance use exposures significantly associated with SVS or lacunar stroke (LS) (*q* < 0.05).

Sensitivity analyses using MR-Egger, weighted median, mode-based estimators, and MR-PRESSO are reported (full results in Table S14). While some methods have effect sizes and *P*-values that differ from the IVW estimates, there was no evidence of bias. MR-Egger detected no directional pleiotropy, the MR-PRESSO global test found no horizontal pleiotropy or outliers, and Steiger filtering indicated no reverse causality.

## Discussion

In this comprehensive analysis of multiple illicit drugs and stroke, we show consistent evidence that cannabis, cocaine, and amphetamines are associated with increased stroke risk, with MR analyses suggesting potential associations between genetic liability to several illicit drug exposures and stroke subtypes.

Cannabis use was associated with increased stroke risk in our meta-analysis, consistent with prior systematic reviews that reported elevated risk, although we found substantial heterogeneity.^35,36^ While previous analyses did not detect differences between ischemic and hemorrhagic subtypes,^35^ our findings suggest that the excess risk is primarily attributable to ischemic stroke. Mechanisms suggested to link cannabis with stroke include cerebral vasoconstriction, impaired cerebral vasomotor function, and fluctuations in blood pressure.^37^ It has been suggested these may largely relate to the potential of cannabis to induce sympathetic stimulation and decrease parasympathetic activity.^37^ Cannabis has also been associated with increased platelet aggregation and a possible prothrombotic effect.^38^ Our MR analyses further support causality, showing that genetically predicted CUD increases the risk of both AS and LAS. These findings align with previous MR studies using smaller datasets;^17,18^ however, our analysis benefits from substantially larger GWAS, providing greater statistical power and increasing confidence in the robustness of the findings.

Cocaine showed one of the strongest associations in our meta-analysis, nearly doubling the risk of stroke and increasing the risk for both ischemic and hemorrhagic stroke. This aligns with a previous systematic review reporting elevated stroke risk among cocaine users, particularly in younger populations, although differences between ischemic and hemorrhagic stroke were not quantified.^39^ Our MR analyses similarly linked genetically predicted CD with both CES and ICH. Mechanistically, cocaine has been reported to result in cerebral vasospasm, perhaps due to its sympathomimetic effect and an increase in circulating endothelin 1.^40^ It can also produce sudden elevations in blood pressure and vasospasm and an increased risk of cervical arterial dissection, and, when used chronically, accelerate atherosclerosis.^41^ Together, the consistency between observational and genetic evidence suggests that the strong associations reported previously are unlikely to be explained by acute intoxication events or confounding lifestyle factors but may reflect a direct association between cocaine use and stroke risk.

Amphetamine use was also strongly associated with both ischemic and hemorrhagic stroke in our meta-analysis, showing more than a twofold increase overall and significant effects for both subtypes. One of the larger studies reported a fourfold increase in overall stroke risk among amphetamine users, with hemorrhagic events occurring roughly twice as often as ischemic strokes.^42^ These associations are biologically plausible, as amphetamines can provoke acute surges in blood pressure, as well as cerebral vasoconstriction.^43^ They have also been associated with a necrotising vasculitis^44^ and cardiomyopathy and arrythmias.^37^ Despite these strong epidemiological associations, we were unable to evaluate amphetamine use in our MR analysis due to the lack of suitable GWAS summary statistics. The strength of the observational evidence from the meta-analysis highlights a need for genetic studies to clarify causality, particularly given the apparent high risk for both hemorrhagic and ischemic events.

In our meta-analysis, we found no association between opioid and overall stroke risk. This may reflect a true weak or absent association or heterogeneity across studies. Most reports lacked detail on route and duration, factors that may be critical since duration-dependent use has been associated with increased risk.^45^ It is well recognized that in some cases, IV opiate use is associated with an increased risk of infective endocarditis which can cause stroke, but such cases may be such a small proportion of the overall opiate users that they are not detected in our analyses. Our MR analysis focusing on prescription opioid use identified significant associations with AS and AIS. However, a previous MR of POU and stroke found no significant effect, although this analysis had a smaller sample size.^46^ The lack of epidemiological but modest associations on MR could partly reflect exposure misclassification, while genetic evidence points toward a modest cerebrovascular impact.

Our subgroup analyses focusing on individuals under 55 years of age showed broadly similar associations to the all-age analyses. For cannabis, the observed association is consistent with a previous systematic review in younger populations.^47^ The CIs overlapped with those from the all-age analyses, indicating no statistically significant differences by age group. In contrast, opioid use appeared significantly associated with a decreased stroke risk in the younger subgroup. This likely reflects selection bias and the small sample size, rather than a true protective effect. These findings suggest that younger populations largely align with the all-age associations, although the possibility of differential susceptibility warrants further investigation. Sensitivity analyses restricted to studies capturing recent substance misuse produced estimates broadly consistent with the primary analysis. Cannabis was no longer statistically significant, which may suggest that its association with stroke reflects longer-term rather than recent use, although this cannot be confirmed given the limited data. Opioid estimates were higher, based on only three studies, and should be interpreted cautiously.

No significant associations were observed for any drug exposures with SVS and SVS-MRI in the MR analyses. Observational studies have suggested associations between cocaine and opioid use with white matter hyperintensities and cerebral microbleeds, pointing toward possible small vessel pathology;^48,49^ however, the evidence remains inconclusive for a definitive increase in SVS or SVS-MRI risk.

This study has several strengths. The meta-analysis captured broad observational data and enabled subgroup analyses by stroke subtype. It represents the most comprehensive evaluation of multiple substance misuse types and stroke subtypes. By also performing MR analysis, we were able to gain information on causality, particularly important where epidemiological data may be subject to confounding by other associated environmental exposures which could themselves increase stroke risk.

Limitations should also be acknowledged. For the meta-analysis, substantial heterogeneity (*I*^2^> 95%) arising from differences in study design, populations, and exposure definitions may affect the precision of pooled estimates. Many studies did not report prior stroke history or specify whether events were first-ever or recurrent, and of the 32 studies included in the met-analysis, 27 were conducted in the United States and all cocaine use studies were from the United States, limiting generalizability of findings to other populations and geographic contexts. In addition, the majority of included studies were based on hospital records, which may introduce selection bias and limit generalizability to community-based populations. Exposure definitions and key variables, such as exposure timing and frequency relative to stroke and sex, were inconsistently defined or not reported, with only 14/32 studies explicitly reporting substance use prior to the stroke event. This limits sensitivity analyses based on these factors and highlights the need for future studies to report detailed exposure information. For the MR analyses, restriction to European ancestry may limit generalizability, and for some drug exposures (SUD, ND, CD), instrument thresholds had to be relaxed. CD analyses were further constrained by only the top 100 SNPs, and POU by the top 100,000 SNPs, potentially limiting variant coverage. Nevertheless, sensitivity analyses detected no pleiotropy or heterogeneity, and the overall consistency between the meta-analysis and MR findings supports the robustness of the results.

In summary, these findings demonstrate consistent associations and provide evidence that substance misuse is associated with an increased risk of stroke and provide support for associations with cocaine, amphetamines, and cannabis, although some residual heterogeneity and confounding may remain. Taken together, they emphasize the importance of assessing substance misuse when evaluating stroke risk and the importance of public health measures to reduce substance abuse in reducing stroke risk.

## Supplemental Material

sj-docx-1-wso-10.1177_17474930261418926 – Supplemental material for Does illicit drug use increase stroke risk? A systematic review, meta-analyses, and Mendelian randomization analysis Supplemental material, sj-docx-1-wso-10.1177_17474930261418926 for Does illicit drug use increase stroke risk? A systematic review, meta-analyses, and Mendelian randomization analysis by Megan Ritson, Hugh S Markus and Eric L Harshfield in International Journal of Stroke

## References

[1] Feigin VL Brainin M Norrving B , et al. World stroke organization: global stroke fact sheet 2025. Int J Stroke 2025; 20: 132–144. 39635884 10.1177/17474930241308142 PMC11786524

[2] Shen J Hua G Li C Liu S Liu L Jiao J. Prevalence, incidence, deaths, and disability-adjusted life-years of drug use disorders for 204 countries and territories during the past 30 years. Asian J Psychiatr 2023; 86: 103677. 37348194 10.1016/j.ajp.2023.103677

[3] Office for National Statistics. Drug misuse in England and Wales: year ending March 2024. Published 12 December 2024. https://www.ons.gov.uk/peoplepopulationandcommunity/crimeandjustice/articles/drugmisuseinenglandandwales/yearendingmarch2024 (accessed 8 September 2025).

[4] National Center for Drug Abuse Statistics. Drug abuse statistics. https://drugabusestatistics.org (accessed 8 September 2025).

[5] Gan WQ Buxton JA Frank XS , et al. Risk of cardiovascular diseases in relation to substance use disorders. Drug Alcohol Depend 2021; 229: 109132. 34768052 10.1016/j.drugalcdep.2021.109132

[6] Schulte MT Hser YI. Substance use and associated health conditions throughout the lifespan. Public Health Rev 2014; 35(2): 3. 10.1007/BF03391702 PMC5373082 28366975

[7] Cheng YC Ryan KA Qadwai SA , et al. Cocaine use and risk of ischemic stroke in young adults. Stroke 2016; 47: 918–922. 26965853 10.1161/STROKEAHA.115.011417 PMC6128285

[8] Nolte KB Brass LM Fletterick CF. Intracranial hemorrhage associated with cocaine abuse: a prospective autopsy study. Neurology 1996; 46: 1291–1296. 8628469 10.1212/wnl.46.5.1291

[9] Dutta T Ryan KA Thompson O , et al. Marijuana use and the risk of early ischemic stroke: the stroke prevention in young adults study. Stroke 2021; 52: 3184–3190. 34266309 10.1161/STROKEAHA.120.032811 PMC8478805

[10] Esse K Fossati-Bellani M Traylor A Martin-Schild S. Epidemic of illicit drug use, mechanisms of action/addiction and stroke as a health hazard. Brain Behav 2011; 1: 44–54. 22398980 10.1002/brb3.7 PMC3217673

[11] Saadatnia M Norouzi R Najafi MA , et al. Opioid use disorder and intracerebral hemorrhage in Isfahan, Iran: a case-control study. Front Neurol 2024; 15: 1420675. 10.3389/fneur.2024.1420675 PMC11439711 39350972

[12] Di Napoli M Zha AM Godoy DA , et al. Prior cannabis use is associated with outcome after intracerebral hemorrhage. Cerebrovasc Dis 2016; 41: 248–255. 26820826 10.1159/000443532

[13] Richmond RC Smith GD. Mendelian randomization: concepts and scope. Cold Spring Harb Perspect Med 2022; 12: a040501. 10.1101/cshperspect.a040501 PMC8725623 34426474

[14] Christensen AI Nordestgaard BG Tolstrup JS. Alcohol intake and risk of ischemic and hemorrhagic stroke: results from a Mendelian randomisation study. J Stroke 2018; 20: 218–227. 29886720 10.5853/jos.2017.01466 PMC6007300

[15] Larsson SC Burgess S Michaëlsson K. Smoking and stroke: a Mendelian randomization study. Ann Neurol 2019; 86: 468–471. 31237718 10.1002/ana.25534 PMC6701987

[16] Harshfield EL Georgakis MK Malik R Dichgans M Markus HS. Modifiable lifestyle factors and risk of stroke: a Mendelian randomization analysis. Stroke 2021; 52: 931–936. 33535786 10.1161/STROKEAHA.120.031710 PMC7903981

[17] Millwood IY Walters RG Mei XW , et al. Conventional and genetic evidence on alcohol and vascular disease aetiology: a prospective study of 500,000 men and women in China. Lancet 2019; 393: 1831–1842. 30955975 10.1016/S0140-6736(18)31772-0 PMC6497989

[18] Chen M Lu YL Chen XF Wang Z Ma L. Association of cannabis use disorder with cardiovascular diseases: a two-sample Mendelian randomization study. Front Cardiovasc Med 2022; 9: 966707. 36277767 10.3389/fcvm.2022.966707 PMC9582269

[19] Dong L Xie M Li W , et al. Cannabis use disorder increases risk of large-artery atherosclerotic stroke and migraine with aura through Mendelian randomization study. Sci Rep 2024; 14: 24295. 39414896 10.1038/s41598-024-74754-1 PMC11484857

[20] Page MJ McKenzie JE Bossuyt PM , et al. The PRISMA 2020 statement: an updated guideline for reporting systematic reviews. BMJ 2021; 29: 372. 10.1136/bmj.n71 PMC8005924 33782057

[21] Wells G Shea B O’Connell D , et al. The Newcastle-Ottawa Scale (NOS) for assessing the quality of nonrandomised studies in meta-analyses. http://www.evidencebasedpublichealth.de/download/Newcastle_Ottowa_Scale_Pope_Bruce.pdf (accessed 28 August 2009).

[22] Burgess S Smith GD Davies NM , et al. Guidelines for performing Mendelian randomization investigations: update for summer 2023. Wellcome Open Res 2023; 8: 186. 32760811 10.12688/wellcomeopenres.15555.1 PMC7384151

[23] Zhou H Kember RL Deak JD , et al. Multi-ancestry study of the genetics of problematic alcohol use in over 1 million individuals. Nat Med 2023; 29: 3184–3192. 38062264 10.1038/s41591-023-02653-5 PMC10719093

[24] Quach BC Bray MJ Gaddis NC , et al. Expanding the genetic architecture of nicotine dependence and its shared genetics with multiple traits. Nat Commun 2020; 11: 5562. 33144568 10.1038/s41467-020-19265-z PMC7642344

[25] Cabana-Domínguez J Shivalikanjli A Fernàndez-Castillo N , et al. Genome-wide association meta-analysis of cocaine dependence: shared genetics with comorbid conditions. Prog Neuropsychopharmacol Biol Psychiatry 2019; 94: 109667. 31212010 10.1016/j.pnpbp.2019.109667

[26] Levey DF Galimberti M Deak JD , et al. Multi-ancestry genome-wide association study of cannabis use disorder yields insight into disease biology and public health implications. Nat Genet 2023; 55: 2094–2103. 37985822 10.1038/s41588-023-01563-z PMC10703690

[27] Sanchez-Roige S Fontanillas P Jennings MV , et al. Genome-wide association study of problematic opioid prescription use in 132,113 23andMe research participants of European ancestry. Mol Psychiatry 2021; 26: 6209–6217. 34728798 10.1038/s41380-021-01335-3 PMC8562028

[28] Hatoum AS Colbert SMC Johnson EC , et al. Multivariate genome-wide association meta-analysis of over 1 million subjects identifies loci underlying multiple substance use disorders. Nat Ment Health 2023; 1: 210–223. 37250466 10.1038/s44220-023-00034-y PMC10217792

[29] Malik R Chauhan G Traylor M , et al. Multiancestry genome-wide association study of 520,000 subjects identifies 32 loci associated with stroke and stroke subtypes. Nat Genet 2018; 50: 524–537. 29531354 10.1038/s41588-018-0058-3 PMC5968830

[30] Traylor M Persyn E Tomppo L , et al. Genetic basis of lacunar stroke: a pooled analysis of individual patient data and genome-wide association studies. Lancet Neurol 2021; 20: 351–361. 33773637 10.1016/S1474-4422(21)00031-4 PMC8062914

[31] Woo D Falcone GJ Devan WJ , et al. Meta-analysis of genome-wide association studies identifies 1q22 as a susceptibility locus for intracerebral hemorrhage. Am J Hum Genet 2014; 94: 511–521. 24656865 10.1016/j.ajhg.2014.02.012 PMC3980413

[32] Bowden J Davey Smith G Haycock PC Burgess S. Consistent estimation in Mendelian randomization with some invalid instruments using a weighted median estimator. Genet Epidemiol 2016; 40: 304–314. 27061298 10.1002/gepi.21965 PMC4849733

[33] Ebrahimi H Haghjoo Javanmard S Asgary S Dehghani L Amiri M Saadatnia M. Opium addiction and ischemic stroke in Isfahan, Iran: a case-control study. Eur Neurol 2018; 79: 82–85. 29275418 10.1159/000485098

[34] Hamzei Moqaddam A Ahmadi Musavi SM Khademizadeh K . Relationship of opium dependency and stroke. Addict Health 2009; 1: 6–10. 24494076 PMC3905492

[35] Liu D Yang L Liu P Wang Y Gao L. Impact of cannabis abuse on the occurrence of stroke in young people: a systematic review and meta-analysis. Front Neurol 2024; 15: 1426023. 10.3389/fneur.2024.1426023 PMC11536518 39502388

[36] Storck W Elbaz M Vindis C , et al. Cardiovascular risk associated with the use of cannabis and cannabinoids: a systematic review and meta-analysis. Heart 2025; 111: 1047–1056. 40527600 10.1136/heartjnl-2024-325429

[37] Satsakis A Docea AO Calina D , et al. A mechanistic and pathophysiological approach for stroke associated with drugs of abuse. J Clin Med 2019; 8: 1295. 31450861 10.3390/jcm8091295 PMC6780697

[38] Levy R Schur A Nathan I , et al. Impairment of ADP-induced platelet aggregation by hashish components. Thromb Haemost 1976; 36: 634–640. 1037158

[39] Rendon LF Melta S Leung J , et al. Cocaine and ischemic or hemorrhagic stroke: a systematic review and meta-analysis of clinical evidence. J Clin Med 2023; 12: 5207. 37629248 10.3390/jcm12165207 PMC10455873

[40] Pradhan L Mondal D Chandra S Ali M Agrawal KC. Molecular analysis of cocaine-induced endothelial dysfunction: role of endothelin-1 and nitric oxide. Cardiovasc Toxicol 2008; 8: 161–171. 18813882 10.1007/s12012-008-9025-z

[41] Bachi K Mani V Jeyachandran D , et al. Vascular disease in cocaine addiction. Atherosclerosis 2017; 262: 154–162. 28363516 10.1016/j.atherosclerosis.2017.03.019 PMC5757372

[42] Westover AN McBride S Haley RW. Stroke in young adults who abuse amphetamines or cocaine: a population-based study of hospitalized patients. Arch Gen Psychiatry 2007; 64: 495–502. 17404126 10.1001/archpsyc.64.4.495

[43] Weiss SR Raskind R Morganstern NL Pytlyk PJ Baiz TC. Intracerebral and subarachnoid hemorrhage following use of methamphetamine (“speed”). Int Surg 1970; 53: 123–127. 5416024

[44] Berlit P. Diagnosis and treatment of cerebral vasculitis. Ther Adv Neurol Disord 2010; 3: 29–42. 21180634 10.1177/1756285609347123 PMC3002614

[45] Saberi A , et al. Opium consumption prevalence among patients with ischemic stroke compared with healthy individuals in Iran. Int J High Risk Behav Addict 2017; 6: e27264.

[46] Cai J He L Wang H , et al. Genetic liability for prescription opioid use and risk of cardiovascular diseases: a multivariable Mendelian randomization study. Addiction 2022; 117: 1382–1391. 34859517 10.1111/add.15767

[47] San Luis CV O’Hana S Nobleza C Shekhar S , et al. Association between recent cannabinoid use and acute ischemic stroke. Neurol Clin Pract 2020; 10: 333–339. 32983613 10.1212/CPJ.0000000000000888 PMC7508332

[48] Riley ED Chow FC Josephson SA , et al. Cocaine use and white matter hyperintensities in homeless and unstably housed women. J Stroke Cerebrovasc Dis 2021; 30: 105675. 33677311 10.1016/j.jstrokecerebrovasdis.2021.105675 PMC8415496

[49] Petrie BK Lau H Cajiga-Pena FM , et al. Illicit drug use and cerebral microbleeds in patients with acute ischemic stroke and transient ischemic attack. Int J Stroke 2025; 20: 874–882. 40070091 10.1177/17474930251328524

