# Supplementary material for: Does illicit drug use increase stroke risk? A systematic review, meta-analyses, and Mendelian randomization analysis
Source: Int J Stroke. 2026 Jan 21;21(6):788–800. doi: 10.1177/17474930261418926 (PMC13291408; doi:10.1177/17474930261418926)
Supplement: sj-docx-1-wso-10.1177_17474930261418926 – Supplemental material for Does illicit drug use increase stroke risk? A systematic review, meta-analyses, and Mendelian randomization analysis [file sj-docx-1-wso-10.1177_17474930261418926.docx]

**Supplementary Material**

**Supplementary Appendix**

PRISMA 2020 checklist

Systematic review search strategy

**Supplementary Methods**

Mendelian randomisation data sources

**Supplementary Tables**

Supplementary Table 1: Characteristics of included studies in meta-analysis.

Supplementary Table 2: Newcastle-Ottawa Scale case-control study assessment.

Supplementary Table 3: Newcastle-Ottawa Scale cohort study assessment.

Supplementary Table 4: Newcastle-Ottawa Scale cross-sectional study assessment.

Supplementary Table 5: Publication diagnostics for all-age analysis

Supplementary Table 6: Publication diagnostics restricted to participants under 55 years of age

Supplementary Table 7: All multivariate meta-analysis results.

Supplementary Table 8: All univariate meta-analysis results.

Supplementary Table 9: Meta-regression of effect size on standard error for the all-age analysis.

Supplementary Table 10: Multivariate meta-analysis results for participants under 55 years of age.

Supplementary Table 11: Meta-regression of effect size on standard error for analyses restricted to participants under 55 years of age.

Supplementary Table 12: Sensitivity analysis of studies published after 2010.

Supplementary Table 13. Characteristics of the included genome-wide association studies of substance misuse.

Supplementary Table 14. Mendelian randomisation results of all addiction and stroke subtypes.

**Supplementary Figures**

Supplementary Figure 1. Funnel plots showing publication bias assessment for each drug and stroke type.

Supplementary Figure 2. Funnel plots showing publication bias assessment for each drug for overall stroke, including trim-and-fill adjustments.

Supplementary Figure 3. Forest plot from the univariate random-effects meta-analysis of cannabis and stroke risk.

Supplementary Figure 4. Forest plot from the univariate random-effects meta-analysis of cocaine and stroke risk.

Supplementary Figure 5. Forest plot from the univariate random-effects meta-analysis of amphetamines and stroke risk.

Supplementary Figure 6. Forest plot from the univariate random-effects meta-analysis of opioids and stroke risk.

Supplementary Figure 7. Forest plot from the multivariate random-effects meta-analysis of recent illicit drug use and stroke risk.

Supplementary Figure 8. Mendelian randomisation associations between genetically predicted substance dependence and stroke subtypes.

Supplementary Figure 9. Scatter plots showing genetic associations of substance use exposures and stroke subtypes for significant causal estimates.

Supplementary Figure 10. Leave-one-out analysis for all significant causal estimates identified by Mendelian Randomisation.

**Supplementary Appendix: PRISMA 2020 checklist**

**PRISMA ABSTRACT CHECKLIST**

**
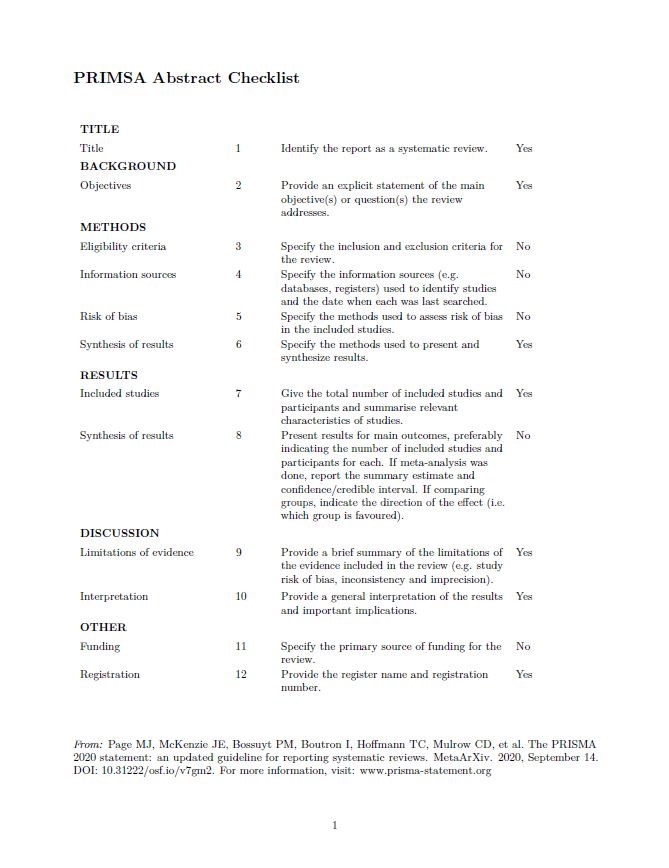
**

**PRISMA 2020 MAIN CHECKLIST**

**
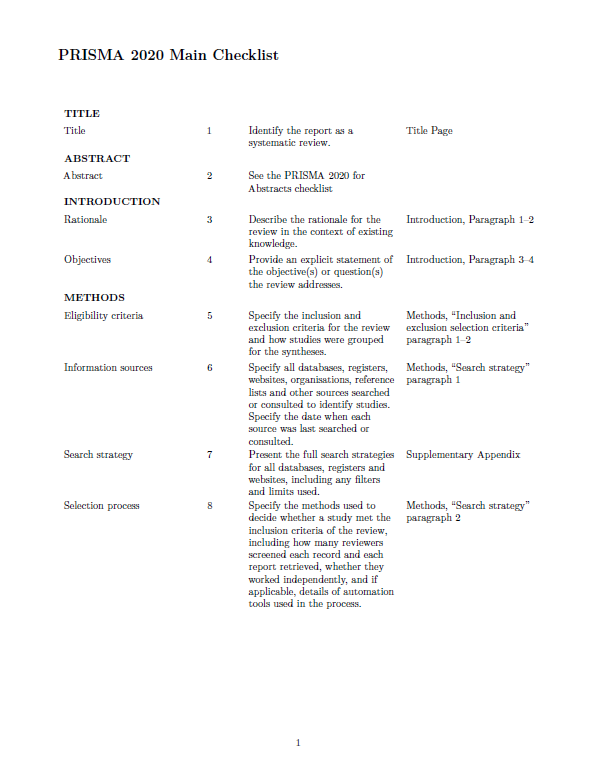
**

**
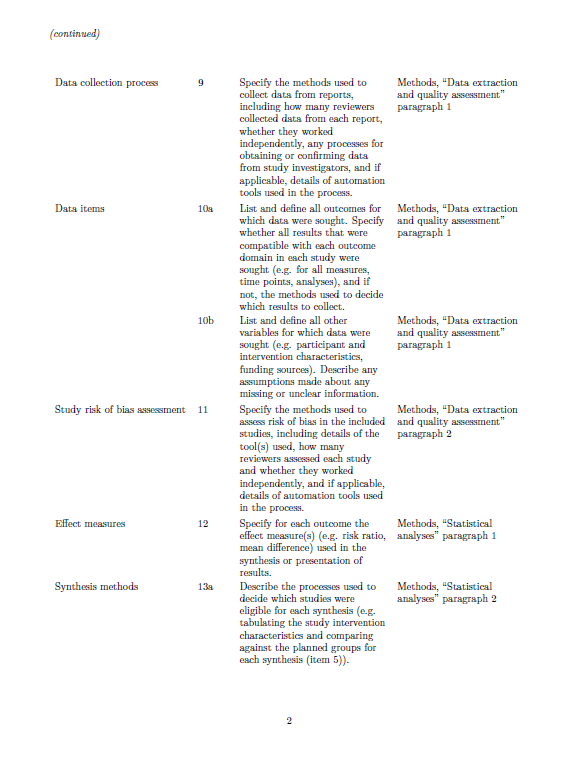
**

**
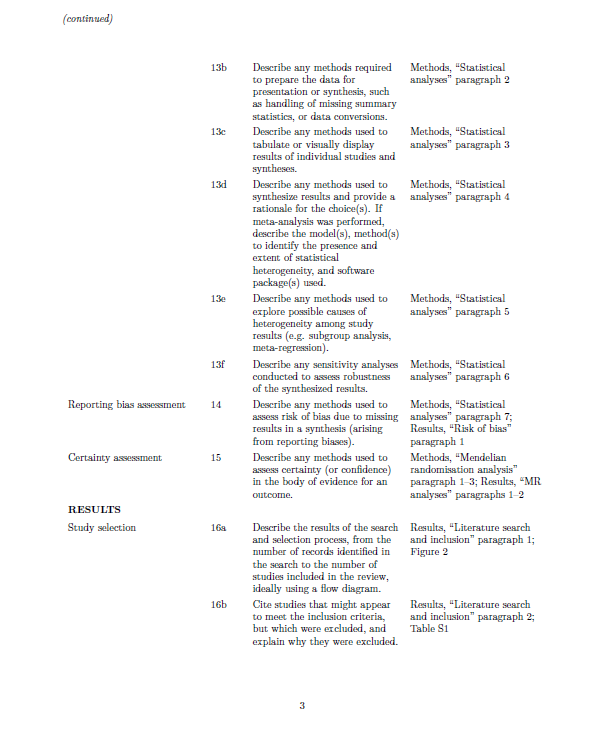
**

**
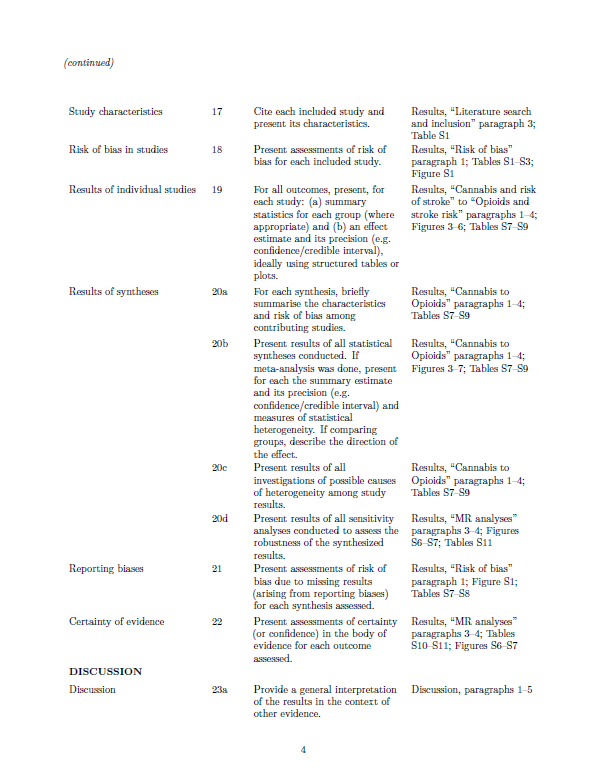
**

**
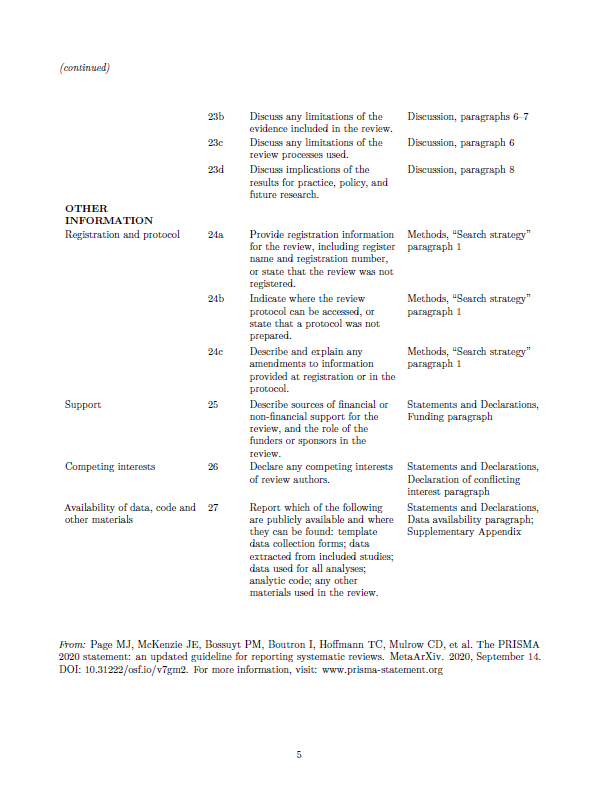
**

**Supplementary Appendix: Systematic review search strategy**

**PubMed**

**#1 Substance use**

("Substance-Related Disorders"[MeSH Terms] OR "Substance Abuse"[tiab] OR "Drug Abuse"[tiab] OR “abuse drugs”[tiab] OR "Drug Misuse"[tiab] OR "Substance Misuse"[tiab] OR "non-medical use"[tiab] OR "illicit use"[tiab] OR misuse[tiab] OR abuse[tiab] OR addicted[tiab] OR addiction[tiab])

**AND**

**#2 Substance type**

(opioid[tiab] OR "analgesics, opioid"[MeSH Terms] OR opiate[tiab] OR "opiate alkaloids"[MeSH Terms] OR heroin[tiab] OR "heroin"[MeSH Terms] OR morphine[tiab] OR "morphine"[MeSH Terms] OR fentanyl[tiab] OR "fentanyl"[MeSH Terms] OR cocaine[tiab] OR "cocaine"[MeSH Terms] OR crack[tiab] OR "crack cocaine"[MeSH Terms] OR “coca leaves”[tiab] OR cannabis[tiab] OR "cannabis"[MeSH Terms] OR marijuana[tiab] OR "cannabis"[MeSH Terms] OR hashish[tiab] OR "cannabis"[MeSH Terms] OR cannabinoid[tiab] OR "cannabinoids"[MeSH Terms] OR amphetamine[tiab] OR "dextroamphetamine"[MeSH Terms] OR "amphetamines"[MeSH Terms] OR "amphetamine"[MeSH Terms] OR amfetamine[tiab] OR "amphetamine"[MeSH Terms] OR benzodiazepine[tiab] OR "benzodiazepines"[MeSH Terms] OR methadone[tiab] OR "methadone"[MeSH Terms] OR prescription[tiab] OR "drug prescriptions"[MeSH Terms] OR phencyclidine[tiab] OR "phencyclidine"[MeSH Terms] OR solvent[tiab] OR "solvents"[MeSH Terms] OR barbiturate[tiab] OR "barbiturates"[MeSH Terms] OR depressant[tiab] OR "hypnotics and sedatives"[MeSH Terms] OR sedative[tiab] OR "hypnotics and sedatives"[MeSH Terms] OR stimulant[tiab] OR "central nervous system stimulants"[MeSH Terms] OR ritalin[tiab] OR "methylphenidate"[MeSH Terms] OR Adderall[tiab] OR methylphenidate[tiab] OR "methylphenidate"[MeSH Terms] OR oxycodone[tiab] OR "oxycodone"[MeSH Terms] OR hydrocodone[tiab] OR "hydrocodone"[MeSH Terms] OR “pain relief”[tiab] OR "pain"[MeSH Terms] OR "pain"[All Fields] OR “pain reliever”[tiab] OR "pain"[MeSH Terms] OR “pain medication”[tiab] OR "pain"[MeSH Terms])

**AND**

**#3 Stroke**

("Stroke"[MeSH Terms] OR stroke[tiab] OR "Ischemic Attack, Transient"[MeSH Terms] OR “ischemic attack”[tiab] OR “ischaemic attack”[tiab] OR “transient ischemic attack”[tiab] OR “cerebrovascular accident”[tiab] OR “intracerebral hemorrhage”[tiab] OR “intracerebral haemorrhage”[tiab] OR "subarachnoid hemorrhage"[tiab] OR "subarachnoid haemorrhage"[tiab])

**AND**

**#4 Study design**

("epidemiology"[tiab] OR "epidemiology"[MeSH Terms]) OR ("systematic review"[tiab] OR "meta-analysis"[tiab] OR "meta analysis"[MeSH Terms]) OR ("cohort study"[tiab]) OR ("cross-sectional study"[tiab] OR "cross sectional study"[tiab]) OR ("prospective study"[tiab]) OR ("retrospective study"[tiab]) OR ("population-based study"[tiab]) OR ("prevalence"[tiab] OR "prevalence"[MeSH Terms]) OR ("risk factors"[tiab] OR "risk factors"[MeSH Terms]) OR ("association"[tiab]) OR ("incidence"[tiab] OR "incidence"[MeSH Terms])

**#5 Combined results**

#1 AND #2 AND #3 AND #4

**Scopus:**

( TITLE-ABS-KEY ( ( "Substance-Related Disorders" OR "Substance Abuse" OR "Drug Abuse" OR "abuse drugs" OR "Drug Misuse" OR "Substance Misuse" OR "non-medical use" OR "illicit use" OR misuse OR abuse OR addicted OR addiction ) ) ) AND ( TITLE-ABS-KEY ( ( opioid OR opiate OR "opiate alkaloids" OR heroin OR morphine OR fentanyl OR cocaine OR crack OR "crack cocaine" OR "coca leaves" OR cannabis OR marijuana OR hashish OR cannabinoid OR amphetamine OR dextroamphetamine OR amphetamines OR amfetamine OR amphetamine OR benzodiazepine OR methadone OR prescription OR "drug prescriptions" OR phencyclidine OR solvent OR barbiturate OR depressant OR "hypnotics and sedatives" OR sedative OR stimulant OR "central nervous system stimulants" OR ritalin OR "methylphenidate" OR adderall OR methylphenidate OR oxycodone OR hydrocodone OR "pain relief" OR pain OR "pain reliever" OR "pain medication" ) ) ) AND ( TITLE-ABS-KEY ( ( stroke OR "ischemic attack" OR "ischaemic attack" OR "transient ischemic attack" OR "cerebrovascular accident" OR "intracerebral hemorrhage" OR "intracerebral haemorrhage" OR "subarachnoid hemorrhage" OR "subarachnoid haemorrhage" ) ) ) AND ( TITLE-ABS-KEY ( ( epidemiology OR "systematic review" OR "meta-analysis" OR "meta analysis" OR "cohort study" OR "cross-sectional study" OR "cross sectional study" OR "prospective study" OR "retrospective study" OR "population-based study" OR prevalence OR "risk factors" OR association OR incidence ) ) )

**Web of Science**

**1:** TS=(("Substance-Related Disorders" OR "Substance Abuse" OR "Drug Abuse" OR “abuse drugs” OR "Drug Misuse" OR "Substance Misuse” OR "non-medical use" OR "illicit use" OR misuse OR abuse OR addicted OR addiction))

**2:** TS=((opioid OR opiate OR "opiate alkaloids" OR heroin OR morphine OR fentanyl OR cocaine OR crack OR "crack cocaine" OR "coca leaves" OR cannabis OR marijuana OR hashish OR cannabinoid OR amphetamine OR dextroamphetamine OR amphetamines OR amfetamine OR amphetamine OR benzodiazepine OR methadone OR prescription OR "drug prescriptions" OR phencyclidine OR solvent OR barbiturate OR depressant OR "hypnotics and sedatives" OR sedative OR stimulant OR "central nervous system stimulants" OR ritalin OR "methylphenidate" OR adderall OR methylphenidate OR oxycodone OR hydrocodone OR "pain relief" OR pain OR "pain reliever" OR "pain medication"))

**3:** TS=((Stroke OR “ischemic attack” OR “ischaemic attack” OR “transient ischemic attack” OR “cerebrovascular accident” OR “intracerebral hemorrhage” OR “intracerebral haemorrhage” OR "subarachnoid hemorrhage" OR "subarachnoid haemorrhage"))

**4:** TS=((epidemiology OR "systematic review" OR "meta-analysis" OR "meta analysis” OR "cohort study" OR "cross-sectional study" OR "cross sectional study" OR "prospective study" OR "retrospective study" OR "population-based study" OR prevalence OR “risk factors" OR association OR incidence))

**5:** #1 AND #2 AND #3 AND #4

**Embase:**

1. drug dependence/

2. substance abuse/

3. drug abuse/

4. drug misuse/

5. addiction/ or substance abuse/

6. 2 or 3 or 4 or 5

7. ("Substance-Related Disorders" or "Substance Abuse" or "Drug Abuse" or "abuse drugs" or "Drug Misuse" or "Substance Misuse" or "non-medical use" or "illicit use" or misuse or abuse or addicted or addiction).ti,ab.

8. opiate/

9. morphine/

10. fentanyl/

11. cocaine/

12. cocaine/

13. cannabis/

14. cannabinoid/

15. amphetamine/

16. dexamphetamine/

17. benzodiazepine/

18. methadone/

19. prescription/

20. phencyclidine/

21. solvent/

22. methylphenidate/

23. oxycodone/

24. hydrocodone/

25. (opioid or opiate or "opiate alkaloids" or heroin or morphine or fentanyl or cocaine or crack or "crack cocaine" or "coca leaves" or cannabis or marijuana or hashish or cannabinoid or amphetamine or dextroamphetamine or amphetamines or amfetamine or amphetamine or benzodiazepine or methadone or prescription or "drug prescriptions" or phencyclidine or solvent or barbiturate or depressant or "hypnotics and sedatives" or sedative or stimulant or "central nervous system stimulants" or ritalin or "methylphenidate" or adderall or methylphenidate or oxycodone or hydrocodone or "pain relief" or pain or "pain reliever" or "pain medication").ti,ab.

26. cerebrovascular accident/

27. transient ischemic attack/

28. brain hemorrhage/

29. (Stroke or "ischemic attack" or "ischaemic attack" or "transient ischemic attack" or "cerebrovascular accident" or "intracerebral hemorrhage" or "intracerebral haemorrhage" or "subarachnoid hemorrhage" or "subarachnoid haemorrhage").ti,ab.

30. epidemiology/

31. "systematic review"/

32. meta analysis/

33. cohort analysis/

34. cross-sectional study/

35. prospective study/

36. retrospective study/

37. prevalence/

38. risk factor/

39. incidence/

40. (epidemiology or "systematic review" or "meta-analysis" or "meta analysis" or "cohort study" or "cross-sectional study" or "cross sectional study" or "prospective study" or "retrospective study" or "population-based study" or prevalence or "risk factors" or "association" or incidence).ti,ab.

41. 1 or 2 or 3 or 4 or 5 or 7

42. 8 or 9 or 10 or 11 or 12 or 13 or 14 or 15 or 16 or 17 or 18 or 19 or 20 or 21 or 22 or 23 or 24 or 25

43. 26 or 27 or 28 or 29

44. 30 or 31 or 32 or 33 or 34 or 35 or 36 or 37 or 38 or 39 or 40

45. 41 and 42 and 43 and 44

**Supplementary Methods: Mendelian randomisation data sources**

***Exposure data***

PAU data was derived from a GWAS of alcohol-related behaviours assessed by questionnaire, including individuals with AUD according to DSM-5 criteria, comprised of 903,147 participants. From this, we identified 53 SNPs associated at a genome-wide significance threshold of *P*<1×10⁻⁸. Similarly, the AUD GWAS was derived from the same study, comprised of only those participants meeting the DSM-5 criteria for a problematic pattern of alcohol use, comprised of 435,563 participants. We identified 44 SNPs at a genome-wide significance threshold of *P*<1×10⁻⁸.^1^

Full summary GWAS statistics for ND were obtained from a Fagerström Test for Nicotine Dependence (FTND)-based GWAS involving 28,677 individuals, identifying 77 SNPs at genome wide significance of *P*<1×10⁻^5^.^2^

CD summary statistics were derived from a GWAS including 2,085 participants meeting the DSM-5 criteria for CD, yielding 14 SNPs associated at *P*<1×10⁻^5^.^3^

Full summary GWAS statistics for CUD were obtained from a GWAS conducted on 42,281 participants with a problematic pattern of cannabis abuse, identifying 13 genome-wide significant SNPs at *P*<1×10⁻⁸.^4^

We obtained data for POU from a GWAS of 132,113 participants assessed via questionnaire regarding using opioids ‘not as prescribed’, identifying 78 SNPs at *P*<1×10⁻⁸.^5^

Finally, summary statistics for the SUD phenotype were obtained from a GWAS designed to identify general addiction genetic risk factor by disaggregating loci shared across PAU, ND, CUD and opioid use disorder (OUD). This study included 1,025,550 participants and identified 27 independent SNPs at a significance threshold of *P*<1×10⁻^7^.^6^

***Outcome data***

Outcome datasets included GWAS summary statistics for five stroke subtypes: AS, AIS, LAS, CES and SVS, which were obtained from the MEGASTROKE consortium^7^ (67,162 cases and 454,450 controls). AIS was comprised of 60,341 cases, of which 9,006 were CES, 6,688 were LAS and 11,710 were SVS. SVS-MRI statistics were derived from a GWAS conducted by Traylor et al 2021, comprised of 6030 cases and 248,929 controls^8^ and GWAS summary statistics on ICH were obtained from the International Stroke Genetics Consortium (ISGC), consisting of 1,545 cases and 1,481 controls.^9^ Stroke cases were defined based on the World Health Organization (WHO) criteria (e.g., sudden onset neurological changes of presumed vascular origin lasting at least 24 hours) and stroke subtypes were classified according to the Trial of Org 10172 in Acute Stroke Treatment (TOAST) criteria.^10^

**Supplementary Table 1:** Characteristics of included studies in meta-analysis

| **First author** | **Year** | **Country** | **Study design** | **Data source** | **Study period** | **Sample size (n)** | **Exposure** | **Timing of exposure relative to the stroke event** | **Outcome** | **Outcome definition** | **Age (yrs)** | **Sex** |
| --- | --- | --- | --- | --- | --- | --- | --- | --- | --- | --- | --- | --- |
| **Barber^11^** | 2013 | New Zealand | Case-control | Hospital records (urine screen) | 2009 - 2012 | 160 cases; 160 matched controls | Cannabis | Detected via urine screen (~72 h after single use; ≤10 weeks daily use) | Ischemic stroke/TIA | Ischemic stroke or TIA confirmed by hospital records; patients with prior stroke/TIA excluded | 18-55 | 62.5% male |
| **Can^12^** | 2018 | USA | Retrospective cohort | Hospital records | 1990-2016 | 4701 patients with 6,411 intracranial aneurysms | Current cocaine use; Current heroin use | Current use at diagnosis of aneurysm rupture vs non-rupture | Aneurysm rupture | Rupture of saccular non-mycotic intracranial aneurysm confirmed by hospital records. | 55.6 ± 13.7 yrs | 22% male |
| **Chelikam^13^** | 2022 | USA | Cross-sectional | Self-reported surveys (NHANES database) | 2013-2018 | Stroke cases: 10,435; Total respondents: 264,740 | Ever use of cannabis; cocaine; opioids; methamphetamines | Self-reported prior to stroke | Stroke | First-reported stroke among adults | 18+ | 47% male |
| **Cheng^14^** | 2016 | USA | Case-control | Self-report via standardized face-to-face interview | 1991‑  2008 | 1090 cases and 1154 controls | Cocaine (frequent users - more than once per week) | More than once per week in the past year prior to stroke onset | Ischemic stroke | First‑ever ischemic stroke | 15-49 | 54% male cases, 47% male controls |
| **Dayyani^15^** | 2019 | Iran | Case-control | Hospital records + patient/next-of-kin interviews | 2015-2017 | 50 cases, 43 controls | Opium consumption | Hazard period — consumption within 4 h prior to rupture | Aneurysmal Rupture | First-ever aneurysmal subarachnoid hemorrhage | 18+ | 56% male cases; 65% male controls |
| **Desai^16^** | 2020 | USA | Retrospective cohort | Hospital records | 2007-2014 | 3,307,310 hospitalisations | Cannabis | Cannabis use was documented at the time of admission | Stroke; Ischemic stroke | Hospital record of ICD-9-CM codes 433.01–434.91, 436 and CCS code 109 | 18-49 | - |
| **Desai^17^** | 2022 | USA | Retrospective cohort | Hospital records | 2015-2017 | 19,448,302 hospitalisations, 623,715 cannabis users | Cannabis | Cannabis use was documented at the time of admission | Stroke | Hospital admission with stroke | 18-44 | 61.2%  Male CUD+; 25.5% CUD- |
| **Desai^18^** | 2024 | USA | Retrospective cohort | Hospital records | 2016-2019 | 1.1 million patients CUD, ~27.4 million hospitalisations | Cannabis (recent) | Cannabis use was documented at the time of admission | Acute ischemic stroke | Hospital admission for AIS (ICD-10 I63) | 18-44 | 57.3% male |
| **Dutta^19^** | 2021 | USA | Population-based case–control | Self-reported via standardized face-to-face interviews (cases verified by hospital records) | 1992-2008 | 751 cases & 813 controls | Moderate use of cannabis (1x/week ≥ amount < 7x/week) | Prior to stroke (self-reported) | Early onset ischemic stroke | First-ever ischemic stroke | 15-49 | 47.3% male (cases), 41.9% male (controls) |
| **Jeffers^20^** | 2024 | USA | Cross-sectional | Self-reported survey | 2016-2020 | 434,104 adults | Cannabis | Cannabis use in the past 30 days | Stroke | Self-reported lifetime history of stroke as told by a health professional | 18-74 | 48.9 (48.6–49.2)% male |
| **Kalla^21^** | 2018 | USA | Retrospective cohort | Hospital records | 2009-2010 | 316,397 cannabis users | Cannabis | Cannabis use documented in hospital records | Cerebrovascular accident; Subarachnoid haemorrhage | Hospital discharge diagnosis | 18-55 | 60% male |
| **Kwok^22^** | 2020 | USA | Retrospective cohort | Hospital records | 2004-2014 | 32,765 CUD; 7,306,012 hospitalizations | Cannabis | Cannabis use documented at hospital admission | Stroke | Hospital record of stroke/TIA | Mean non-cannabis users 64.6 ± 12.3; mean cannabis users 49.5 ± 9.9 | 82.7% male in cannabis users, 66.3% male in non-users |
| **Malhotra^23^** | 2018 | USA | Retrospective cohort | Hospital records | 2004-2011 | 103,356 hospitalized ICH patients (2,306 marijuana users; 93,278 nonusers) | Cannabis; Cocaine; Amphetamines | Current substance use documented during ICH hospitalization | Intracerebral haemorrhage | Hospitalization with *primary diagnosis* of nontraumatic ICH | 15-54 | 66.8% male |
| **Parekh^24^** | 2019 | USA | Cross-sectional | Self-reported data | 2016-2017 | 43,860 participants | Cannabis (frequent users) | Past 30-day marijuana use | Stroke | Self-reported physician diagnosis of stroke | 18-44 | 49.9% male overall; 63.3% among cannabis users |
| **Patel^25^** | 2023 | USA | Retrospective cross-sectional | Administrative hospital discharge records | 2016-2017 | 58,259,589 total hospitalizations; cannabis (n=1,072,879), cocaine (n=341,745) amphetamine (n=295,060) | Cannabis; Cocaine; Amphetamine; Opioids | SUD diagnosis recorded during hospitalization | Acute ischemic stroke; Subarachnoid haemorrhage; Intracerebral haemorrhage | Hospital discharge diagnosis based on ICD-10-CM codes | 18+ | 58% male |
| **Petitti^26^** | 1998 | USA | Case-control | Hospital admission & discharge record; interviews | 1991-1994 | 347 cases, 1021 controls | Any cocaine; Amphetamines | Use in the week before the index date | Stroke | New‑onset stroke; first ever stroke | 15-44 | - |
| **Qureshi^27^** | 1997 | USA | Retrospective case–control | Hospital records | 1990-1994 | 66 stroke cases, 99 controls | Crack-cocaine use (ever) | Recent use defined as within 48 hours of stroke event (acute crack use) | Stroke; Infarction | Clinically diagnosed stroke per WHO criteria | 20-39 | 84% male |
| **Qureshi (a)^28^** | 2001 | USA | Cross-sectional | Self-reported surveys (NHANES database) | 1988-1994 | 10,085 adults | Cocaine, frequent users | Lifetime use | Nonfatal strokes | Self-reported physician diagnosis of stroke | 18-45 | 46.5% male |
| **Qureshi (b)^29^** | 2001 | USA | Case-control | Hospital records | 1990-1997 | 122 ICH; 366 age- and sex-matched controls | Cocaine | Prior or current use (ascertained from hospital and prior medical/pharmacy records) | Intracerebral haemorrhage | Clinically diagnosed stroke | 18+ |  |
| **Rezvani^30^** | 2012 | Iran | Cross-sectional | Hospital records | 2011 | 558 patients | Oral opium addiction | Chronic exposure before diagnosis of stroke | Ischemic stroke | Clinically diagnosed stroke | Mean = 52.6 | 45.0 % male |
| **Rumalla (a)^31^** | 2016 | USA | Retrospective cohort | Hospital records | 2004-2011 | 16,163,453 non-cannabis users & 2,496,165 cannabis users | Cannabis; Cocaine; Amphetamines | Use documented during hospitalisation | Aneurysmal subarachnoid haemorrhage | Primary discharge diagnosis for aSAH | 15-54 | 67.9% male in cannabis user group (AIS subset) vs 52.7% male in non-cannabis group |
| **Rumalla (b)^32^** | 2016 | USA | Retrospective cohort | Hospital records | 2004-2011 | 478,649 AIS hospitalizations;  Cannabis = 11,320, non-cannabis = 467,329 | Cannabis; Cocaine; Opioids; Amphetamines | Recorded in same hospitalisation episode | Acute ischemic stroke | Clinically diagnosed stroke | 15-54 | 67.9 % male cannabis users AIS; 52.7% male non-cannabis users AIS |
| **Saadatnia^33^** | 2024 | Iran | Case-control | Hospital records | 2021-2023 | 806 participants  402 ICH cases, 404 controls | Opioid use disorder | History of opium use prior to intracerebral hemorrhage event in cases | Intracerebral haemorrhage | Clinically and imaging (CT/MRI) confirmed ICH | Mean case group-62.01 ± 14.85 years; Mean control group - 57.86 ± 15.80 years | 59% male ICH cases, 58% male controls |
| **Saberi^34^** | 2016 | Iran | Cross-sectional | Hospital records | 2013-2014 | 83 cases, 83 control | Opium addiction | Exposure assessed prior to or at time of stroke) | Ischemic stroke | Ischemic stroke confirmed by clinical evaluation and CT and/or MRI | Mean case group- 68.55 ± 15.01 years; Mean control group - 67.94 ± 13.55 years | 58% male |
| **San Luis^35^** | 2020 | USA | Retrospective observational cohort | Hospital records | 2015-2017 | 9,350 adults; 1,643 cannabis-positive on urine toxicology | Cannabis (recent) | Measured at first hospital admission | Acute ischemic stroke | First-ever ischemic stroke identified by ICD‑10 codes | 18+ | 51% male cannabis negative; 68% male cannabis positive |
| **Satish^36^** | 2021 | USA | Retrospective cohort | Hospital records | 2017 | 317,688 trauma patients | Cocaine; Amphetamines | Positive urine drug screen at admission | Stroke | Clinically documented in-hospital events | Mean age 41.19-45.79 | 66.8% male |
| **Shah^37^** | 2021 | USA | Cross-sectional | Hospital records | 2016-2018 | 133,706 adults | Frequent marijuana use | Past 30 day use | Stroke | Self-reported physician diagnosis | 17-74 | 57.5% male cannabis users vs 43.2% male non-users |
| **Vaidya^38^** | 2025 | USA | Prospective cohort | Participant interviews, urine toxicology and health records | 2016 – 2019 + 2-year health record follow-up | 238 participants | Opioids (other than heroin and fentanyl) | Baseline exposure assessed at first study visit; outcome assessed during study period and up to 2 years after | Stroke | Documented in clinician notes on health records | 18+ |  |
| **Vin-Raviv^39^** | 2016 | USA | Retrospective cohort | Hospital records | 2007-2011 | 387,608 cannabis users | Cannabis | Exposure recorded at hospitalization | Stroke | Hospital discharge diagnosis of stroke as per ICD‑9 codes | 40+ | 62.2% male cannabis users; 41.5% male non-users |
| **Westover^40^** | 2007 | USA | Retrospective cross-sectional | Hospital records | 2000-2003 | 812,247 hospital discharges | Cannabis; Cocaine; Opioids; Amphetamines | Recorded in hospital discharge diagnoses | Ischemic stroke; hemorrhagic stroke | ICD-9-CM coded discharge diagnosis | 18-44 | - |
| **Winhusen (a)^41^** | 2020 | USA | Retrospective cohort | Electronic health data | 1999-2018 | 8,944 cannabis users and 8,944 matched controls | Cannabis (Regular cannabis use defined as ≥1 encounter diagnosis for cannabis abuse/dependence or ≥2 positive urine drug screens) | Outcomes only counted if initial diagnosis occurred *after* first cannabis-use indication | Cerebrovascular accident | ICD-9/10 diagnoses | 18+ | 56.8% male |
| **Winhusen (b)^42^** | 2020 | USA | Retrospective cohort | Electronic health data | 1999-2018 | 8,244 patients with cocaine addiction and 8,244 matched controls | Cocaine | Outcomes counted only if diagnosis occurred after first cocaine-use indication | Cerebrovascular accident | ICD-9/10 diagnoses | 18+ | 55.9% male |

**Footnote:** *Some studies contributed multiple effect estimates. The meta-analyses included: cannabis (24 estimates from 19 studies), cocaine (18 estimates from 14 studies), amphetamines (11 estimates from 8 studies), and opioids (13 estimates from 10 studies).*

**Supplementary Table 2:** Newcastle- Ottawa Scale case-control study assessment

**Supplementary Table 3:** Newcastle- Ottawa Scale cohort study assessment

**Supplementary Table 4:** Newcastle- Ottawa Scale cross-sectional study assessment

**Supplementary Table 5:** Publication diagnostics for all-age analysis

| Substance | No. effect estimates | Egger p | Begg p | No. imputed (trim-and-fill) | Adjusted OR | Adjusted 95% CI |
| --- | --- | --- | --- | --- | --- | --- |
| Cannabis | 24 | 4.16 × 10⁻⁶ | 0.54 | 5 | 1.12 | 1.02–1.23 |
| Cocaine | 18 | 0.039 | 0.58 | 0 | 1.96† | 1.27–3.01† |
| Opioids | 12 | 0.0929 | 0.60 | 0 | 1.20† | 0.52–2.74† |
| Amphetamines | 11 | 0.040 | 0.54 | 0 | 2.22† | 1.40–3.53† |

**†** Adjusted estimate is identical to the main pooled estimate due to zero trim-and-fill imputations.

**Supplementary Table 6:** Publication diagnostics restricted to participants under 55 years of age

| Substance | No. effect estimates | Egger p | Begg p | No. imputed (trim-and-fill) | Adjusted OR | Adjusted 95% CI |
| --- | --- | --- | --- | --- | --- | --- |
| Cannabis | 13 | 0.0063 | 0.51 | 5 | 1.12 | 1.02–1.23 |
| Cocaine | 12 | 0.214 | 0.84 | 0 | 1.96† | 1.27–3.01† |
| Opioids | 4 | - | - | - | - | - |
| Amphetamines | 7 | - | - | - | - | - |

**†** Adjusted estimate is identical to the main pooled estimate due to zero trim-and-fill imputations.

**Supplementary Table 7:** All multivariate meta-analysis results

| Exposure | Outcome | No. included effect estimates | Analysis | OR | 95% CI | I^2^ | *p-*Value |
| --- | --- | --- | --- | --- | --- | --- | --- |
| Cannabis | **Any stroke** | **24** | **Multivariate** | **1.37** | **1.14-1.65** | **99.0%** | **0.0007** |
|  | Stroke (non-specific) | 10 | Multivariate | 1.16 | 1.03-1.30 | 99.0% | 0.028 |
|  | Ischemic stroke | 8 | Multivariate | 1.39 | 1.23-1.56 | 99.0% | 7.51E-06 |
|  | Haemorrhagic stroke | 7 | Multivariate | 1.11 | 0.97-1.27 | 99.0% | 8.99E-05 |
| Cocaine | **Any stroke** | **18** | **Multivariate** | **1.96** | **1.27-3.01** | **99.5%** | **0.0023** |
|  | Stroke (non-specific) | 6 | Multivariate | 2.02 | 1.17-3.49 | 99.5% | 0.011 |
|  | Ischemic stroke | 5 | Multivariate | 1.38 | 1.21-1.57 | 99.5% | 0.0025 |
|  | Haemorrhagic stroke | 7 | Multivariate | 1.77 | 1.48-2.11 | 99.5% | 0.0002 |
| Opioids | **Any stroke** | **12** | **Multivariate** | **1.20** | **0.52-2.74** | **99.5%** | **0.66** |
|  | Stroke (non-specific) | 2 | Multivariate | - | - | - | - |
|  | Ischemic stroke | 4 | Multivariate | 0.69 | 0.28-1.70 | 99.5% | 0.79 |
|  | Haemorrhagic stroke | 6 | Multivariate | 1.36 | 0.54-3.41 | 99.5% | 0.86 |
| Amphetamines | **Any stroke** | **11** | **Multivariate** | **2.22** | **1.4-3.53** | **99.3%** | **0.0006** |
|  | Stroke (non-specific) | 3 | Multivariate | 1.15 | 0.62-2.16 | 99.3% | 0.65 |
|  | Ischemic stroke | 3 | Multivariate | 2.37 | 1.45-3.86 | 99.3% | 0.0005 |
|  | Haemorrhagic stroke | 5 | Multivariate | 2.83 | 1.40-3.53 | 99.3% | 3.00E-05 |

**Supplementary Table 8:** All univariate meta-analysis results

| Exposure | Outcome | Included studies | Analysis | OR | 95% CI | I^2^ | *p-*Value |
| --- | --- | --- | --- | --- | --- | --- | --- |
| Cannabis | Stroke (non-specific) | 10 | Univariate | 1.23 | 1.12-1.35 | 96.8% | 1.23E-05 |
|  | Ischemic stroke | 8 | Univariate | 1.16 | 1.04-1.29 | 93.7% | 0.0073 |
|  | Haemorrhagic stroke | 6 | Univariate | 1.11 | 0.97-1.26 | 73.7% | 0.13 |
| Cocaine | Stroke (non-specific) | 6 | Univariate | 1.93 | 1.11-3.36 | 80.8% | 0.02 |
|  | Ischemic stroke | 5 | Univariate | 1.38 | 1.21-1.57 | 89.5% | 1.21E-06 |
|  | Haemorrhagic stroke | 6 | Univariate | 1.77 | 1.48-2.11 | 91.9% | 2.02E-10 |
| Opioids | Stroke (non-specific) | 2 | Univariate | - | - | - | - |
|  | Ischemic stroke | 4 | Univariate | 0.41 | 0.36-0.49 | 87.8% | 2.84E-25 |
|  | Haemorrhagic stroke | 4 | Univariate | 1.59 | 0.48-5.29 | 87.7% | 0.45 |
| Amphetamines | Stroke (non-specific) | 3 | Univariate | 1.01 | 0.7-1.48 | 78.2% | 0.94 |
|  | Ischemic stroke | 3 | Univariate | 1.72 | 0.73-4.06 | 99.5% | 0.22 |
|  | Haemorrhagic stroke | 4 | Univariate | 2.77 | 2.01-3.82 | 95.4% | 4.25E-10 |

**Supplementary Table 9:** Meta-regression of effect size on standard error for the all-age analysis

| Substance | Term | Estimate | SE | 95% CI Lower | 95% CI Upper | p-value |
| --- | --- | --- | --- | --- | --- | --- |
| Cannabis | Intercept | 0.128 | 0.062 | 0.007 | 0.249 | 0.038 |
|  | SEI | 1.494 | 0.219 | 1.065 | 1.924 | <0.0001 |
| Cocaine | Intercept | 0.316 | 0.145 | 0.032 | 0.601 | 0.029 |
|  | SEI | 1.422 | 0.516 | 0.411 | 2.433 | 0.0058 |
| Opioids | Intercept | 0.0929 | 0.581 | -1.045 | 1.231 | 0.872 |
|  | SEI | 0.2143 | 0.950 | -1.648 | 2.077 | 0.821 |
| Amphetamines | Intercept | 0.367 | 0.272 | -0.166 | 0.899 | 0.177 |
|  | SEI | 3.241 | 0.873 | 1.530 | 4.951 | <0.001 |

*Abbreviations: SE- standard error, SEI- standard error of the intervention effect.*

**Supplementary Table 10:** Multivariate meta-analysis results for participants under 55 years of age

| Exposure | Outcome | No. included effect estimates | Analysis | OR | 95% CI | I^2^ | *p-*Value |
| --- | --- | --- | --- | --- | --- | --- | --- |
| Cannabis | **All stroke** | **13** | **Multivariate** | **1.14** | **1.05-1.21** | **89.0%** | **0.0014** |
| Cocaine | **All stroke** | **12** | **Multivariate** | **1.97** | **1.03-2.75** | **93.0%** | **0.0001** |
| Opioids | **All stroke** | **4** | **Multivariate** | **0.36** | **0.23-0.56** | **11.0%** | **4.99E-06** |
| Amphetamines | **All stroke** | **7** | **Multivariate** | **2.74** | **1.70-4.40** | **97.0%** | **3.25E-06** |

**Supplementary Table 11:** Meta-regression of effect size on standard error for analyses restricted to participants under 55 years of age

| Substance | Term | Estimate | SE | 95% CI Lower | 95% CI Upper | p-value |
| --- | --- | --- | --- | --- | --- | --- |
| Cannabis | Intercept | 0.012 | 0.049 | -0.083 | 0.109 | 0.792 |
|  | SEI | 2.542 | 0.516 | 1.529 | 3.555 | <0.0001 |
| Cocaine | Intercept | 0.486 | 0.192 | 0.110 | 0.863 | 0.011 |
|  | SEI | 0.955 | 0.686 | -0.390 | 2.301 | 0.164 |
| Opioids | Intercept | -0.823 | 0.021 | -0.865 | -0.780 | 0.883 |
|  | SEI | -1.18 | 0.677 | -2.509 | 0.144 | 0.081 |
| Amphetamines | Intercept | 1.631 | 0.508 | 0.633 | 2.628 | 0.001 |
|  | SEI | -4.139 | 1.344 | 1.501 | 6.775 | 0.002 |

*Abbreviations: SE- standard error, SEI- standard error of the intervention effect.*

**Supplementary Table 12:** Sensitivity analysis of studies published after 2010.

| Drug | Outcome | Main Analysis OR (95% CI) | p-value | Sensitivity Analysis* OR (95% CI) | p-value |
| --- | --- | --- | --- | --- | --- |
| Cannabis | Stroke (non-specific) | 1.16 (1.03-1.30) | 0.028 | 1.16 (1.03-1.30) | 0.028 |
|  | Ischemic | 1.39 (1.23-1.56) | 7.51E-06 | 1.49 (1.25-1.78) | 8.34E-06 |
|  | Haemorrhagic | 1.11 (0.97-1.27) | 8.99E-05 | 1.16 (0.96-1.41) | 0.044 |
|  | **Overall** | **1.37 (1.14-1.65)** | **0.0007** | **1.32 (1.12-1.54)** | **0.0005** |
| Cocaine | Stroke (non-specific) | 2.02 (1.17-3.49) | 0.011 | 1.78 (1.11-2.23) | 0.0034 |
|  | Ischemic | 1.38 (1.21-1.57) | 0.0025 | 1.45 (1.09-1.92) | 0.009 |
|  | Haemorrhagic | 1.77 (1.48-2.11) | 0.0002 | 1.88 (1.41-2.51) | 1.57E-05 |
|  | **Overall** | **1.96 (1.27-3.01)** | **0.0023** | **1.63 (1.27-2.09)** | **0.00017** |
| Opioids | Ischemic | 0.69 (0.28-1.70) | 0.79 | 0.73 (0.36-1.88) | 0.84 |
|  | Haemorrhagic | 1.36 (0.54-3.41) | 0.86 | 1.53 (0.68-3.40) | 0.65 |
|  | **Overall** | **1.20 (0.52-2.74)** | **0.66** | **1.33 (0.68-2.61)** | **0.39** |
| Amphetamines | Stroke (non-specific) | 1.15 (0.62-2.16) | 0.65 | - ** | - ** |
|  | Ischemic | 2.37 (1.45-3.86) | 0.0005 | - ** | - ** |
|  | Haemorrhagic | 2.83 (1.40-3.53) | 3.00E-05 | 2.79 (2.08-3.74) | 5.46E-12 |
|  | **Overall** | **2.22 (1.40-3.53)** | **0.0006** | **1.85 (1.23-2.79)** | **0.0028** |

**Excluding studies published before 2010.*

****** *Sensitivity analysis performed only for outcomes with more than three contributing studies*

**Supplementary Table 13:** Characteristics of the included genome-wide association studies of substance misuse

| Category | Trait abbreviation | Trait description | First Author/Year | Population | Cohort(s) | No. Participants | GWAS Significance cut off | No. SNPs |
| --- | --- | --- | --- | --- | --- | --- | --- | --- |
| Problematic alcohol use^1^ | PAU | Combines alcohol use disorder and alcohol-related problems assessed via questionnaire. | Zhou et al, 2023 | European | MVP; FinnGen; UKB-EUR1; UKB-EUR2; PGC; QIMR AGDS; QIMR TWINS; QIMR GBP; iPSYCH1; iPSYCH2; YP3 | 903,147 | P < 10^-8^ | 53 |
| Alcohol use disorder^1^ | AUD | Problematic pattern of alcohol use – defined by DSM-5 criteria. | Zhou et al, 2023 | European | MVP; PGC; UKB; GSCAN | 435,563 | P < 10^-8^ | 44 |
| Nicotine dependency^2^ | ND | Fagerström Test for Nicotine Dependence (FTND) - questionnaire to quantify addiction to nicotine. | Quach et al, 2020 | European | iNDiGO consortium | 33,791 | P < 10^-5^ | 77 |
| Cocaine dependence^3^ | CD | Diagnoses based on DSM-IV criteria. | Judit Cabana-Domínguez et al, 2019 | European | dbGap | 6,378 | P < 10^-5^ | 14 |
| Cannabis use disorder^4^ | CUD | Problematic pattern of cannabis use. | Levey et al, 2023 | European | MVP; MGB; Yale-Penn3; iPSYCH2; PGC+deCODE | 886,025 | P < 10^-8^ | 13 |
| Problematic opioid use^5^ | POU | Using prescription opioids ‘not as prescribed’ – assessed by questionnaire. | Sanchez-Roige et al, 2021 | European | 23andMe | 132,113 | P < 10^-8^ | 78 |
| Substance abuse disorder^6^ | SUD | Addiction risk factor – including PAU, CUD, tobacco use disorder & opioid use disorder. | Hatoum et al, 2023 | European | MVP; FinnGen; UKB-EUR1; UKB-EUR2; PGC; QIMR AGDS; QIMR TWINS; QIMR GBP; iPSYCH1; iPSYCH2; YP3; 23andMe; ALSPAC; ARIC; BLS; CADD; COGEND; COPDGene; deCODE; EGCUT; FHS; FinnTwin; GERA; GFG; Harvard; HRS; HUNT; MCTFR; MESA; METSIM; NESCOG; NAG-FIN; NTR; SardiNIA; WHI | 1,025, 550 | P < 10^-7^ | 27 |

***Abbreviations:*** *ALSPAC – Avon Longitudinal Study of Parents and Children; ARIC – Atherosclerosis Risk in Communities Study; BLS – Boston Longitudinal Study; CADD – Center for Applied Genomics; COGEND – Collaborative Genetic Study of Nicotine Dependence; COPDGene – Genetic Epidemiology of COPD Study; dbGaP – Database of Genotypes and Phenotypes; deCODE – deCODE Genetics; EGCUT – Estonian Genome Center, University of Tartu; FHS – Framingham Heart Study; FinnGen – Finnish FinnGen Study; FinnTwin – Finnish Twin Cohort; GERA – Genetic Epidemiology Research on Adult Health and Aging; GFG – Genes for Good; GSCAN – GWAS & Sequencing Consortium of Alcohol and Nicotine use; Harvard – Harvard cohorts (e.g., Nurses’ Health Study); HRS – Health and Retirement Study; HUNT – Nord-Trøndelag Health Study; iNDiGO – International Nicotine Dependence GenOmics consortium; iPSYCH1/2 – Lundbeck Foundation Initiative for Integrative Psychiatric Research (phase 1/2); MCTFR – Minnesota Center for Twin and Family Research; MESA – Multi-Ethnic Study of Atherosclerosis; METSIM – Metabolic Syndrome in Men Study; MGB – Mass General Brigham Biobank; MVP – Million Veteran Program; NAG-FIN – Nicotine Addiction Genetics - Finland; NESCOG – Netherlands Study of Cognition, Environment and Genes; NTR – Netherlands Twin Register; PGC – Psychiatric Genomics Consortium; QIMR AGDS – QIMR Australian Genetics of Depression Study; QIMR TWINS – QIMR Twin Studies; QIMR GBP – QIMR Genetics of Bipolar Phenotypes; SardiNIA – SardiNIA Study of Aging; UKB/UKB-EUR1/UKB-EUR2 – UK Biobank European subsets 1 and 2; WHI – Women’s Health Initiative; Yale-Penn3 – Yale-Penn Study (Phase 3); YP3 – Yale-Penn Phase 3; 23andMe – 23andMe Research Team.*

**Supplementary Table 14**: Mendelian randomisation results of all addiction and stroke subtypes

| Exposure | Consortium | Outcome | Method | P value threshold | n SNP | Beta | SE | Z-score | P-value | *q* FDR (IVW) | F-Statistic | R^2^ | MR-Egger Intercept | MR-Egger SE | MR-Egger P-value | MR-PRESSO (Global p) |
| --- | --- | --- | --- | --- | --- | --- | --- | --- | --- | --- | --- | --- | --- | --- | --- | --- |
| Problematic alcohol use | MEGASTROKE | AS | MR Egger | P >108 | 57 | 0.4025123 | 0.255359 | 1.576259863 | 0.12347342 |  | 104.8917 | 0.005506036 | -0.003570877 | 0.004014788 | 0.3776453 | 0.065 |
| Problematic alcohol use | MEGASTROKE | AS | Weighted median | P >108 | 57 | 0.179844 | 0.13797 | 1.303502651 | 0.29535333 |  | 104.8917 | 0.005506036 |  |  |  |  |
| Problematic alcohol use | MEGASTROKE | AS | Inverse variance  weighted | P >108 | 57 | 0.1950056 | 0.103624 | 1.881862933 | 0.05985473 | 0.13966103 | 104.8917 | 0.005506036 |  |  |  |  |
| Problematic alcohol use | MEGASTROKE | AS | Simple mode | P >108 | 57 | 0.647999 | 0.307518 | 2.107188418 | 0.03959264 |  | 104.8917 | 0.005506036 |  |  |  |  |
| Problematic alcohol use | MEGASTROKE | AS | Weighted mode | P >108 | 57 | 0.1758852 | 0.224292 | 0.784178852 | 0.4362397 |  | 104.8917 | 0.005506036 |  |  |  |  |
| Problematic alcohol use | MEGASTROKE | IS | MR Egger | P >108 | 57 | 0.4905212 | 0.278183 | 1.763304012 | 0.07345345 |  | 104.8917 | 0.005506036 | -0.005852475 | 0.004353811 | 0.1843961 | 0.192 |
| Problematic alcohol use | MEGASTROKE | IS | Weighted median | P >108 | 57 | 0.2624571 | 0.151663 | 1.730531634 | 0.08353536 |  | 104.8917 | 0.005506036 |  |  |  |  |
| Problematic alcohol use | MEGASTROKE | IS | Inverse variance  weighted | P >108 | 57 | 0.1477252 | 0.111943 | 1.3196525 | 0.18695093 | 0.32125422 | 104.8917 | 0.005506036 |  |  |  |  |
| Problematic alcohol use | MEGASTROKE | IS | Simple mode | P >108 | 57 | 0.4316353 | 0.284026 | 1.519702408 | 0.13421208 |  | 104.8917 | 0.005506036 |  |  |  |  |
| Problematic alcohol use | MEGASTROKE | IS | Weighted mode | P >108 | 57 | 0.3808615 | 0.215323 | 1.768794822 | 0.08237413 |  | 104.8917 | 0.005506036 |  |  |  |  |
| Problematic alcohol use | MEGASTROKE | CES | MR Egger | P >108 | 57 | 0.8413189 | 0.473017 | 1.778621357 | 0.07345355 |  | 104.8917 | 0.005506036 | -0.007251323 | 0.007361403 | 0.3289149 | 0.697 |
| Problematic alcohol use | MEGASTROKE | CES | Weighted median | P >108 | 57 | 0.3958922 | 0.269663 | 1.468098576 | 0.24203533 |  | 104.8917 | 0.005506036 |  |  |  |  |
| Problematic alcohol use | MEGASTROKE | CES | Inverse variance  weighted | P >108 | 57 | 0.4121737 | 0.184244 | 2.237103054 | **0.01027962** | **0.03597867** | 104.8917 | 0.005506036 |  |  |  |  |
| Problematic alcohol use | MEGASTROKE | CES | Simple mode | P >108 | 57 | -0.07546 | 0.579562 | -0.130202016 | 0.89687317 |  | 104.8917 | 0.005506036 |  |  |  |  |
| Problematic alcohol use | MEGASTROKE | CES | Weighted mode | P >108 | 57 | 0.3083461 | 0.431874 | 0.713972551 | 0.47820956 |  | 104.8917 | 0.005506036 |  |  |  |  |
| Problematic alcohol use | MEGASTROKE | LAS | MR Egger | P >108 | 57 | 0.8147351 | 0.636657 | 1.279707265 | 0.183535565 |  | 104.8917 | 0.005506036 | -0.002040281 | 0.009934149 | 0.8380331 | 0.304 |
| Problematic alcohol use | MEGASTROKE | LAS | Weighted median | P >108 | 57 | 0.3531338 | 0.371329 | 0.951000299 | 0.341604101 |  | 104.8917 | 0.005506036 |  |  |  |  |
| Problematic alcohol use | MEGASTROKE | LAS | Inverse variance  weighted | P >108 | 57 | 0.6946252 | 0.249479 | 2.784308868 | **0.001349633** | **0.0094472** | 104.8917 | 0.005506036 |  |  |  |  |
| Problematic alcohol use | MEGASTROKE | LAS | Simple mode | P >108 | 57 | -0.114754 | 0.825752 | -0.138969519 | 0.998453333 |  | 104.8917 | 0.005506036 |  |  |  |  |
| Problematic alcohol use | MEGASTROKE | LAS | Weighted mode | P >108 | 57 | 0.2179203 | 0.639647 | 0.340688326 | 0.734613058 |  | 104.8917 | 0.005506036 |  |  |  |  |
| Problematic alcohol use | MEGASTROKE | SVS | MR Egger | P >108 | 57 | -0.598449 | 0.62616 | -0.955744748 | 0.343383432 |  | 104.8917 | 0.005506036 | 0.00920452 | 0.009727257 | 0.3481546 | 0.133 |
| Problematic alcohol use | MEGASTROKE | SVS | Weighted median | P >108 | 57 | -0.197269 | 0.339835 | -0.580485259 | 0.556158743 |  | 104.8917 | 0.005506036 |  |  |  |  |
| Problematic alcohol use | MEGASTROKE | SVS | Inverse variance  weighted | P >108 | 57 | -0.052543 | 0.243192 | -0.216057168 | 0.834587355 | 0.8923424 | 104.8917 | 0.005506036 |  |  |  |  |
| Problematic alcohol use | MEGASTROKE | SVS | Simple mode | P >108 | 57 | 0.0660267 | 0.680718 | 0.096995731 | 0.95555333 |  | 104.8917 | 0.005506036 |  |  |  |  |
| Problematic alcohol use | MEGASTROKE | SVS | Weighted mode | P >108 | 57 | -0.164178 | 0.48901 | -0.33573542 | 0.73573855 |  | 104.8917 | 0.005506036 |  |  |  |  |
| Problematic alcohol use | Traylor et al, 2021 | SVS-MRI | MR Egger | P >108 | 53 | -0.42267 | 0.477407 | -0.885345436 | 0.3801257 |  | 105.9586 | 0.005173431 | 0.008265402 | 0.007658636 | 0.2855646 | 0.400 |
| Problematic alcohol use | Traylor et al, 2021 | SVS-MRI | Weighted median | P >108 | 53 | 0.2802045 | 0.299514 | 0.935532021 | 0.6456466 |  | 105.9586 | 0.005173431 |  |  |  |  |
| Problematic alcohol use | Traylor et al, 2021 | SVS-MRI | Inverse variance  weighted | P >108 | 53 | 0.042395 | 0.205804 | 0.205997019 | 0.7367933 | 0.8289432 | 105.9586 | 0.005173431 |  |  |  |  |
| Problematic alcohol use | Traylor et al, 2021 | SVS-MRI | Simple mode | P >108 | 53 | 0.5446221 | 0.615222 | 0.885244982 | 0.3800998 |  | 105.9586 | 0.005173431 |  |  |  |  |
| Problematic alcohol use | Traylor et al, 2021 | SVS-MRI | Weighted mode | P >108 | 53 | 0.217958 | 0.422903 | 0.515384908 | 0.45675757 |  | 105.9586 | 0.005173431 |  |  |  |  |
| Problematic alcohol use | Woo et al, 2014 | ICH | MR Egger | P >108 | 46 | 1.1533787 | 1.980474 | 0.58237496 | 0.5632867 |  | 95.07447 | 0.004033508 | -0.006771332 | 0.02960592 | 0.8201498 | 0.288 |
| Problematic alcohol use | Woo et al, 2014 | ICH | Weighted median | P >108 | 46 | 0.4777109 | 0.90882 | 0.525638862 | 0.5991391 |  | 95.07447 | 0.004033508 |  |  |  |  |
| Problematic alcohol use | Woo et al, 2014 | ICH | Inverse variance  weighted | P >108 | 46 | 0.7222452 | 0.601003 | 1.201732303 | 0.2294673 | 0.32125422 | 95.07447 | 0.004033508 |  |  |  |  |
| Problematic alcohol use | Woo et al, 2014 | ICH | Simple mode | P >108 | 46 | -0.860958 | 1.936613 | -0.444569038 | 0.6355355 |  | 95.07447 | 0.004033508 |  |  |  |  |
| Problematic alcohol use | Woo et al, 2014 | ICH | Weighted mode | P >108 | 46 | -0.180008 | 1.510516 | -0.119169682 | 0.9056713 |  | 95.07447 | 0.004033508 |  |  |  |  |
| Alcohol use disorder | MEGASTROKE | AS | MR Egger | P >107 | 44 | 0.2830569 | 0.32526 | 0.870248912 | 0.38911117 |  | 54.56642 | 0.005482573 | 0.004598724 | 0.004921163 | 0.3539311 | 0.074 |
| Alcohol use disorder | MEGASTROKE | AS | Weighted median | P >108 | 44 | 0.34608 | 0.138144 | 2.505211953 | 0.01223783 |  | 54.56642 | 0.005482573 |  |  |  |  |
| Alcohol use disorder | MEGASTROKE | AS | Inverse variance  weighted | P >108 | 44 | 0.2112306 | 0.102796 | 2.054856327 | **0.01085675** | **0.0379986** | 54.56642 | 0.005482573 |  |  |  |  |
| Alcohol use disorder | MEGASTROKE | AS | Simple mode | P >108 | 44 | 0.4266243 | 0.299304 | 1.425389805 | 0.16126351 |  | 54.56642 | 0.005482573 |  |  |  |  |
| Alcohol use disorder | MEGASTROKE | AS | Weighted mode | P >108 | 44 | 0.4947196 | 0.216775 | 2.28217593 | 0.02533557 |  | 54.56642 | 0.005482573 |  |  |  |  |
| Alcohol use disorder | MEGASTROKE | IS | MR Egger | P >108 | 44 | 0.0736915 | 0.324213 | 0.227293499 | 0.82129833 |  | 54.56642 | 0.005482573 | 0.07369153 | 0.324213 | 0.8212983 | 0.279 |
| Alcohol use disorder | MEGASTROKE | IS | Weighted median | P >108 | 44 | 0.3361695 | 0.145803 | 2.305646329 | 0.01934535 |  | 54.56642 | 0.005482573 |  |  |  |  |
| Alcohol use disorder | MEGASTROKE | IS | Inverse variance  weighted | P >108 | 44 | 0.1941536 | 0.102553 | 1.893198945 | 0.03833133 | 0.0894397 | 54.56642 | 0.005482573 |  |  |  |  |
| Alcohol use disorder | MEGASTROKE | IS | Simple mode | P >108 | 44 | 0.3988032 | 0.293052 | 1.360863807 | 0.18064697 |  | 54.56642 | 0.005482573 |  |  |  |  |
| Alcohol use disorder | MEGASTROKE | IS | Weighted mode | P >108 | 44 | 0.4312497 | 0.23283 | 1.852212413 | 0.05464666 |  | 54.56642 | 0.005482573 |  |  |  |  |
| Alcohol use disorder | MEGASTROKE | CES | MR Egger | P >108 | 44 | 0.2628858 | 0.57316 | 0.458660089 | 0.64884302 |  | 54.56642 | 0.005482573 | 0.003477917 | 0.01037198 | 0.7390541 | 0.903 |
| Alcohol use disorder | MEGASTROKE | CES | Weighted median | P >108 | 44 | 0.3206319 | 0.262649 | 1.220764253 | 0.2221752 |  | 54.56642 | 0.005482573 |  |  |  |  |
| Alcohol use disorder | MEGASTROKE | CES | Inverse variance  weighted | P >108 | 44 | 0.4448241 | 0.184704 | 2.408307887 | **0.0045672** | 0.03197086 | 54.56642 | 0.005482573 |  |  |  |  |
| Alcohol use disorder | MEGASTROKE | CES | Simple mode | P >108 | 44 | 0.2862272 | 0.521278 | 0.549087014 | 0.56544566 |  | 54.56642 | 0.005482573 |  |  |  |  |
| Alcohol use disorder | MEGASTROKE | CES | Weighted mode | P >108 | 44 | 0.2086142 | 0.41038 | 0.508343602 | 0.64535355 |  | 54.56642 | 0.005482573 |  |  |  |  |
| Alcohol use disorder | MEGASTROKE | LAS | MR Egger | P >108 | 44 | 0.2509191 | 0.786535 | 0.319018282 | 0.7512939 |  | 54.56642 | 0.005482573 | 0.002670436 | 0.01419659 | 0.851701 | 0.318 |
| Alcohol use disorder | MEGASTROKE | LAS | Weighted median | P >108 | 44 | 0.2613559 | 0.38832 | 0.673042594 | 0.33875666 |  | 54.56642 | 0.005482573 |  |  |  |  |
| Alcohol use disorder | MEGASTROKE | LAS | Inverse variance  weighted | P >108 | 44 | 0.3911218 | 0.248357 | 1.574840119 | 0.1152934 | 0.16141076 | 54.56642 | 0.005482573 |  |  |  |  |
| Alcohol use disorder | MEGASTROKE | LAS | Simple mode | P >108 | 44 | -0.099229 | 0.864627 | -0.114765444 | 0.9091653 |  | 54.56642 | 0.005482573 |  |  |  |  |
| Alcohol use disorder | MEGASTROKE | LAS | Weighted mode | P >108 | 44 | 0.1591221 | 0.672957 | 0.236452341 | 0.8142045 |  | 54.56642 | 0.005482573 |  |  |  |  |
| Alcohol use disorder | MEGASTROKE | SVS | MR Egger | P >108 | 44 | -0.605936 | 0.757167 | -0.800267882 | 0.4234244 |  | 54.56642 | 0.005482573 | 0.01422553 | 0.0136441 | 0.3030916 | 0.399 |
| Alcohol use disorder | MEGASTROKE | SVS | Weighted median | P >108 | 44 | -0.154551 | 0.356628 | -0.433366945 | 0.8456465 |  | 54.56642 | 0.005482573 |  |  |  |  |
| Alcohol use disorder | MEGASTROKE | SVS | Inverse variance  weighted | P >108 | 44 | 0.1425438 | 0.240947 | 0.591597304 | 0.3541203 | 0.41314035 | 54.56642 | 0.005482573 |  |  |  |  |
| Alcohol use disorder | MEGASTROKE | SVS | Simple mode | P >108 | 44 | 0.0036988 | 0.729767 | 0.005068499 | 0.9959794 |  | 54.56642 | 0.005482573 |  |  |  |  |
| Alcohol use disorder | MEGASTROKE | SVS | Weighted mode | P >108 | 44 | -0.15823 | 0.556386 | -0.284389321 | 0.7774771 |  | 54.56642 | 0.005482573 |  |  |  |  |
| Alcohol use disorder | Traylor et al, 2021 | SVS-MRI | MR Egger | P >108 | 42 | -0.562309 | 0.684502 | -0.821486542 | 0.5385735 |  | 53.62455 | 0.005144756 | 0.01082657 | 0.01216378 | 0.3787525 | 0.931 |
| Alcohol use disorder | Traylor et al, 2021 | SVS-MRI | Weighted median | P >108 | 42 | 0.3663516 | 0.3005 | 1.219138983 | 0.2227914 |  | 53.62455 | 0.005144756 |  |  |  |  |
| Alcohol use disorder | Traylor et al, 2021 | SVS-MRI | Inverse variance  weighted | P >108 | 42 | 0.0185134 | 0.206657 | 0.089584964 | 0.7286171 | 0.7286171 | 53.62455 | 0.005144756 |  |  |  |  |
| Alcohol use disorder | Traylor et al, 2021 | SVS-MRI | Simple mode | P >108 | 42 | 0.5004369 | 0.655161 | 0.763838297 | 0.63453535 |  | 53.62455 | 0.005144756 |  |  |  |  |
| Alcohol use disorder | Traylor et al, 2021 | LAS | Weighted mode | P >108 | 42 | 0.4269169 | 0.550389 | 0.77566373 | 0.4423989 |  | 53.62455 | 0.005144756 |  |  |  |  |
| Alcohol use disorder | Woo et al, 2014 | ICH | MR Egger | P >108 | 35 | 1.2528267 | 2.000852 | 0.626146518 | 0.5355262 |  | 53.86655 | 0.004310188 | -0.005093456 | 0.03545175 | 0.8866325 | 0.145 |
| Alcohol use disorder | Woo et al, 2014 | ICH | Weighted median | P >108 | 35 | 0.4290801 | 0.87613 | 0.489744897 | 0.7465466 |  | 53.86655 | 0.004310188 |  |  |  |  |
| Alcohol use disorder | Woo et al, 2014 | ICH | Inverse variance  weighted | P >108 | 35 | 0.9798696 | 0.618569 | 1.58408995 | 0.1131733 | 0.16141076 | 53.86655 | 0.004310188 |  |  |  |  |
| Alcohol use disorder | Woo et al, 2014 | ICH | Simple mode | P >108 | 35 | -0.730344 | 1.866203 | -0.391352636 | 0.6979785 |  | 53.86655 | 0.004310188 |  |  |  |  |
| Alcohol use disorder | Woo et al, 2014 | ICH | Weighted mode | P >108 | 35 | -0.060845 | 1.347502 | -0.0451536 | 0.964249 |  | 53.86655 | 0.004310188 |  |  |  |  |
| Nicotine dependency | MEGASTROKE | AS | MR Egger | P > 105 | 77 | -0.021246 | 0.074324 | -0.285859365 | 0.7353559 |  | 99.76683 | 0.1855789 | 0.002999293 | 0.004251874 | 0.4827435 | 0.412 |
| Nicotine dependency | MEGASTROKE | AS | Weighted median | P > 105 | 77 | 0.0137058 | 0.043107 | 0.317952387 | 0.6436535 |  | 99.76683 | 0.1855789 |  |  |  |  |
| Nicotine dependency | MEGASTROKE | AS | Inverse variance  weighted | P > 105 | 77 | 0.0262458 | 0.03138 | 0.836380526 | 0.703543 | 0.85699133 | 99.76683 | 0.1855789 |  |  |  |  |
| Nicotine dependency | MEGASTROKE | AS | Simple mode | P > 105 | 77 | -0.057477 | 0.110793 | -0.518777605 | 0.6054232 |  | 99.76683 | 0.1855789 |  |  |  |  |
| Nicotine dependency | MEGASTROKE | AS | Weighted mode | P > 105 | 77 | 0.0306729 | 0.097711 | 0.313913913 | 0.6353355 |  | 99.76683 | 0.1855789 |  |  |  |  |
| Nicotine dependency | MEGASTROKE | IS | MR Egger | P > 105 | 77 | 0.0199473 | 0.084083 | 0.237232952 | 0.8242435 |  | 99.76683 | 0.1855789 | 0.000587678 | 0.004795662 | 0.9027963 | 0.243 |
| Nicotine dependency | MEGASTROKE | IS | Weighted median | P > 105 | 77 | 0.0394149 | 0.048992 | 0.804522506 | 0.4210953 |  | 99.76683 | 0.1855789 |  |  |  |  |
| Nicotine dependency | MEGASTROKE | IS | Inverse variance  weighted | P > 105 | 77 | 0.0292797 | 0.035408 | 0.826913406 | 0.725345 | 0.85699133 | 99.76683 | 0.1855789 |  |  |  |  |
| Nicotine dependency | MEGASTROKE | IS | Simple mode | P > 105 | 77 | 0.0097644 | 0.123788 | 0.078879834 | 0.9373355 |  | 99.76683 | 0.1855789 |  |  |  |  |
| Nicotine dependency | MEGASTROKE | IS | Weighted mode | P > 105 | 77 | 0.020373 | 0.097935 | 0.208026058 | 0.8357651 |  | 99.76683 | 0.1855789 |  |  |  |  |
| Nicotine dependency | MEGASTROKE | CES | MR Egger | P > 105 | 77 | 0.1098386 | 0.169108 | 0.649518653 | 0.63534367 |  | 99.76683 | 0.1855789 | -0.004535518 | 0.009537398 | 0.6357584 | 0.206 |
| Nicotine dependency | MEGASTROKE | CES | Weighted median | P > 105 | 77 | -0.012244 | 0.100134 | -0.12228053 | 0.9026768 |  | 99.76683 | 0.1855789 |  |  |  |  |
| Nicotine dependency | MEGASTROKE | CES | Inverse variance  weighted | P > 105 | 77 | 0.0368972 | 0.070853 | 0.520758757 | 0.2565467 | 0.798546 | 99.76683 | 0.1855789 |  |  |  |  |
| Nicotine dependency | MEGASTROKE | CES | Simple mode | P > 105 | 77 | -0.029551 | 0.215723 | -0.136987236 | 0.8913984 |  | 99.76683 | 0.1855789 |  |  |  |  |
| Nicotine dependency | MEGASTROKE | CES | Weighted mode | P > 105 | 77 | -0.081636 | 0.190346 | -0.428879897 | 0.6664645 |  | 99.76683 | 0.1855789 |  |  |  |  |
| Nicotine dependency | MEGASTROKE | LAS | MR Egger | P > 105 | 77 | 0.0447048 | 0.211945 | 0.210925977 | 0.8335171 |  | 99.76683 | 0.1855789 | 0.003146335 | 0.01196932 | 0.7933741 | 0.340 |
| Nicotine dependency | MEGASTROKE | LAS | Weighted median | P > 105 | 77 | 0.1612197 | 0.123888 | 1.301336912 | 0.2453975 |  | 99.76683 | 0.1855789 |  |  |  |  |
| Nicotine dependency | MEGASTROKE | LAS | Inverse variance  weighted | P > 105 | 77 | 0.0953182 | 0.088042 | 1.082639596 | 0.7345643 | 0.85699133 | 99.76683 | 0.1855789 |  |  |  |  |
| Nicotine dependency | MEGASTROKE | LAS | Simple mode | P > 105 | 77 | 0.2055456 | 0.309381 | 0.664376204 | 0.5084603 |  | 99.76683 | 0.1855789 |  |  |  |  |
| Nicotine dependency | MEGASTROKE | LAS | Weighted mode | P > 105 | 77 | 0.1845152 | 0.271991 | 0.678387028 | 0.4995868 |  | 99.76683 | 0.1855789 |  |  |  |  |
| Nicotine dependency | MEGASTROKE | SVS | MR Egger | P > 105 | 77 | 0.3391397 | 0.184992 | 1.833271637 | 0.0564646 |  | 99.76683 | 0.1855789 | -0.01634304 | 0.01062295 | 0.1281432 | 0.062 |
| Nicotine dependency | MEGASTROKE | SVS | Weighted median | P > 105 | 77 | 0.0746843 | 0.116102 | 0.643267159 | 0.52005075 |  | 99.76683 | 0.1855789 |  |  |  |  |
| Nicotine dependency | MEGASTROKE | SVS | Inverse variance  weighted | P > 105 | 77 | 0.0819483 | 0.079923 | 1.025347045 | 0.3422343 | 0.798546 | 99.76683 | 0.1855789 |  |  |  |  |
| Nicotine dependency | MEGASTROKE | SVS | Simple mode | P > 105 | 77 | 0.0233497 | 0.286391 | 0.081530878 | 0.93523411 |  | 99.76683 | 0.1855789 |  |  |  |  |
| Nicotine dependency | MEGASTROKE | SVS | Weighted mode | P > 105 | 77 | 0.0114793 | 0.257466 | 0.0445857 | 0.96455447 |  | 99.76683 | 0.1855789 |  |  |  |  |
| Nicotine dependency | Traylor et al, 2021 | SVS-MRI | MR Egger | P > 105 | 53 | 0.446512 | 0.231356 | 1.929979257 | 0.05918014 |  | 51.73345 | 0.07516331 | -0.02451637 | 0.01230336 | 0.05166384 | 0.189 |
| Nicotine dependency | Traylor et al, 2021 | SVS-MRI | Weighted median | P > 105 | 53 | 0.2148498 | 0.112826 | 1.904252217 | 0.06456463 |  | 51.73345 | 0.07516331 |  |  |  |  |
| Nicotine dependency | Traylor et al, 2021 | SVS-MRI | Inverse variance  weighted | P > 105 | 53 | 0.0157509 | 0.084749 | 0.185854066 | 0.99574343 | 0.9957445 | 51.73345 | 0.07516331 |  |  |  |  |
| Nicotine dependency | Traylor et al, 2021 | SVS-MRI | Simple mode | P > 105 | 53 | 0.3054784 | 0.301655 | 1.012675283 | 0.31590427 |  | 51.73345 | 0.07516331 |  |  |  |  |
| Nicotine dependency | Traylor et al, 2021 | SVS-MRI | Weighted mode | P > 105 | 53 | 0.3054784 | 0.285931 | 1.06836215 | 0.37365473 |  | 51.73345 | 0.07516331 |  |  |  |  |
| Nicotine dependency | Woo et al, 2014 | ICH | MR Egger | P > 105 | 25 | -1.441455 | 0.909107 | -1.585571537 | 0.1264908 |  | 41.84808 | 0.0300536 | 0.08393134 | 0.04526048 | 0.0765478 | 0.441 |
| Nicotine dependency | Woo et al, 2014 | ICH | Weighted median | P > 105 | 25 | 0.0847027 | 0.411712 | 0.205732794 | 0.6485674 |  | 41.84808 | 0.0300536 |  |  |  |  |
| Nicotine dependency | Woo et al, 2014 | ICH | Inverse variance  weighted | P > 105 | 25 | 0.166764 | 0.274372 | 0.607802806 | 0.3346345 | 0.798546 | 41.84808 | 0.0300536 |  |  |  |  |
| Nicotine dependency | Woo et al, 2014 | ICH | Simple mode | P > 105 | 25 | 0.2257855 | 0.851702 | 0.265099205 | 0.7931967 |  | 41.84808 | 0.0300536 |  |  |  |  |
| Nicotine dependency | Woo et al, 2014 | ICH | Weighted mode | P > 105 | 25 | 0.2423082 | 0.876528 | 0.276440824 | 0.8845762 |  | 41.84808 | 0.0300536 |  |  |  |  |
| Cocaine dependency | MEGASTROKE | AS | MR Egger | P > 105 | 14 | -0.731984 | 0.030991 | -23.61958843 | 0.8266225 |  | 186.2676 | 0.2906944 | 0.0084296 | 0.008873723 | 0.3608749 | 0.073 |
| Cocaine dependency | MEGASTROKE | AS | Weighted median | P > 105 | 14 | 0.0168401 | 0.016245 | 1.036626854 | 0.2999099 |  | 186.2676 | 0.2906944 |  |  |  |  |
| Cocaine dependency | MEGASTROKE | AS | Inverse variance  weighted | P > 105 | 14 | 0.020026 | 0.012439 | 1.609882688 | 0.1074233 | 0.2506543 | 186.2676 | 0.2906944 |  |  |  |  |
| Cocaine dependency | MEGASTROKE | AS | Simple mode | P > 105 | 14 | 0.0175819 | 0.024356 | 0.721866769 | 0.6538575 |  | 186.2676 | 0.2906944 |  |  |  |  |
| Cocaine dependency | MEGASTROKE | AS | Weighted mode | P > 105 | 14 | 0.0172196 | 0.027523 | 0.625648117 | 0.5564666 |  | 186.2676 | 0.2906944 |  |  |  |  |
| Cocaine dependency | MEGASTROKE | IS | MR Egger | P > 105 | 14 | -0.003268 | 0.034207 | -0.095524723 | 0.9254747 |  | 186.2676 | 0.2906944 | 0.006660495 | 0.009788606 | 0.5091458 | 0.452 |
| Cocaine dependency | MEGASTROKE | IS | Weighted median | P > 105 | 14 | 0.0136333 | 0.017857 | 0.763463537 | 0.445187 |  | 186.2676 | 0.2906944 |  |  |  |  |
| Cocaine dependency | MEGASTROKE | IS | Inverse variance  weighted | P > 105 | 14 | 0.01805 | 0.013734 | 1.314306466 | 0.1887431 | 0.33030042 | 186.2676 | 0.2906944 |  |  |  |  |
| Cocaine dependency | MEGASTROKE | IS | Simple mode | P > 105 | 14 | 0.0076484 | 0.030098 | 0.254119664 | 0.8033782 |  | 186.2676 | 0.2906944 |  |  |  |  |
| Cocaine dependency | MEGASTROKE | IS | Weighted mode | P > 105 | 14 | 0.0062637 | 0.028388 | 0.220650233 | 0.8287923 |  | 186.2676 | 0.2906944 |  |  |  |  |
| Cocaine dependency | MEGASTROKE | CES | MR Egger | P > 105 | 14 | 0.0803108 | 0.070201 | 1.144007753 | 0.27492821 |  | 186.2676 | 0.2906944 | -0.001091875 | 0.01971208 | 0.9567384 | 0.066 |
| Cocaine dependency | MEGASTROKE | CES | Weighted median | P > 105 | 14 | 0.0843586 | 0.039468 | 2.137410661 | 0.02564777 |  | 186.2676 | 0.2906944 |  |  |  |  |
| Cocaine dependency | MEGASTROKE | CES | Inverse variance  weighted | P > 105 | 14 | 0.076725 | 0.027157 | 2.825242654 | **0.0011448** | **0.0040068** | 186.2676 | 0.2906944 |  |  |  |  |
| Cocaine dependency | MEGASTROKE | CES | Simple mode | P > 105 | 14 | 0.1444773 | 0.076603 | 1.886065106 | 0.08182548 |  | 186.2676 | 0.2906944 |  |  |  |  |
| Cocaine dependency | MEGASTROKE | CES | Weighted mode | P > 105 | 14 | -0.013072 | 0.071044 | -0.183996052 | 0.93756355 |  | 186.2676 | 0.2906944 |  |  |  |  |
| Cocaine dependency | MEGASTROKE | LAS | MR Egger | P > 105 | 14 | -0.109651 | 0.103156 | -1.062958436 | 0.3087243 |  | 186.2676 | 0.2906944 | 0.03567974 | 0.02921211 | 0.2453873 | 0.423 |
| Cocaine dependency | MEGASTROKE | LAS | Weighted median | P > 105 | 14 | 0.0676226 | 0.046773 | 1.445755857 | 0.23784567 |  | 186.2676 | 0.2906944 |  |  |  |  |
| Cocaine dependency | MEGASTROKE | LAS | Inverse variance  weighted | P > 105 | 14 | 0.0061371 | 0.041436 | 0.148111398 | 0.8238743 | 0.9247622 | 186.2676 | 0.2906944 |  |  |  |  |
| Cocaine dependency | MEGASTROKE | LAS | Simple mode | P > 105 | 14 | 0.1005114 | 0.098306 | 1.022430206 | 0.3252279 |  | 186.2676 | 0.2906944 |  |  |  |  |
| Cocaine dependency | MEGASTROKE | LAS | Weighted mode | P > 105 | 14 | 0.0926782 | 0.09016 | 1.027936151 | 0.322727 |  | 186.2676 | 0.2906944 |  |  |  |  |
| Cocaine dependency | MEGASTROKE | SVS | MR Egger | P > 105 | 14 | 0.0197763 | 0.083705 | 0.236262189 | 0.8172138 |  | 186.2676 | 0.2906944 | -0.001889493 | 0.02369031 | 0.9377444 | 0.111 |
| Cocaine dependency | MEGASTROKE | SVS | Weighted median | P > 105 | 14 | 0.0263797 | 0.043447 | 0.607169055 | 0.5437388 |  | 186.2676 | 0.2906944 |  |  |  |  |
| Cocaine dependency | MEGASTROKE | SVS | Inverse variance  weighted | P > 105 | 14 | 0.0136474 | 0.031942 | 0.427254177 | 0.6691942 | 0.7807265 | 186.2676 | 0.2906944 |  |  |  |  |
| Cocaine dependency | MEGASTROKE | SVS | Simple mode | P > 105 | 14 | 0.0209478 | 0.075041 | 0.279152975 | 0.784519 |  | 186.2676 | 0.2906944 |  |  |  |  |
| Cocaine dependency | MEGASTROKE | SVS | Weighted mode | P > 105 | 14 | 0.011424 | 0.073829 | 0.154735671 | 0.8794062 |  | 186.2676 | 0.2906944 |  |  |  |  |
| Cocaine dependency | Traylor et al, 2021 | SVS-MRI | MR Egger | P > 105 | 12 | 0.0165609 | 0.06953 | 0.238182271 | 0.8165493 |  | 183.0262 | 0.2565393 | -0.01735991 | 0.02119827 | 0.4319092 | 0.538 |
| Cocaine dependency | Traylor et al, 2021 | SVS-MRI | Weighted median | P > 105 | 12 | -0.047253 | 0.041809 | -1.130222096 | 0.2583826 |  | 183.0262 | 0.2565393 |  |  |  |  |
| Cocaine dependency | Traylor et al, 2021 | SVS-MRI | Inverse variance  weighted | P > 105 | 12 | -0.034983 | 0.029545 | -1.18404794 | 0.2363941 | 0.33095174 | 183.0262 | 0.2565393 |  |  |  |  |
| Cocaine dependency | Traylor et al, 2021 | SVS-MRI | Simple mode | P > 105 | 12 | -0.048138 | 0.069951 | -0.688161812 | 0.5242442 |  | 183.0262 | 0.2565393 |  |  |  |  |
| Cocaine dependency | Traylor et al, 2021 | SVS-MRI | Weighted mode | P > 105 | 12 | -0.046941 | 0.066445 | -0.706467901 | 0.4945896 |  | 183.0262 | 0.2565393 |  |  |  |  |
| Cocaine dependency | Woo et al, 2014 | ICH | MR Egger | P > 105 | 8 | 0.3136367 | 0.24779 | 1.265734322 | 0.252534789 |  | 156.2701 | 0.1640812 | 0.002154778 | 0.06906103 | 0.9761212 | 0.986 |
| Cocaine dependency | Woo et al, 2014 | ICH | Weighted median | P > 105 | 8 | 0.3189953 | 0.117323 | 2.71894731 | 0.006548999 |  | 156.2701 | 0.1640812 |  |  |  |  |
| Cocaine dependency | Woo et al, 2014 | ICH | Inverse variance  weighted | P > 105 | 8 | 0.3208235 | 0.091344 | 3.512257021 | **8.66E-05** | **0.00060599** | 156.2701 | 0.1640812 |  |  |  |  |
| Cocaine dependency | Woo et al, 2014 | ICH | Simple mode | P > 105 | 8 | 0.3374505 | 0.16437 | 2.052998243 | 0.079179206 |  | 156.2701 | 0.1640812 |  |  |  |  |
| Cocaine dependency | Woo et al, 2014 | ICH | Weighted mode | P > 105 | 8 | 0.3237331 | 0.162726 | 1.989440512 | 0.086962997 |  | 156.2701 | 0.1640812 |  |  |  |  |
| Cannabis use disorder | MEGASTROKE | AS | MR Egger | P >108 | 13 | -0.218737 | 0.182153 | -1.20084026 | 0.43644646 |  | 1336.15 | 0.02067621 | 0.02119151 | 0.01149668 | 0.0923759 | 0.679 |
| Cannabis use disorder | MEGASTROKE | AS | Weighted median | P >108 | 13 | 0.1027555 | 0.066633 | 1.542106938 | 0.1230476 |  | 1336.15 | 0.02067621 |  |  |  |  |
| Cannabis use disorder | MEGASTROKE | AS | Inverse variance  weighted | P >108 | 13 | 0.1055607 | 0.047185 | 2.237182018 | **0.00225274** | **0.01576921** | 1336.15 | 0.02067621 |  |  |  |  |
| Cannabis use disorder | MEGASTROKE | AS | Simple mode | P >108 | 13 | 0.1141469 | 0.120396 | 0.948093951 | 0.36178446 |  | 1336.15 | 0.02067621 |  |  |  |  |
| Cannabis use disorder | MEGASTROKE | AS | Weighted mode | P >108 | 13 | 0.0764323 | 0.100634 | 0.759511666 | 0.58357355 |  | 1336.15 | 0.02067621 |  |  |  |  |
| Cannabis use disorder | MEGASTROKE | IS | MR Egger | P >108 | 13 | -0.194009 | 0.19679 | -0.985865198 | 0.3453879 |  | 1336.15 | 0.02067621 | 0.01882329 | 0.01238689 | 0.156816 | 0.548 |
| Cannabis use disorder | MEGASTROKE | IS | Weighted median | P >108 | 13 | 0.1009439 | 0.067196 | 1.502236525 | 0.28374655 |  | 1336.15 | 0.02067621 |  |  |  |  |
| Cannabis use disorder | MEGASTROKE | IS | Inverse variance  weighted | P >108 | 13 | 0.0948391 | 0.050952 | 1.861355902 | 0.0326939 | 0.07628576 | 1336.15 | 0.02067621 |  |  |  |  |
| Cannabis use disorder | MEGASTROKE | IS | Simple mode | P >108 | 13 | 0.1234184 | 0.121701 | 1.014115386 | 0.34734653 |  | 1336.15 | 0.02067621 |  |  |  |  |
| Cannabis use disorder | MEGASTROKE | IS | Weighted mode | P >108 | 13 | 0.1234184 | 0.120909 | 1.020752429 | 0.4675103 |  | 1336.15 | 0.02067621 |  |  |  |  |
| Cannabis use disorder | MEGASTROKE | CES | MR Egger | P >108 | 13 | -0.267313 | 0.390809 | -0.683997883 | 0.5081299 |  | 1336.15 | 0.02067621 | 0.02457108 | 0.02442779 | 0.3360942 | 0.503 |
| Cannabis use disorder | MEGASTROKE | CES | Weighted median | P >108 | 13 | 0.0836293 | 0.136279 | 0.613661884 | 0.5394388 |  | 1336.15 | 0.02067621 |  |  |  |  |
| Cannabis use disorder | MEGASTROKE | CES | Inverse variance  weighted | P >108 | 13 | 0.1131772 | 0.098198 | 1.1525371 | 0.1491004 | 0.2609257 | 1336.15 | 0.02067621 |  |  |  |  |
| Cannabis use disorder | MEGASTROKE | CES | Simple mode | P >108 | 13 | 0.0559568 | 0.23627 | 0.23683359 | 0.8167805 |  | 1336.15 | 0.02067621 |  |  |  |  |
| Cannabis use disorder | MEGASTROKE | CES | Weighted mode | P >108 | 13 | 0.0325823 | 0.200509 | 0.162497885 | 0.873618 |  | 1336.15 | 0.02067621 |  |  |  |  |
| Cannabis use disorder | MEGASTROKE | LAS | MR Egger | P >108 | 13 | -0.357472 | 0.560864 | -0.637360022 | 0.53693047 |  | 1336.15 | 0.02067621 | 0.04266783 | 0.03518386 | 0.2506422 | 0.423 |
| Cannabis use disorder | MEGASTROKE | LAS | Weighted median | P >108 | 13 | 0.2449139 | 0.186339 | 1.314349423 | 0.18872868 |  | 1336.15 | 0.02067621 |  |  |  |  |
| Cannabis use disorder | MEGASTROKE | LAS | Inverse variance  weighted | P >108 | 13 | 0.3002358 | 0.145708 | 2.060532064 | **0.0083452** | **0.0292082** | 1336.15 | 0.02067621 |  |  |  |  |
| Cannabis use disorder | MEGASTROKE | LAS | Simple mode | P >108 | 13 | 0.1517877 | 0.337941 | 0.449154172 | 0.78432459 |  | 1336.15 | 0.02067621 |  |  |  |  |
| Cannabis use disorder | MEGASTROKE | LAS | Weighted mode | P >108 | 13 | 0.1056275 | 0.288005 | 0.366755403 | 0.72018693 |  | 1336.15 | 0.02067621 |  |  |  |  |
| Cannabis use disorder | MEGASTROKE | SVS | MR Egger | P >108 | 13 | 0.1503774 | 0.537811 | 0.279609912 | 0.7849645 |  | 1336.15 | 0.02067621 | -0.01342086 | 0.03389818 | 0.6997375 | 0.237 |
| Cannabis use disorder | MEGASTROKE | SVS | Weighted median | P >108 | 13 | -0.027693 | 0.167001 | -0.165825873 | 0.83457635 |  | 1336.15 | 0.02067621 |  |  |  |  |
| Cannabis use disorder | MEGASTROKE | SVS | Inverse variance  weighted | P >108 | 13 | -0.055267 | 0.134475 | -0.410986966 | 0.3810821 | 0.53351494 | 1336.15 | 0.02067621 |  |  |  |  |
| Cannabis use disorder | MEGASTROKE | SVS | Simple mode | P >108 | 13 | 0.201987 | 0.278084 | 0.726352812 | 0.4815509 |  | 1336.15 | 0.02067621 |  |  |  |  |
| Cannabis use disorder | MEGASTROKE | SVS | Weighted mode | P >108 | 13 | 0.1421783 | 0.24603 | 0.577890361 | 0.8567577 |  | 1336.15 | 0.02067621 |  |  |  |  |
| Cannabis use disorder | Traylor et al, 2021 | SVS-MRI | MR Egger | P >108 | 13 | -0.245616 | 0.411375 | -0.597059886 | 0.5625551 |  | 1359.126 | 0.01955189 | 0.01882992 | 0.02590103 | 0.4824144 | 0.859 |
| Cannabis use disorder | Traylor et al, 2021 | SVS-MRI | Weighted median | P >108 | 13 | 0.1039446 | 0.144073 | 0.721471977 | 0.4706192 |  | 1359.126 | 0.01955189 |  |  |  |  |
| Cannabis use disorder | Traylor et al, 2021 | SVS-MRI | Inverse variance  weighted | P >108 | 13 | 0.0433586 | 0.105973 | 0.409147716 | 0.6645456 | 0.7534563 | 1359.126 | 0.01955189 |  |  |  |  |
| Cannabis use disorder | Traylor et al, 2021 | SVS-MRI | Simple mode | P >108 | 13 | 0.1373377 | 0.23588 | 0.58223665 | 0.5711848 |  | 1359.126 | 0.01955189 |  |  |  |  |
| Cannabis use disorder | Traylor et al, 2021 | SVS-MRI | Weighted mode | P >108 | 13 | 0.1252238 | 0.207276 | 0.604140858 | 0.6346546 |  | 1359.126 | 0.01955189 |  |  |  |  |
| Cannabis use disorder | Woo et al, 2014 | ICH | MR Egger | P >108 | 11 | -0.577642 | 1.561802 | -0.369856135 | 0.720043 |  | 1423.946 | 0.01737142 | 0.04802241 | 0.1029737 | 0.6520423 | 0.761 |
| Cannabis use disorder | Woo et al, 2014 | ICH | Weighted median | P >108 | 11 | -0.297992 | 0.4104 | -0.726102096 | 0.4677763 |  | 1423.946 | 0.01737142 |  |  |  |  |
| Cannabis use disorder | Woo et al, 2014 | ICH | Inverse variance  weighted | P >108 | 11 | 0.1244933 | 0.398707 | 0.312242261 | 0.7548564 | 0.7975436 | 1423.946 | 0.01737142 |  |  |  |  |
| Cannabis use disorder | Woo et al, 2014 | ICH | Simple mode | P >108 | 11 | -0.764834 | 0.934614 | -0.818341972 | 0.4322298 |  | 1423.946 | 0.01737142 |  |  |  |  |
| Cannabis use disorder | Woo et al, 2014 | ICH | Weighted mode | P >108 | 11 | -0.731984 | 0.544726 | -1.343765369 | 0.38736455 |  | 1423.946 | 0.01737142 |  |  |  |  |
| Problematic opioid use | MEGASTROKE | AS | MR Egger | P >108 | 90 | 0.1247739 | 0.064182 | 1.944055716 | 0.06465646 |  | 139.2664 | 0.08758634 | -0.004870765 | 0.003898573 | 0.2148051 | 0.522 |
| Problematic opioid use | MEGASTROKE | AS | Weighted median | P >108 | 90 | 0.0285587 | 0.035503 | 0.804403351 | 0.42116411 |  | 139.2664 | 0.08758634 |  |  |  |  |
| Problematic opioid use | MEGASTROKE | AS | Inverse variance  weighted | P >108 | 90 | 0.0517522 | 0.024151 | 2.142816566 | **0.004212783** | **0.02948948** | 139.2664 | 0.08758634 |  |  |  |  |
| Problematic opioid use | MEGASTROKE | AS | Simple mode | P >108 | 90 | 0.0473758 | 0.087906 | 0.538934008 | 0.59127796 |  | 139.2664 | 0.08758634 |  |  |  |  |
| Problematic opioid use | MEGASTROKE | AS | Weighted mode | P >108 | 90 | 0.0346909 | 0.071812 | 0.483076263 | 0.74646333 |  | 139.2664 | 0.08758634 |  |  |  |  |
| Problematic opioid use | MEGASTROKE | IS | MR Egger | P >108 | 90 | 0.135553 | 0.070007 | 1.936280129 | 0.05604279 |  | 139.2664 | 0.08758634 | -0.005600297 | 0.00425404 | 0.1913981 | 0.182 |
| Problematic opioid use | MEGASTROKE | IS | Weighted median | P >108 | 90 | 0.0601814 | 0.039153 | 1.537099901 | 0.12426878 |  | 139.2664 | 0.08758634 |  |  |  |  |
| Problematic opioid use | MEGASTROKE | IS | Inverse variance  weighted | P >108 | 90 | 0.0532384 | 0.0263 | 2.024242198 | **0.009294524** | **0.03253083** | 139.2664 | 0.08758634 |  |  |  |  |
| Problematic opioid use | MEGASTROKE | IS | Simple mode | P >108 | 90 | 0.0834687 | 0.088836 | 0.93958656 | 0.34997279 |  | 139.2664 | 0.08758634 |  |  |  |  |
| Problematic opioid use | MEGASTROKE | IS | Weighted mode | P >108 | 90 | 0.0690509 | 0.074969 | 0.921056545 | 0.35951087 |  | 139.2664 | 0.08758634 |  |  |  |  |
| Problematic opioid use | MEGASTROKE | CES | MR Egger | P >108 | 90 | 0.470005 | 0.163485 | 2.874912591 | 0.005067265 |  | 139.2664 | 0.08758634 | -0.02933939 | 0.009823372 | **0.003642731** | 0.082 |
| Problematic opioid use | MEGASTROKE | CES | Weighted median | P >108 | 90 | -0.035804 | 0.079468 | -0.4505451 | 0.738563355 |  | 139.2664 | 0.08758634 |  |  |  |  |
| Problematic opioid use | MEGASTROKE | CES | Inverse variance  weighted | P >108 | 90 | 0.0114408 | 0.063348 | 0.180601657 | 0.756680224 | 0.8934563 | 139.2664 | 0.08758634 |  |  |  |  |
| Problematic opioid use | MEGASTROKE | CES | Simple mode | P >108 | 90 | -0.080345 | 0.198769 | -0.404213544 | 0.687025397 |  | 139.2664 | 0.08758634 |  |  |  |  |
| Problematic opioid use | MEGASTROKE | CES | Weighted mode | P >108 | 90 | -0.05188 | 0.156046 | -0.332466365 | 0.740318958 |  | 139.2664 | 0.08758634 |  |  |  |  |
| Problematic opioid use | MEGASTROKE | LAS | MR Egger | P >108 | 90 | 0.2948311 | 0.199642 | 1.476800199 | 0.53432989 |  | 139.2664 | 0.08758634 | -0.01374812 | 0.01202894 | 0.2561372 | 0.323 |
| Problematic opioid use | MEGASTROKE | LAS | Weighted median | P >108 | 90 | 0.0904283 | 0.099857 | 0.905579284 | 0.3651586 |  | 139.2664 | 0.08758634 |  |  |  |  |
| Problematic opioid use | MEGASTROKE | LAS | Inverse variance  weighted | P >108 | 90 | 0.1045775 | 0.074049 | 1.412266001 | 0.0534517 | 0.12472063 | 139.2664 | 0.08758634 |  |  |  |  |
| Problematic opioid use | MEGASTROKE | LAS | Simple mode | P >108 | 90 | 0.1499013 | 0.262223 | 0.571656676 | 0.5689955 |  | 139.2664 | 0.08758634 |  |  |  |  |
| Problematic opioid use | MEGASTROKE | LAS | Weighted mode | P >108 | 90 | 0.0967316 | 0.195117 | 0.495762538 | 0.6212839 |  | 139.2664 | 0.08758634 |  |  |  |  |
| Problematic opioid use | MEGASTROKE | SVS | MR Egger | P >108 | 90 | -0.066323 | 0.16632 | -0.398769564 | 0.3640181 |  | 139.2664 | 0.08758634 | 0.00685079 | 0.01003019 | 0.4963507 | 0.411 |
| Problematic opioid use | MEGASTROKE | SVS | Weighted median | P >108 | 90 | 0.0683924 | 0.094667 | 0.722456242 | 0.470014 |  | 139.2664 | 0.08758634 |  |  |  |  |
| Problematic opioid use | MEGASTROKE | SVS | Inverse variance  weighted | P >108 | 90 | 0.0331471 | 0.062098 | 0.533789077 | 0.5934874 | 0.69240196 | 139.2664 | 0.08758634 |  |  |  |  |
| Problematic opioid use | MEGASTROKE | SVS | Simple mode | P >108 | 90 | 0.1131074 | 0.229091 | 0.493721678 | 0.7464224 |  | 139.2664 | 0.08758634 |  |  |  |  |
| Problematic opioid use | MEGASTROKE | SVS | Weighted mode | P >108 | 90 | 0.0546859 | 0.16822 | 0.325086088 | 0.7458704 |  | 139.2664 | 0.08758634 |  |  |  |  |
| Problematic opioid use | Traylor et al, 2021 | SVS-MRI | MR Egger | P >108 | 78 | -0.052247 | 0.148738 | -0.351267108 | 0.6453599 |  | 139.7059 | 0.08783846 | 0.006578337 | 0.01019852 | 0.5205681 | 0.380 |
| Problematic opioid use | Traylor et al, 2021 | SVS-MRI | Weighted median | P >108 | 78 | -0.060413 | 0.081935 | -0.737326162 | 0.460924 |  | 139.7059 | 0.08783846 |  |  |  |  |
| Problematic opioid use | Traylor et al, 2021 | SVS-MRI | Inverse variance  weighted | P >108 | 78 | -0.059371 | 0.056614 | -1.048700834 | 0.2943158 | 0.51505265 | 139.7059 | 0.08783846 |  |  |  |  |
| Problematic opioid use | Traylor et al, 2021 | SVS-MRI | Simple mode | P >108 | 78 | -0.241205 | 0.189226 | -1.27468927 | 0.3837555 |  | 139.7059 | 0.08783846 |  |  |  |  |
| Problematic opioid use | Traylor et al, 2021 | SVS-MRI | Weighted mode | P >108 | 78 | -0.108803 | 0.154932 | -0.702260162 | 0.2464564 |  | 139.7059 | 0.08783846 |  |  |  |  |
| Problematic opioid use | Woo et al, 2014 | SVS-MRI | MR Egger | P >108 | 46 | -0.177884 | 0.527938 | -0.336941402 | 0.7377631 |  | 129.1463 | 0.04304666 | 0.004217761 | 0.03191262 | 0.8954556 | 0.599 |
| Problematic opioid use | Woo et al, 2014 | ICH | Weighted median | P >108 | 46 | -0.072211 | 0.271294 | -0.266172849 | 0.7901061 |  | 129.1463 | 0.04304666 |  |  |  |  |
| Problematic opioid use | Woo et al, 2014 | ICH | Inverse variance  weighted | P >108 | 46 | -0.112171 | 0.177509 | -0.631917649 | 0.5274408 | 0.69240196 | 129.1463 | 0.04304666 |  |  |  |  |
| Problematic opioid use | Woo et al, 2014 | ICH | Simple mode | P >108 | 46 | 0.380287 | 0.541621 | 0.702127521 | 0.4862157 |  | 129.1463 | 0.04304666 |  |  |  |  |
| Problematic opioid use | Woo et al, 2014 | ICH | Weighted mode | P >108 | 46 | 0.1386942 | 0.453828 | 0.305609682 | 0.761311 |  | 129.1463 | 0.04304666 |  |  |  |  |
| Substance abuse disorder | MEGASTROKE | AS | MR Egger | P >107 | 27 | -0.194305 | 0.644962 | -0.301265176 | 0.6456464 |  | 90.84258 | 0.002397664 | -0.007078102 | 0.009381908 | 0.7657039 | 0.499 |
| Substance abuse disorder | MEGASTROKE | AS | Weighted median | P >107 | 27 | 0.2540662 | 0.185962 | 1.366228637 | 0.235354 |  | 90.84258 | 0.002397664 |  |  |  |  |
| Substance use disorder | MEGASTROKE | AS | Inverse variance  weighted | P >107 | 27 | 0.2815927 | 0.133324 | 2.112101017 | **0.00267776** | **0.01874432** | 90.84258 | 0.002397664 |  |  |  |  |
| Substance use disorder | MEGASTROKE | AS | Simple mode | P >107 | 27 | 0.5558233 | 0.411944 | 1.349268748 | 0.18888411 |  | 90.84258 | 0.002397664 |  |  |  |  |
| Substance use disorder | MEGASTROKE | AS | Weighted mode | P >107 | 27 | 0.5377876 | 0.407275 | 1.320453905 | 0.21879003 |  | 90.84258 | 0.002397664 |  |  |  |  |
| Substance use disorder | MEGASTROKE | IS | MR Egger | P >107 | 27 | 0.0063463 | 0.726634 | 0.008733824 | 0.99310083 |  | 90.84258 | 0.002397664 | 0.003614181 | 0.7266341 | 0.9931008 | 0.367 |
| Substance use disorder | MEGASTROKE | IS | Weighted median | P >107 | 27 | 0.3236463 | 0.216648 | 1.493881061 | 0.29464446 |  | 90.84258 | 0.002397664 |  |  |  |  |
| Substance use disorder | MEGASTROKE | IS | Inverse variance  weighted | P >107 | 27 | 0.2495103 | 0.148889 | 1.675816703 | 0.05377412 | 0.12547294 | 90.84258 | 0.002397664 |  |  |  |  |
| Substance use disorder | MEGASTROKE | IS | Simple mode | P >107 | 27 | 0.9639584 | 0.493922 | 1.951640163 | 0.06183152 |  | 90.84258 | 0.002397664 |  |  |  |  |
| Substance use disorder | MEGASTROKE | IS | Weighted mode | P >107 | 27 | -0.311966 | 0.504605 | -0.61823922 | 0.66757775 |  | 90.84258 | 0.002397664 |  |  |  |  |
| Substance use disorder | MEGASTROKE | CES | MR Egger | P >107 | 27 | 0.186138 | 1.383678 | 0.134524034 | 0.8940654 |  | 90.84258 | 0.002397664 | 0.002527399 | 0.02000897 | 0.9004943 | 0.810 |
| Substance use disorder | MEGASTROKE | CES | Weighted median | P >107 | 27 | 0.2576317 | 0.375913 | 0.685349087 | 0.4931236 |  | 90.84258 | 0.002397664 |  |  |  |  |
| Substance use disorder | MEGASTROKE | CES | Inverse variance  weighted | P >107 | 27 | 0.3573693 | 0.277294 | 1.288776333 | 0.2378456 | 0.34558265 | 90.84258 | 0.002397664 |  |  |  |  |
| Substance use disorder | MEGASTROKE | CES | Simple mode | P >107 | 27 | 0.2216595 | 0.775408 | 0.285861618 | 0.7772493 |  | 90.84258 | 0.002397664 |  |  |  |  |
| Substance use disorder | MEGASTROKE | CES | Weighted mode | P >107 | 27 | 0.2216595 | 0.776067 | 0.285619172 | 0.6357355 |  | 90.84258 | 0.002397664 |  |  |  |  |
| Substance use disorder | MEGASTROKE | LAS | MR Egger | P >107 | 27 | -1.396422 | 2.241956 | -0.622858698 | 0.5390182 |  | 90.84258 | 0.002397664 | 0.02468625 | 0.03257871 | 0.4556892 | 0.055 |
| Substance use disorder | MEGASTROKE | LAS | Weighted median | P >107 | 27 | 0.2762725 | 0.559384 | 0.493886885 | 0.6213861 |  | 90.84258 | 0.002397664 |  |  |  |  |
| Substance use disorder | MEGASTROKE | LAS | Inverse variance  weighted | P >107 | 27 | 0.2649483 | 0.464326 | 0.570608836 | 0.5682648 | 0.66834554 | 90.84258 | 0.002397664 |  |  |  |  |
| Substance use disorder | MEGASTROKE | LAS | Simple mode | P >107 | 27 | -0.073907 | 1.2992 | -0.056886697 | 0.9550704 |  | 90.84258 | 0.002397664 |  |  |  |  |
| Substance use disorder | MEGASTROKE | LAS | Weighted mode | P >107 | 27 | -0.334481 | 1.262353 | -0.264966049 | 0.793125 |  | 90.84258 | 0.002397664 |  |  |  |  |
| Substance use disorder | MEGASTROKE | SVS | MR Egger | P >107 | 27 | -1.914147 | 2.656277 | -0.720612835 | 0.4245355 |  | 90.84258 | 0.002397664 | -0.05852013 | 0.08331409 | 0.4970222 | 0.573 |
| Substance use disorder | MEGASTROKE | SVS | Weighted median | P >107 | 27 | -0.159053 | 0.554425 | -0.286878633 | 0.7742053 |  | 90.84258 | 0.002397664 |  |  |  |  |
| Substance use disorder | MEGASTROKE | SVS | Inverse variance  weighted | P >107 | 27 | 0.1607548 | 0.551118 | 0.291688637 | 0.4742053 | 0.55323951 | 90.84258 | 0.002397664 |  |  |  |  |
| Substance use disorder | MEGASTROKE | SVS | Simple mode | P >107 | 27 | -0.535284 | 1.51272 | -0.353855019 | 0.726301 |  | 90.84258 | 0.002397664 |  |  |  |  |
| Substance use disorder | MEGASTROKE | SVS | Weighted mode | P >107 | 27 | -0.754762 | 1.231518 | -0.612871094 | 0.5452856 |  | 90.84258 | 0.002397664 |  |  |  |  |
| Substance use disorder | Traylor et al, 2021 | SVS-MRI | MR Egger | P >107 | 27 | -1.820238 | 2.159413 | -0.842931838 | 0.4072593 |  | 91.28766 | 0.002397664 | 0.03270467 | 0.03149574 | 0.3090308 | 0.380 |
| Substance use disorder | Traylor et al, 2021 | SVS-MRI | Weighted median | P >107 | 27 | 0.2186698 | 0.480072 | 0.455493854 | 0.44654765 |  | 91.28766 | 0.002397664 |  |  |  |  |
| Substance use disorder | Traylor et al, 2021 | SVS-MRI | Inverse variance  weighted | P >107 | 27 | 0.371372 | 0.457243 | 0.812198505 | 0.4166777 | 0.55323951 | 91.28766 | 0.002397664 |  |  |  |  |
| Substance use disorder | Traylor et al, 2021 | SVS-MRI | Simple mode | P >107 | 27 | 1.4389372 | 1.231388 | 1.168549367 | 0.2531885 |  | 91.28766 | 0.002397664 |  |  |  |  |
| Substance use disorder | Traylor et al, 2021 | SVS-MRI | Weighted mode | P >107 | 27 | 1.1491941 | 1.201678 | 0.956324251 | 0.2356365 |  | 91.28766 | 0.002397664 |  |  |  |  |
| Substance use disorder | Woo et al, 2014 | ICH | MR Egger | P >107 | 25 | -0.330791 | 5.786983 | -0.057161222 | 0.95491046 |  | 89.14342 | 0.002168407 | 0.03436964 | 0.08237414 | 0.6803731 | 0.104 |
| Substance use disorder | Woo et al, 2014 | ICH | Weighted median | P >107 | 25 | 2.048304 | 1.103992 | 1.855360598 | 0.05945345 |  | 89.14342 | 0.002168407 |  |  |  |  |
| Substance use disorder | Woo et al, 2014 | ICH | Inverse variance  weighted | P >107 | 25 | 2.052339 | 0.414393 | 4.952642747 | **0.00948013** | **0.03318046** | 89.14342 | 0.002168407 |  |  |  |  |
| Substance use disorder | Woo et al, 2014 | ICH | Simple mode | P >107 | 25 | 1.918338 | 2.212328 | 0.867112912 | 0.39446735 |  | 89.14342 | 0.002168407 |  |  |  |  |
| Substance use disorder | Woo et al, 2014 | ICH | Weighted mode | P >107 | 25 | 2.161174 | 1.980223 | 1.091379157 | 0.19345355 |  | 89.14342 | 0.002168407 |  |  |  |  |


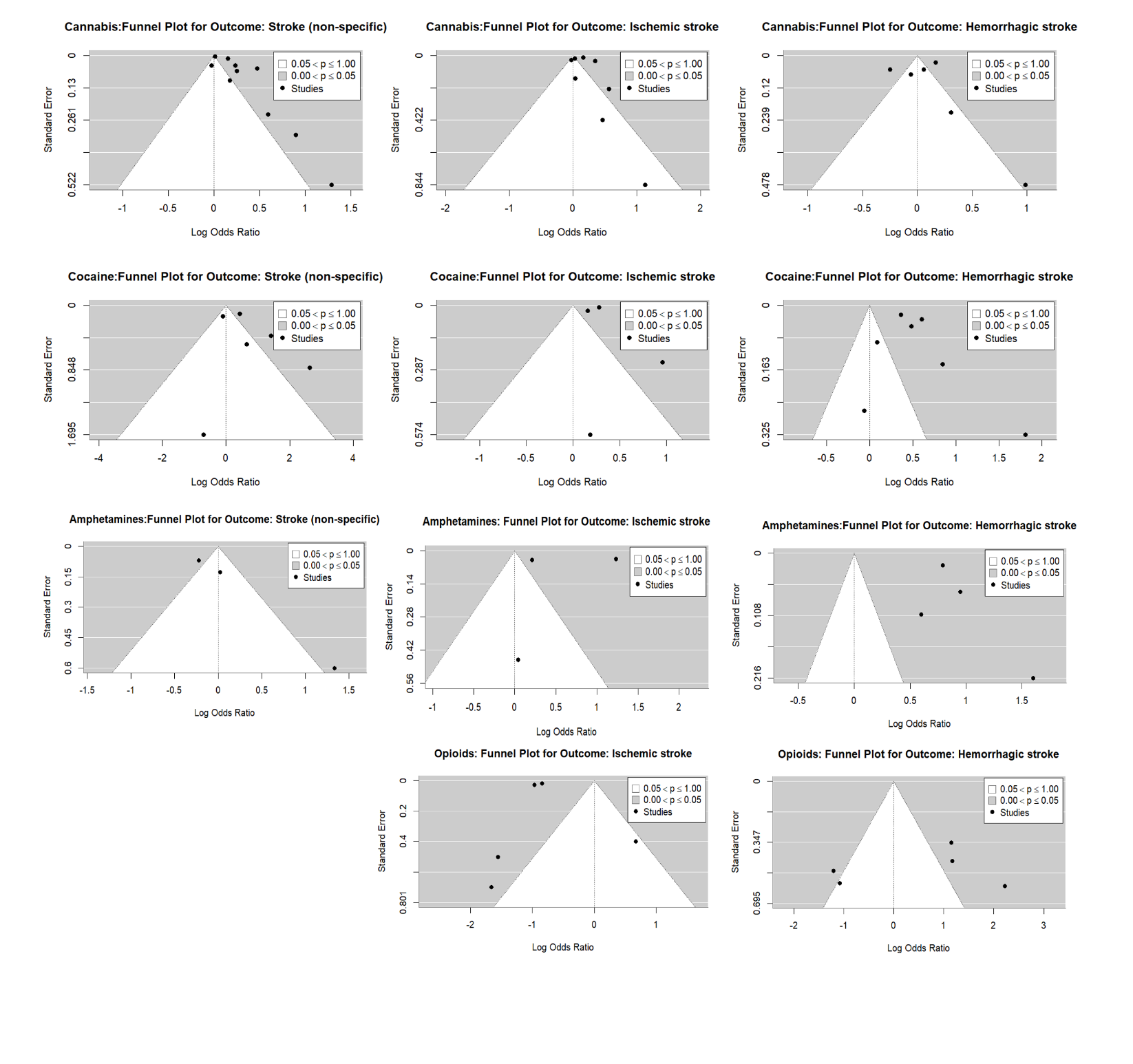


**Supplementary Figure 1:** Funnel plots showing publication bias assessment for each drug and stroke type.

**
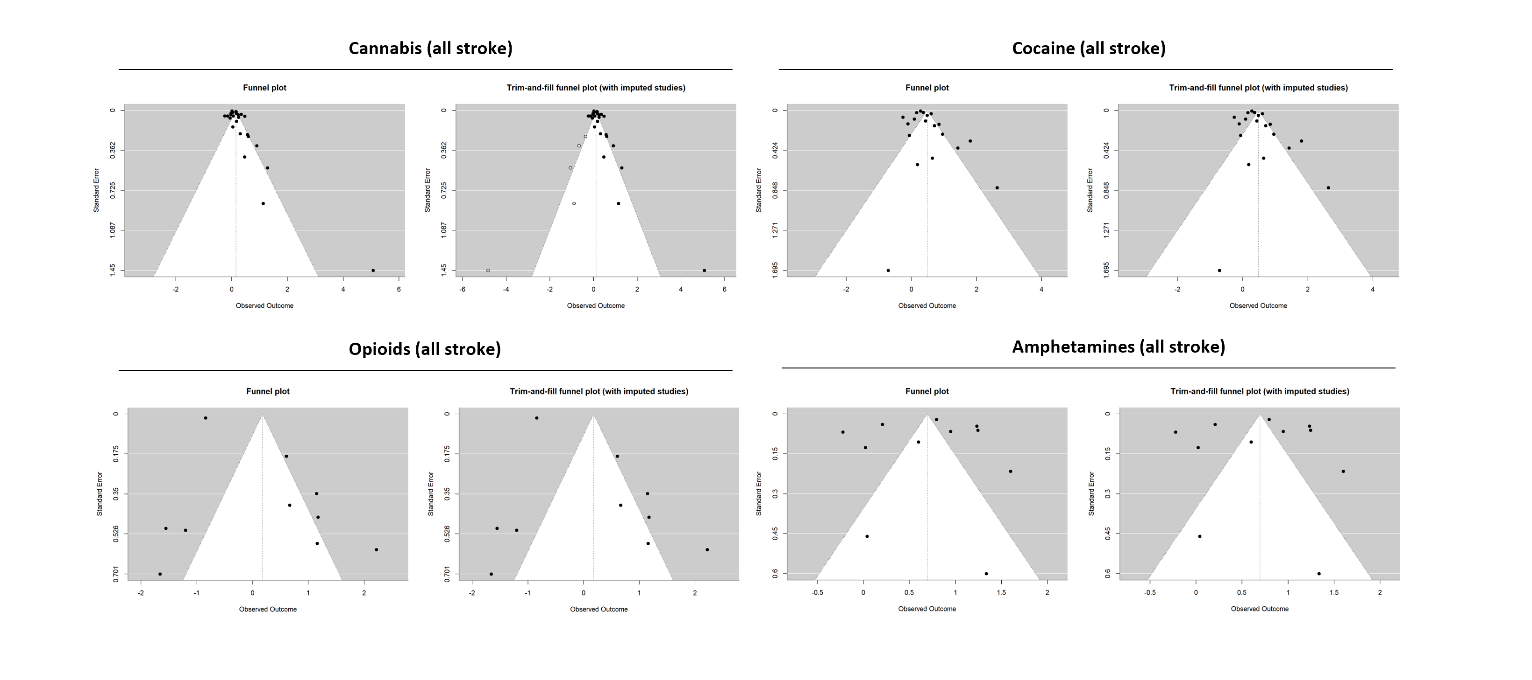
Supplementary Figure 2:** Funnel plots showing publication bias assessment for each drug for overall stroke, including trim-and-fill adjustments.


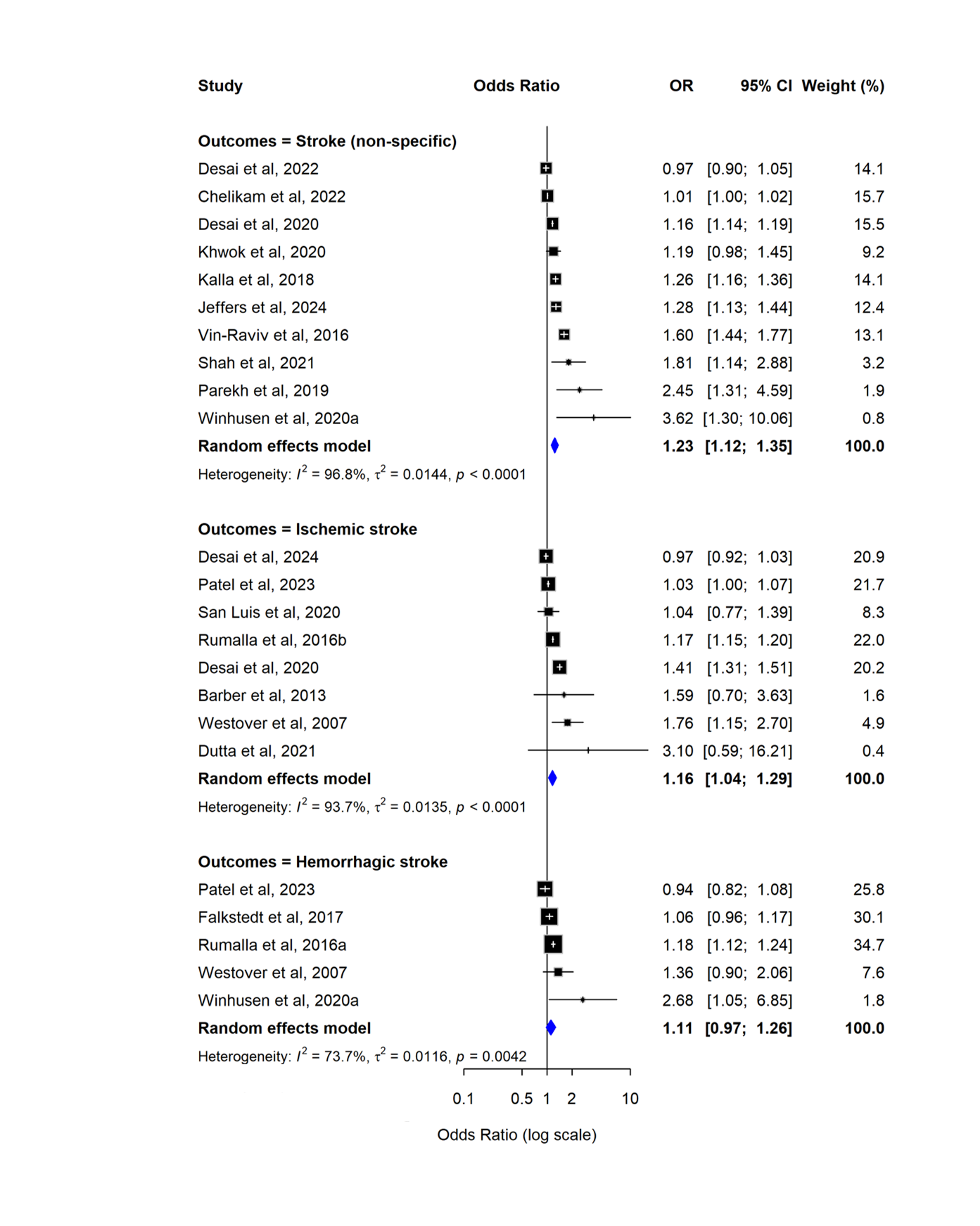


**Supplementary Figure 3. Forest plot from the univariate random-effects meta-analysis of cannabis and stroke risk.** Squares represent study-specific effect estimates, and diamonds represent the pooled multivariate estimates.


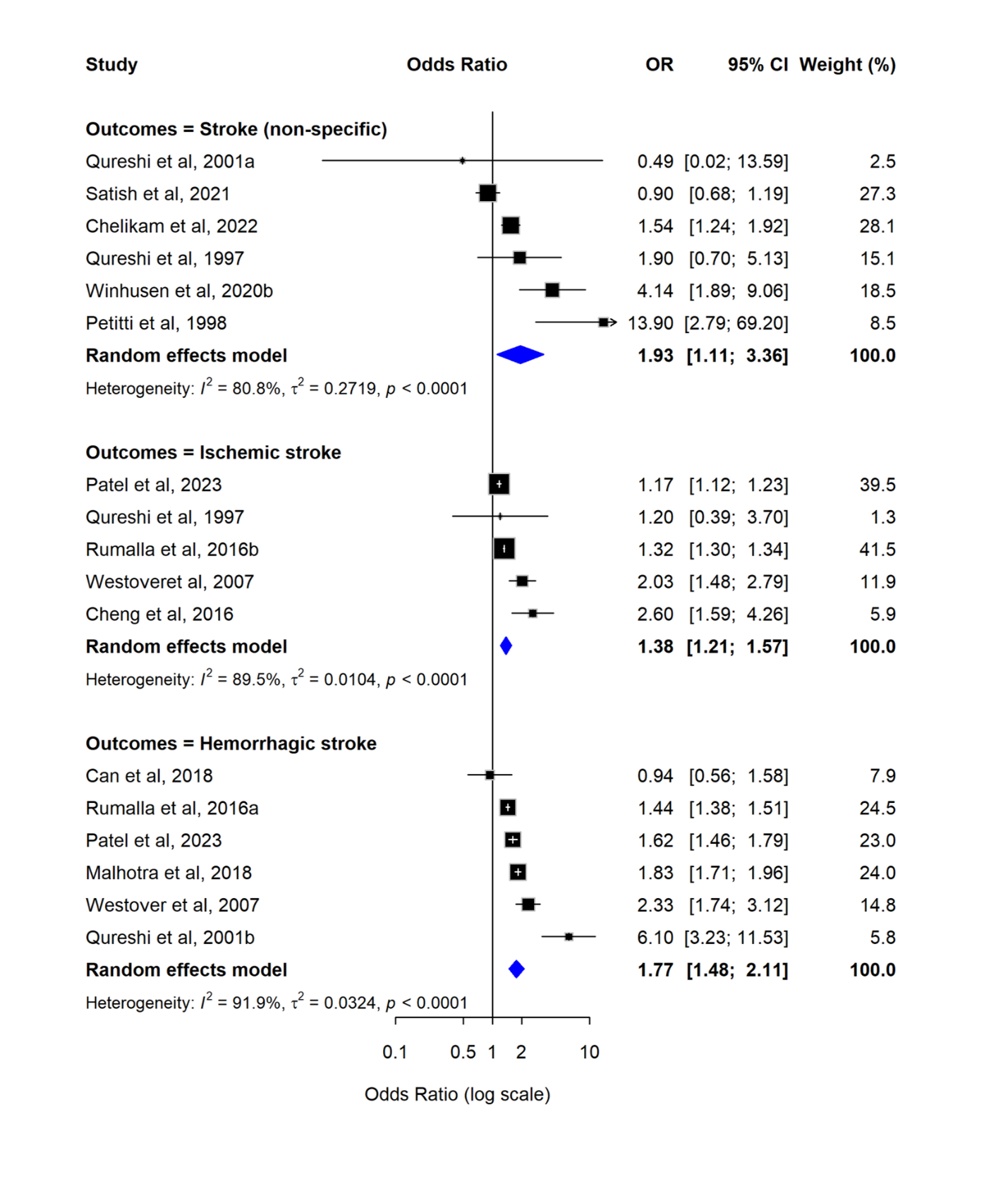


**Supplementary Figure 4. Forest plot from the univariate random-effects meta-analysis of cocaine and stroke risk.** Squares represent study-specific effect estimates, and diamonds represent the pooled multivariate estimates.


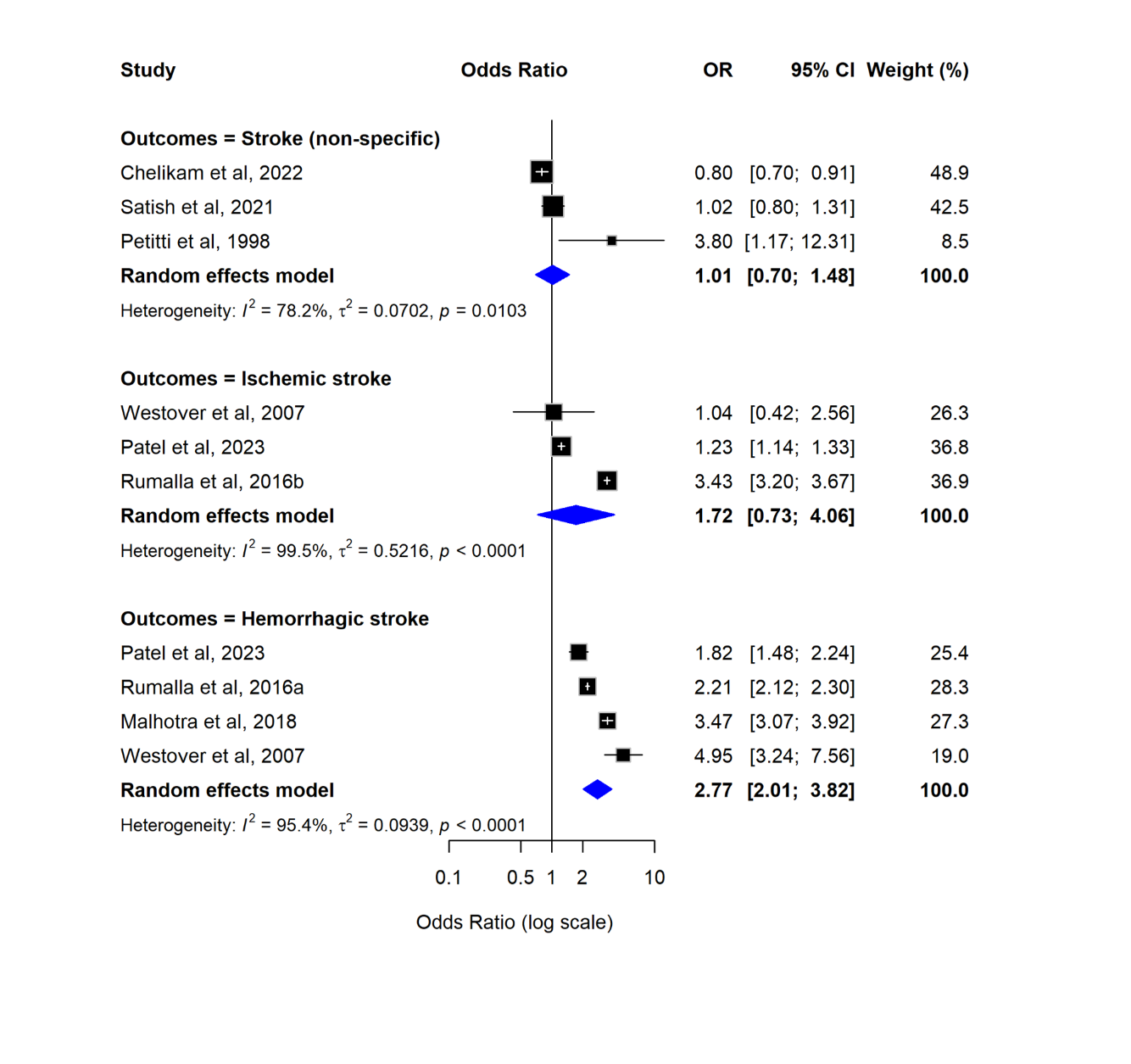


**Supplementary Figure 5. Forest plot from the univariate random-effects meta-analysis of amphetamines and stroke risk.** Squares represent study-specific effect estimates, and diamonds represent the pooled multivariate estimates.


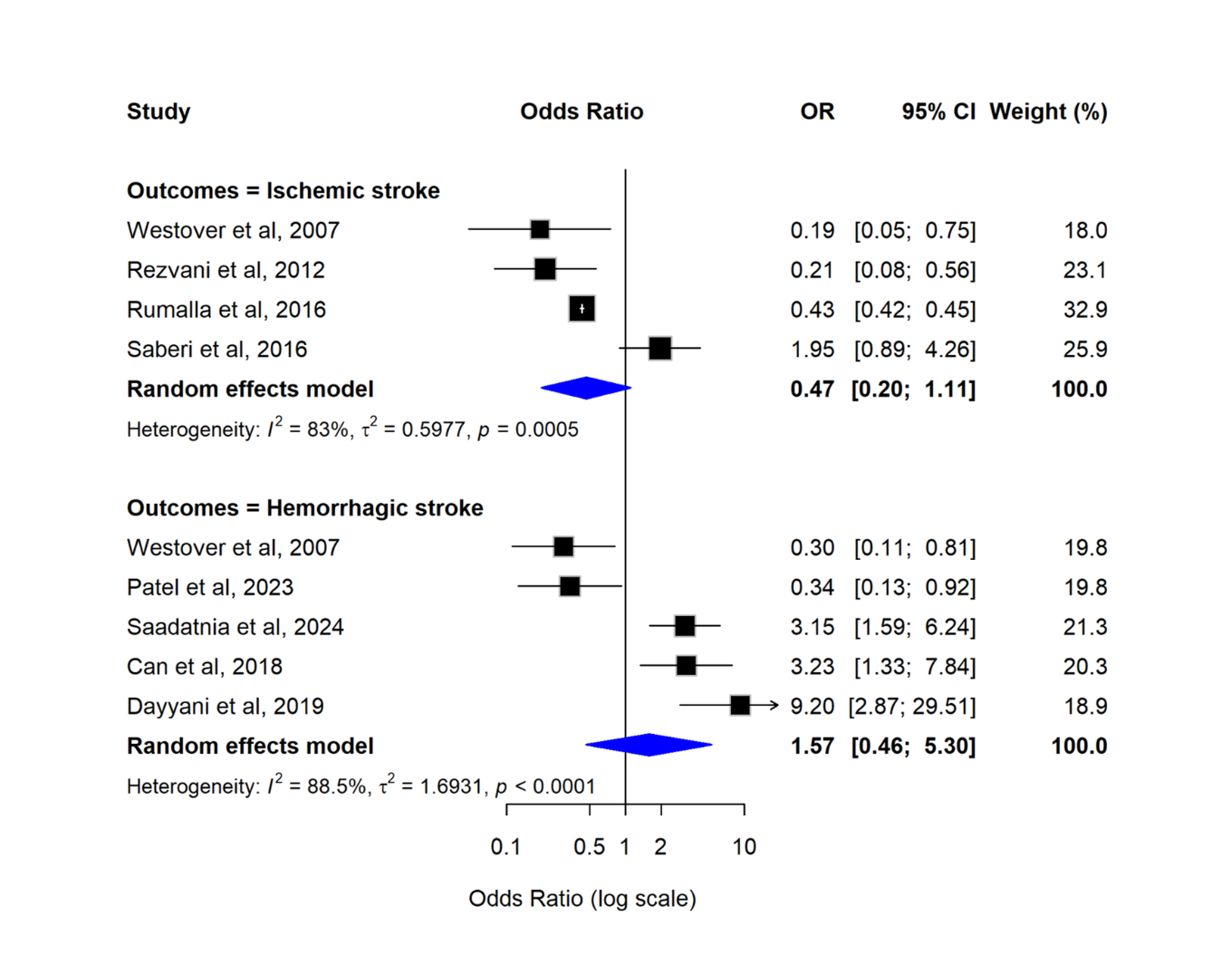


**Supplementary Figure 6. Forest plot from the univariate random-effects meta-analysis of opioids and stroke risk.** Squares represent study-specific effect estimates, and diamonds represent the pooled multivariate estimates.


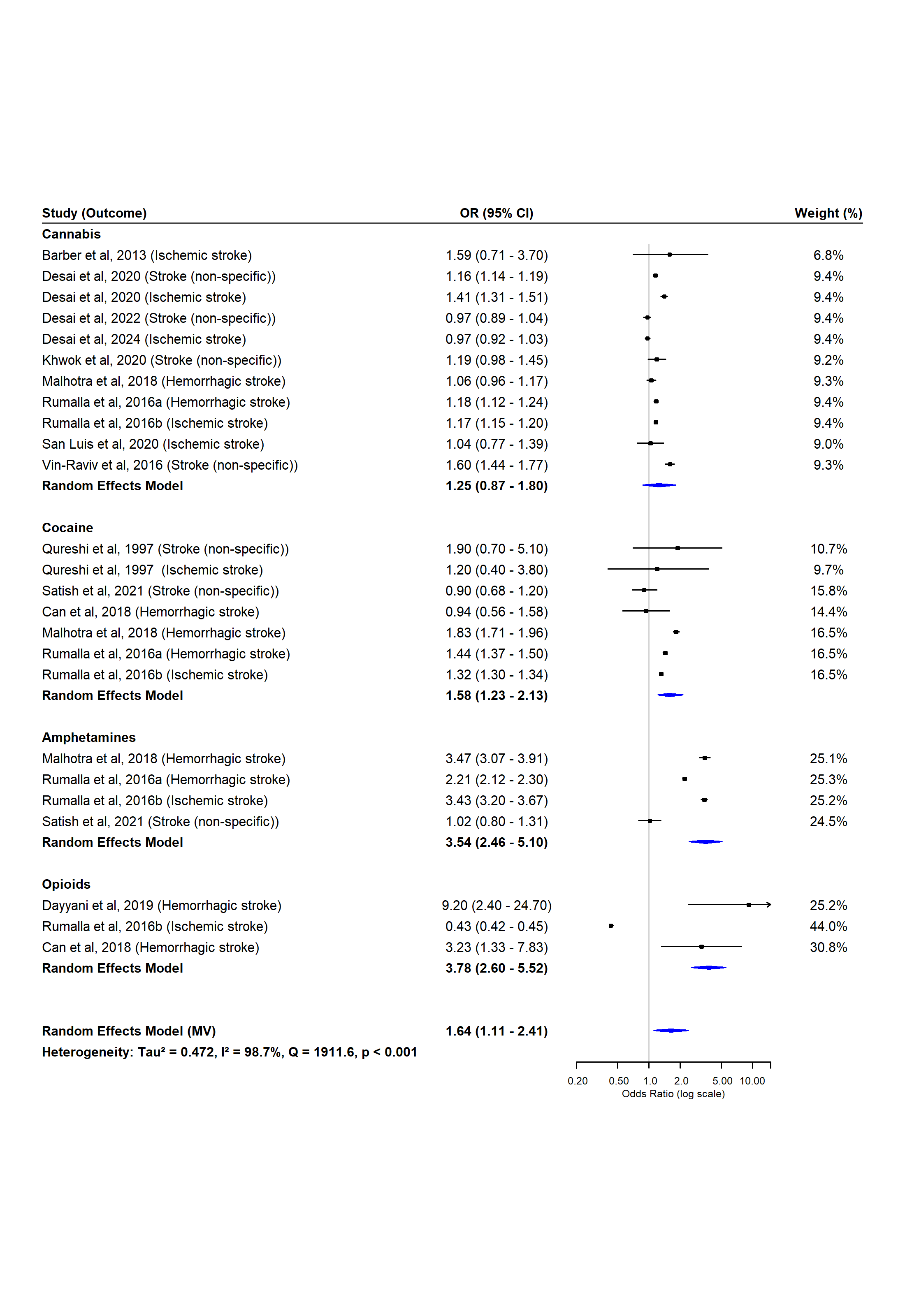


**Supplementary Figure 7.** Forest plot from the multivariate random-effects meta-analysis of recent illicit drug use and stroke risk. Squares indicate study-specific effect estimates, with size proportional to study weight, and diamonds represent the pooled multivariate estimates.


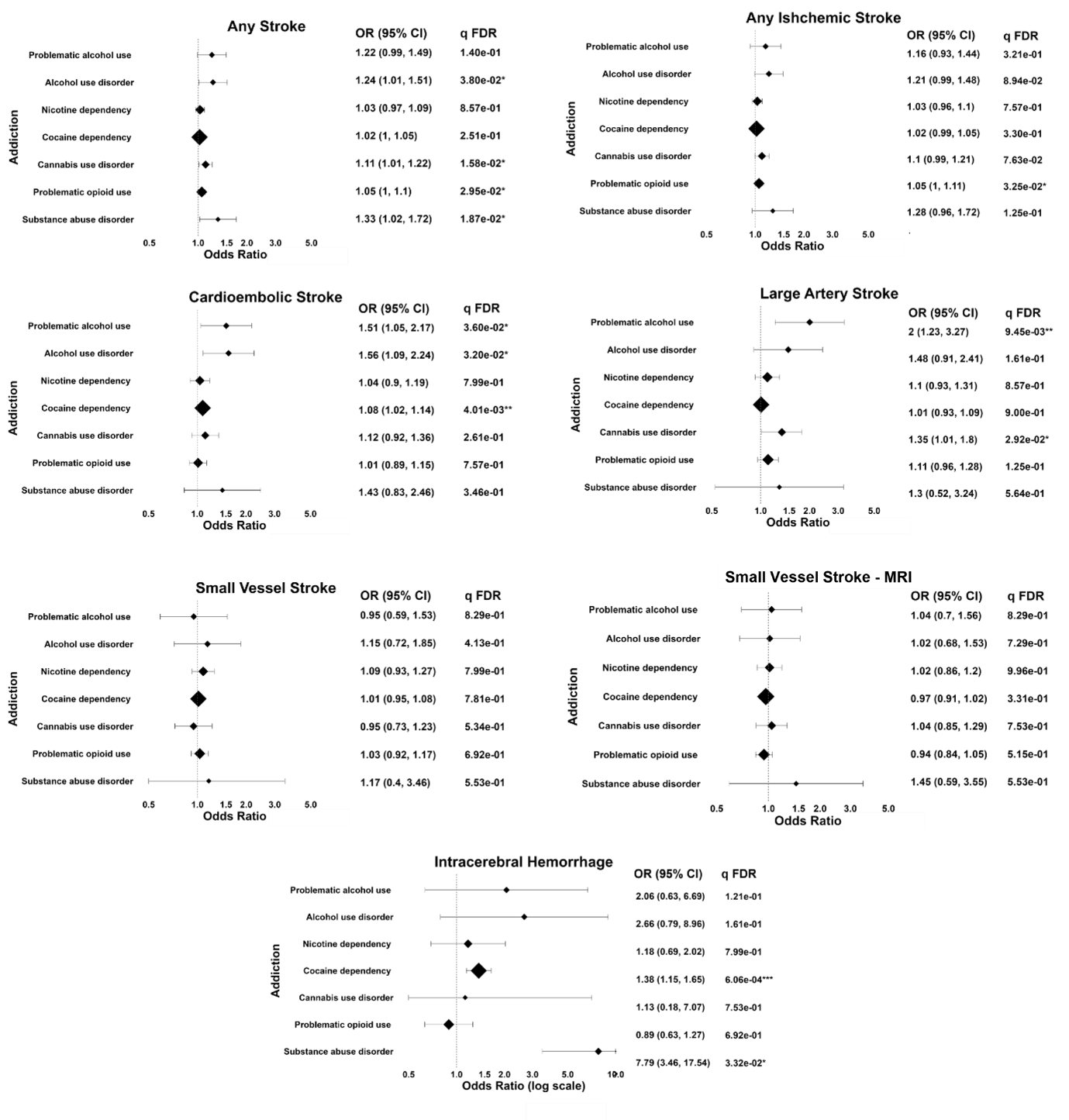


**Supplementary Figure 8. Mendelian randomisation associations between genetically predicted substance dependence and stroke subtypes.** Forest plots showing the results from the random-effects inverse-variance weighted Mendelian randomization analyses. Significant associations for the causal effect estimates are indicated with asterisks (*P*<0.05, ** *P*<0.01, *** *P*<0.001).


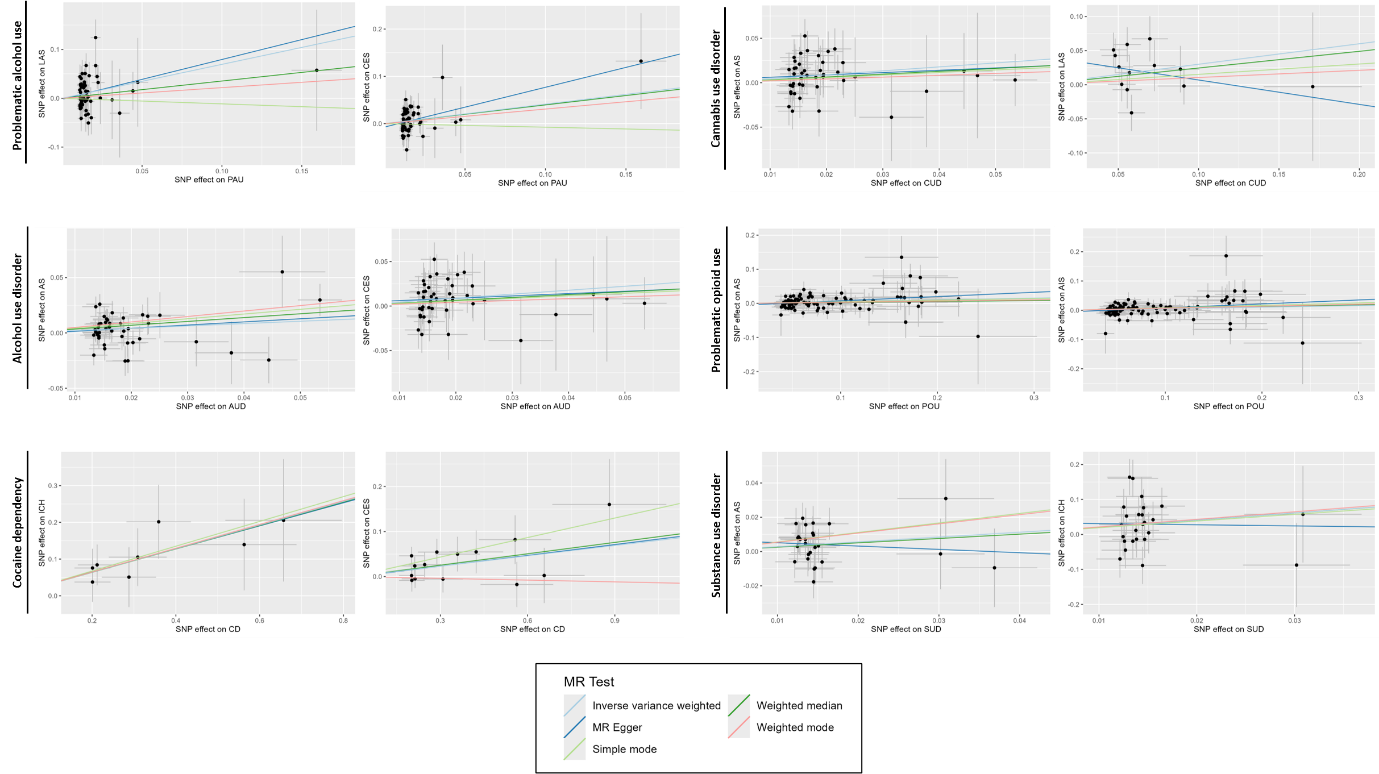


**Supplementary Figure 9. Scatter plots showing genetic associations of substance use exposures and stroke subtypes for significant causal estimates.** For traits with significant causal estimates (false discovery rate, *q*<0.05) each genetic variant is plotted by its association with the corresponding outcome. Data points represent the effect of each variant on the change in stroke risk, with horizontal and vertical lines denoting 95% confidence intervals (CIs). Coloured regression lines indicate causal estimates from different Mendelian randomization (MR) methods. Abbreviations: AS, all stroke; AIS, any ischemic stroke; CES, cardioembolic stroke; ICH, intracerebral haemorrhage and LAS, large artery stroke.


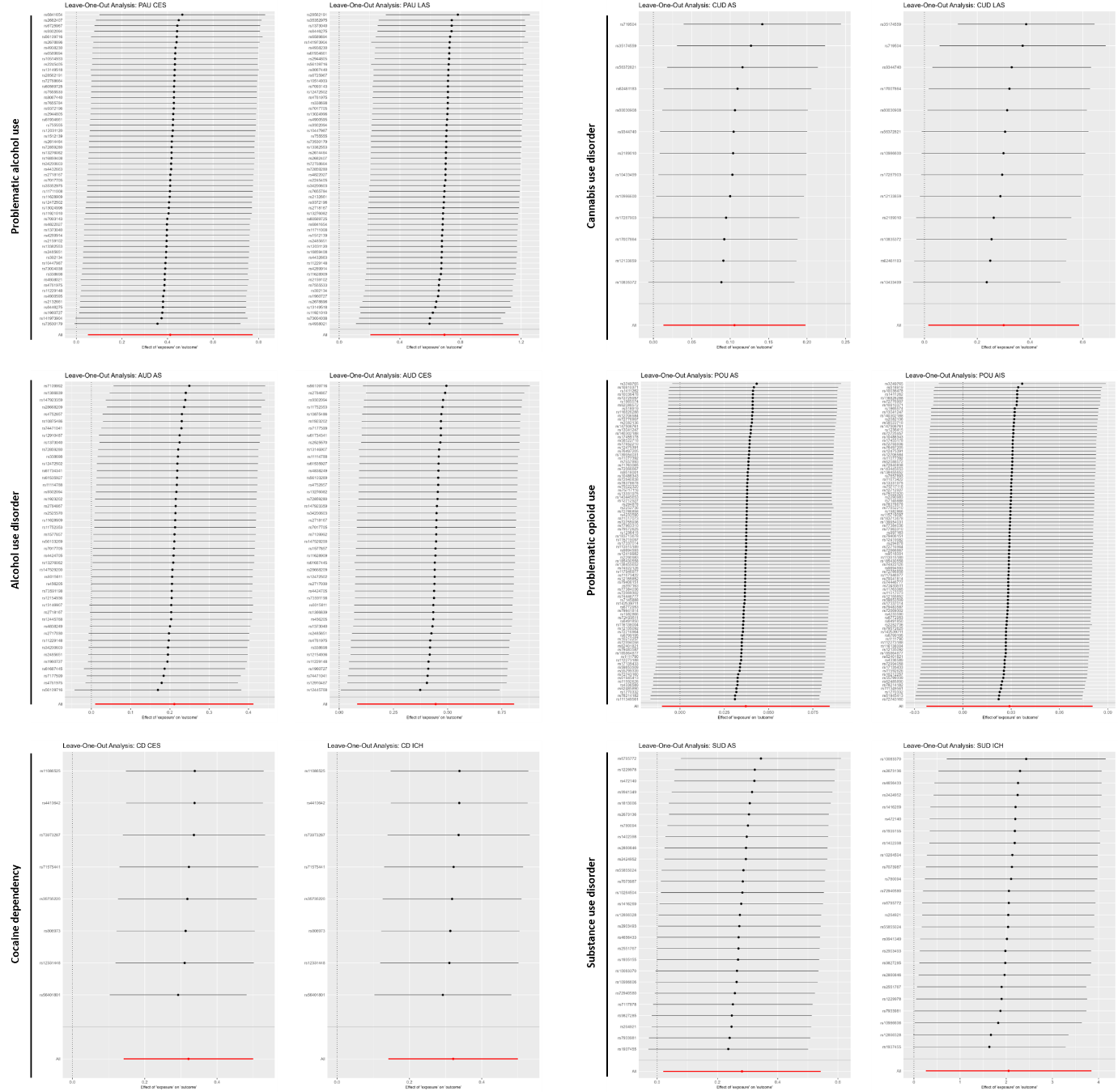


**Supplementary Figure 10. Leave-one-out analysis for all significant causal estimates identified by Mendelian Randomisation.**

**Supplementary references**

1. Zhou H, Kember RL, Deak JD, et al. Multi-ancestry study of the genetics of problematic alcohol use in over 1 million individuals. *Nat Med* 2023; 29(12):3184-3192.
2. Quach BC, Bray MJ, Gaddis NC, et al. Expanding the genetic architecture of nicotine dependence and its shared genetics with multiple traits. *Nat Commun* 2020; 11(1):5562.
3. Cabana-Domínguez J, Shivalikanjli A, Fernàndez-Castillo N, et al. Genome-wide association meta-analysis of cocaine dependence: shared genetics with comorbid conditions. *Prog Neuropsychopharmacol Biol Psychiatry* 2019; 94:109667.
4. Levey DF, Galimberti M, Deak JD, et al. Multi-ancestry genome-wide association study of cannabis use disorder yields insight into disease biology and public health implications. *Nat Genet 2023*; 55(12):2094-2103.
5. Sanchez-Roige S, Fontanillas P, Jennings MV, et al. Genome-wide association study of problematic opioid prescription use in 132,113 23andMe research participants of European ancestry. *Mol Psychiatry* 2021; 26(11):6209-6217.
6. Hatoum AS, Colbert SMC, Johnson EC, et al. Multivariate genome-wide association meta-analysis of over 1 million subjects identifies loci underlying multiple substance use disorders. *Nat Ment Health* 2023; 1(3):210-223.
7. Malik R, Chauhan G, Traylor M, et al. Multiancestry genome-wide association study of 520,000 subjects identifies 32 loci associated with stroke and stroke subtypes. *Nat Genet* 2018; 50:524–537.
8. Traylor M, Persyn E, Tomppo L, et al. Genetic basis of lacunar stroke: a pooled analysis of individual patient data and genome-wide association studies*. Lancet Neurol* 2021; 20(5):351–361.
9. Woo D, Falcone GJ, Devan WJ, et al. International Stroke Genetics Consortium. Meta-analysis of genome-wide association studies identifies 1q22 as a susceptibility locus for intracerebral hemorrhage. *Am J Hum Genet* 2014; 94:511–521.
10. Adams HP Jr et al. Classification of subtype of acute ischemic stroke. Definitions for use in a multicenter clinical trial. TOAST. Stroke. 1993;24:35–41.
11. Barber PA et al. Cannabis, ischemic stroke, and transient ischemic attack: a case-control study. Stroke. 2013;44(8):2327–2329.
12. Can A et al. Heroin use is associated with ruptured saccular aneurysms. Transl Stroke Res. 2018;9(4):340–346.
13. Chelikam N et al. Prevalence of cerebrovascular accidents among the US population with substance use disorders: a nationwide study. Cureus. 2022;14(11):e31826.
14. Cheng YC et al. Cocaine use and risk of ischemic stroke in young adults. Stroke. 2016;47(4):918–922.
15. Dayyani M et al. Association of opium addiction with rupture of intracranial aneurysms: a case-control study. World Neurosurg. 2019;126:e492–e499.
16. Desai R et al. Stroke in young cannabis users (18–49 years): national trends in hospitalizations and outcomes. Int J Stroke. 2020;15(5):535–539.
17. Desai R et al. Hypertensive crisis-related hospitalizations and subsequent major adverse cardiac events in young adults with cannabis use disorder: a nationwide analysis. Medicina (Kaunas). 2022;58(10):1465.
18. Desai R et al. Contemporary nationwide trends in major adverse cardiovascular events in young cannabis users without concomitant tobacco, alcohol, cocaine use. World J Cardiol. 2024;16(9):512–521.
19. Dutta T et al. Marijuana use and the risk of early ischemic stroke: the Stroke Prevention in Young Adults Study. Stroke. 2021;52(10):3184–3190.
20. Jeffers AM et al. Association of cannabis use with cardiovascular outcomes among US adults. J Am Heart Assoc. 2024;13(5):e030178.
21. Kalla A et al. Cannabis use predicts risks of heart failure and cerebrovascular accidents: results from the National Inpatient Sample. J Cardiovasc Med (Hagerstown). 2018;19(9):480–484.
22. Kwok CS et al. Rates, predictors and the impact of cannabis misuse on in-hospital outcomes among patients undergoing percutaneous coronary intervention (from the National Inpatient Sample). Int J Clin Pract. 2020;74(5):e13477.
23. Malhotra K et al. Association and clinical outcomes of marijuana in patients with intracerebral hemorrhage. J Stroke Cerebrovasc Dis. 2018;27(12):3479–3486.
24. Parekh T et al. Marijuana use among young adults (18–44 years of age) and risk of stroke: a Behavioral Risk Factor Surveillance System survey analysis. Stroke. 2020;51(1):308–310.
25. Patel H et al. Substance use disorders (SUDs) and risk of cardiovascular disease (CVD) and cerebrovascular disease (CeVD): analysis of the Nationwide Inpatient Sample (NIS) database. Cureus. 2023;15(5):e39331.
26. Petitti DB et al. Stroke and cocaine or amphetamine use. Epidemiology. 1998;9(6):596–600.
27. Qureshi AI et al. Crack cocaine use and stroke in young patients. Neurology. 1997;48(2):341–345.
28. Qureshi AI et al. Cocaine use and the likelihood of nonfatal myocardial infarction and stroke: data from the Third National Health and Nutrition Examination Survey. Circulation. 2001;103(4):502–506.
29. Qureshi AI et al. Cocaine use and hypertension are major risk factors for intracerebral hemorrhage in young African Americans. Ethn Dis. 2001 Spring-Summer;11(2):311–319.
30. Rezvani MR, Ghandehari K. Is opium addiction a risk factor for ischemic heart disease and ischemic stroke? J Res Med Sci. 2012;17(10):958–961.
31. Rumalla K et al. Association of recreational marijuana use with aneurysmal subarachnoid hemorrhage. J Stroke Cerebrovasc Dis. 2016;25(2):452–460.
32. Rumalla K et al. Recreational marijuana use and acute ischemic stroke: a population-based analysis of hospitalized patients in the United States. J Neurol Sci. 2016;364:191–196.
33. Saadatnia M et al. Opioid use disorder and intracerebral hemorrhage in Isfahan, Iran: a case-control study. Front Neurol. 2024;15:1420675.
34. Saberi A et al. Opium consumption prevalence among patients with ischemic stroke compared with healthy individuals in Iran. Int J High Risk Behav Addict. 2017;6(1):e27264.
35. San Luis CV et al. Association between recent cannabinoid use and acute ischemic stroke. Neurol Clin Pract. 2020;10(4):333–339.
36. Satish S et al. Urine drug screen positive for cocaine and amphetamine is not an adverse risk factor for cardiovascular morbidity or mortality in trauma. Trauma Surg Acute Care Open. 2021;6(1):e000749.
37. Shah S et al. Association of marijuana use and cardiovascular disease: a Behavioral Risk Factor Surveillance System data analysis of 133,706 US adults. Am J Med. 2021;134(5):614–620.e1.
38. Vaidya A et al. Stroke risk in unstably housed women: the role of cocaine and alcohol co-use. Stroke. 2025 Mar 20;56(5):1218-23.
39. Vin-Raviv N et al. Marijuana use and inpatient outcomes among hospitalized patients: analysis of the nationwide inpatient sample database. Cancer Med. 2017;6(1):320–329.
40. Westover AN et al. Stroke in young adults who abuse amphetamines or cocaine: a population-based study of hospitalized patients. Arch Gen Psychiatry. 2007;64(4):495-502.
41. Winhusen T et al. The association between regular cannabis use, with and without tobacco co-use, and adverse cardiovascular outcomes: cannabis may have a greater impact in non-tobacco smokers. Am J Drug Alcohol Abuse. 2020;46(4):454–461.
42. Winhusen T, Theobald J, Kaelber DC, et al. The association between regular cocaine use, with and without tobacco co-use, and adverse cardiovascular and respiratory outcomes. *Drug Alcohol Depend* 2020; 214:108136.
